# Single-Molecule 3D Images of “Hole-Hole” IgG1 Homodimers by Individual-Particle Electron Tomography

**DOI:** 10.1038/s41598-019-44978-7

**Published:** 2019-06-20

**Authors:** Dongsheng Lei, Jianfang Liu, Hongbin Liu, Thomas E. Cleveland, John P. Marino, Ming Lei, Gang Ren

**Affiliations:** 1 The Molecular Foundry, Lawrence Berkeley National Laboratory, Berkeley, CA 94720 USA; 2 Protein Analytical Chemistry, Genentech Inc., South San Francisco, CA 94080 USA; 3 Institute for Bioscience and Biotechnology Research, National Institute of Standards and Technology and the University of Maryland, Rockville, MD 20850 USA

**Keywords:** Electron microscopy, Molecular imaging, Electron microscopy, Protein design, Electron microscopy

## Abstract

The engineering of immunoglobulin-G molecules (IgGs) is of wide interest for improving therapeutics, for example by modulating the activity or multiplexing the specificity of IgGs to recognize more than one antigen. Optimization of engineered IgG requires knowledge of three-dimensional (3D) structure of synthetic IgG. However, due to flexible nature of the molecules, their structural characterization is challenging. Here, we use our reported individual-particle electron tomography (IPET) method with optimized negative-staining (OpNS) for direct 3D reconstruction of individual IgG hole-hole homodimer molecules. The hole-hole homodimer is an undesired variant generated during the production of a bispecific antibody using the knob-into-hole heterodimer technology. A total of 64 IPET 3D density maps at ~15 Å resolutions were reconstructed from 64 individual molecules, revealing 64 unique conformations. In addition to the known Y-shaped conformation, we also observed an unusual X-shaped conformation. The 3D structure of the X-shaped conformation contributes to our understanding of the structural details of the interaction between two heavy chains in the Fc domain. The IPET approach, as an orthogonal technique to characterize the 3D structure of therapeutic antibodies, provides insight into the 3D structural variety and dynamics of heterogeneous IgG molecules.

## Introduction

Immunoglobulin-G molecules (IgGs) are the predominant component of humoral immunity. In clinical practice, recombinant IgGs have been used to treat a wide array of diseases, including cancer^1–3^, rheumatoid arthritis^4–6^, and many auto-immune diseases^7–10^. Recently, advances in IgG engineering have enabled the development of monoclonal antibodies with the potential to recognize more than one antigen, such as bispecific and multi-specific antibodies^11–13^. Among them, bispecific antibodies are being evaluated for the treatment of cancer through activation of T-cell killing^14–16^ and potential reduction of the non-target toxicities of loaded antibodies such as antibody-drug conjugates (ADCs)^17,18^. Over the past 20 years, many platforms have been designed and evolved to produce bispecific IgG, such as strand-exchange engineered domain (SEED)^19^, electrostatic steering^20^, IgG4 Fab-arm exchange^21^, Diabodies^22^, and CovX-Bodies^23,24^. However, the main challenge remains to improve the yield of heterodimer and suppress homodimerization. One approach to address this challenge is the “knob-into-holes” system that involves a “knob” mutation (T366W) in one-half of the Fc C_H_3 domain and “hole” mutations (T366S, L368A and Y407V) in the other half. This system promotes hetero-dimerization through the pairing of the “knob” and the “hole”. Thus far, this system has been successfully implemented in the production and purification of several therapeutic bispecific antibodies at various clinical stages^25,26^.

The main variants generated from this system are hole-hole homodimers and, to a lesser extent, knob-knob homodimers, which are both product-related impurities and should be mostly removed through assembly and purification steps. To evaluate the performance of the purification, the levels of these homodimers were monitored. These homodimers showed unexpected behavior not normally observed in conventional monoclonal IgG antibodies (mAbs)^27^, such as a pH-dependent interchange between several peaks, which were separated on hydrophobic interaction chromatography and represented different forms of the hole-hole homodimer with the same mass^27^. The unexpected behavior of the hole-hole homodimers are related to the mutations in the C_H_3 domain and challenge the development of suitable analytical methods to monitor and understand the difference in the homodimer forms. Therefore, various modes of chromatography, native mass spectrometry (MS) and hydrogen-deuterium exchange MS (HDX-MS) were used to identify the structural basis for the pH-dependent interchange of the hole-hole homodimer forms^27^. The analytical results indicate that higher-order structure-related changes are responsible for the different hole-hole homodimer forms. The HDX-MS data showed a local increase in deuterium exchange in the C_H_2 and C_H_3 regions, but understanding its impact on the overall conformation of the homodimer will require further study. Additional higher-order structure differences that were not observed by HDX-MS may remain to be discovered.

The traditional methods used to determine the three-dimensional (3D) structure of proteins include X-ray crystallography and nuclear magnetic resonance (NMR) spectroscopy. Each method has certain disadvantages: flexible molecules such as IgGs are difficult to crystallize and IgGs are also too large to be amenable to *de novo* NMR structural determination. In recent years, single particle cryo-electron microscopy (cryo-EM) has become an important technique to determine the averaged 3D structure of a type of protein^28,29^.

Single particle cryo-EM now has the capability for structure determination of protein at atomic resolution under near-native buffer conditions^30^. However, this method also has its own limitations, such as difficulties in imaging small proteins (<50 kDa) and in achieving low-resolution 3D maps of flexible macromolecules. These flexible macromolecules include DNA and RNA, lipoprotein, antibodies^31^ and human immunodeficiency virus among many others. The problem for imaging using this technique originates from the averaging concept, in which the tens of thousands and more atoms within each particle are assumed to have identical arrangements. This assumption is largely suitable for rigid particles but becomes less so with increasing conformational flexibility and dynamics. Averaging over hundreds of thousands of particles can often cause anisotropic resolution^32^ and the loss of domains^28^ in the final 3D reconstruction, although other portions of the molecule may show atomic resolution. Moreover, even in cryo-EM, sample preparation artifacts can be present. The thin water film used for cryo-EM specimens can often cause proteins to adopt a preferred orientation in the ice, which can be unduly influenced by the air-water interface^33^. In addition, the air-water interface can induce proteins to denature or aggregate, and complexes to dissociate^34^. In comparison, although negative staining (NS) remains useful as a supplementary method to study small and flexible proteins at a low-resolution, but with high contrast. Some disadvantages of NS remain, such as stain artifacts, complex dissociation, stain-induced chemical reactions and substrate interaction. Therefore, no sample preparation method is perfect.

Preliminary NS-EM 2D images showed that the hole-hole homodimers are heterogeneous in structure^27^. Unlike in the conventional Y-shaped conformation, it was observed that in some particles, the C_H_2 and C_H_3 domains of the Fc of the homodimers were distant from each other^27^, displaying an “X-shaped” conformation. However, geometric analysis based on these 2D images was insufficient to uncover the detailed 3D structure or the extent of the structural flexibility.

To obtain the 3D structure of the hole-hole homodimers, the optimized negative-staining (OpNS)^35–38^ method was used to prepare the EM specimen, and both conventional single-particle class averaging and then the individual-particle electron tomography (IPET) method^24,39,40^ was used to obtain the 3D reconstructions. Reconstructing 64 IPET 3D density maps of the IgG hole-hole homodimers at a resolution of ~15 Å were used to derive 64 conformations of the IgG homodimers via flexible docking (“hand-in-glove”) of a crystal structure of IgG (PDB entry 1HZH^41^) into the envelope of each IPET 3D density map. Additional statistical analysis on the geometry of the IgG homodimers (domain distance and angle) investigated the conformational variety and flexibility among the domains and provided a mean to quantitatively understand the differences in structural flexibility between a regular antibody and the hole-hole homodimer.

## Results

### Morphology of IgG hole-hole homodimers

**Figure 1:**
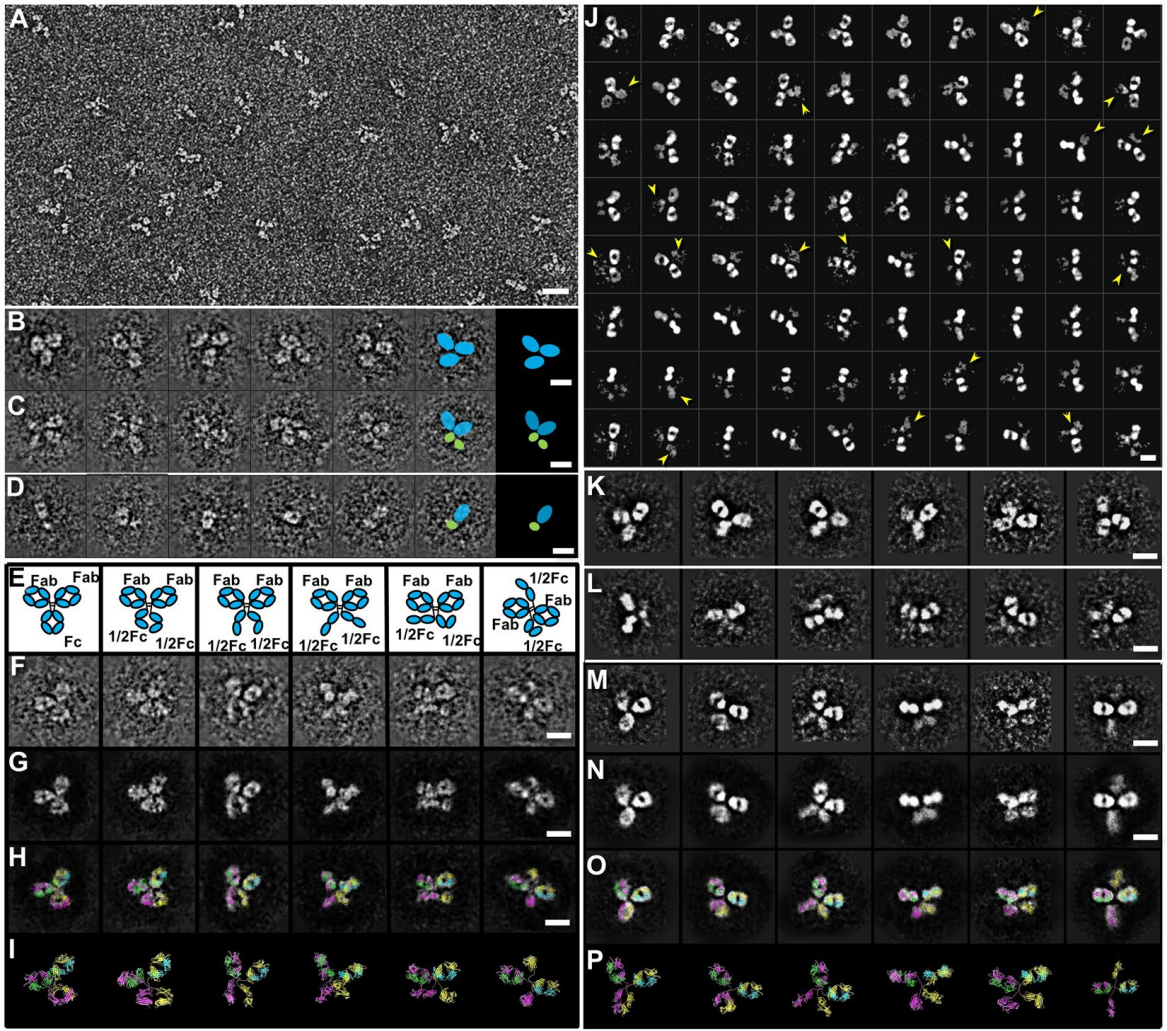
OpNS-EM images of IgG hole-hole homodimer sample. (**A**) A survey OpNS image of the IgG hole-hole homodimer sample. Representative images of (**B**) Y-shaped, (**C**) X-shaped, and (**D**) i-shaped particles are shown. The last images of the Y-, X-, and i-shaped particles were superposed with diagrams to show the overall location of large domains (cyan ellipses) and small regions (green ellipses) within each particle. (**E**) Diagram of various conformations of IgG homodimer. (**F**) Representative raw images showing conformations in (**E**). (**G**) After erasing the noise areas outside particles, (**H**) images were superposed by using an IgG model (crystal structure of regular IgG, PDB entry 1HZH) to show the overall location of domains. (**I**) Obtained models show various conformations of IgG homodimer. All models are shown in ribbon representation, in which heavy chains are in yellow and magenta, and light chains are in cyan and green. (**J**) Representative reference-free class averages from 13,546 particles. Arrows indicate averages with fuzzy or blurry domains (**K**) Representative Y-shaped class averages. (**L**) Representative X-shaped class averages. (**M**) Class averages of Y- and X-shaped particles in various conformations. (**N**) After erasing noise areas outside particles, (**O**) the averages were superposed using the IgG model to show the overall location of Fab, Fc or half Fc domains (detail provided in Methods section). During superposing, the location and orientation of domains within models were required to be adjusted to fit to each average. (**P**) Obtained models show overall conformations of IgG homodimer. Scale bars = 20 nm in A and 10 nm in (**B**–**P**).

The IgG hole-hole homodimers produced as a side product from the assembly of the knob-into-hole bispecific antibody were examined by OpNS EM (Fig. 1A–D)^35–38^. The OpNS protocol was refined from conventional NS protocols by introducing three additional steps: (i) filtering the thawed staining solution (1% Uranyl formate, UF) using the smallest available filter (0.02 μm) to remove the precipitated particles immediately before usage (the newly prepared 1% UF was aliquoted into 2 mL/vial and stored at −20 °C), (ii) incubating and then staining the EM grid within a black box to prevent the stain reagent from the light-exposure, and (iii) drying the specimen with nitrogen gas to prevent sample oxidation. The OpNS protocol can limit certain artifacts, such as Rouleau formation for lipoprotein particles^35–38^, and has been used to examine many biological macromolecules^24,27,35–38,40,42–62^, including antibodies^24,27,49,53^, cholesteryl ester transfer protein (CETP)^46^, phospholipid transfer protein (PLTP)^63^, liposome-CETP complex^42^, lipoproteins and their antibody complexes^35,38,43,58^, GroEL and Proteosome^37^, Contactin-associated Protein-like 2 (CNTNAP2)^59^, Calsyntenin-3^40^, Neurexin 1𝛼^51^, 84-base pair dsDNA conjugated with 5 nm nanogold^57^, and DNA origami^52^. These studies demonstrate that OpNS is a suitable and reliable protocol to examine the structure and morphology of small, and/or flexible proteins.

OpNS-EM images of the IgG hole-hole homodimer showed evenly distributed particles (Fig. 1A, after bandpass filtering between 8 Å and 2,000 Å). Most particles had a dimension of ~150–190 Å, which is similar to that of regular IgGs^49^. Zoom-in images showed that most particles have a Y-shape (~60.6%) (Fig. 1B). Unexpectedly, many particles (~26.9%) displayed as X-shape (Fig. 1C, Supplementary Video 1), bow-tie shape (~10.0%) or even i-shape (~2.5%) (Fig. 1D). The Y-shaped particles had a similar structure to regular IgGs^49^, while the X-shaped particles contained four apparent domains: two large ring-shaped domains and two small rod-shaped domains (Fig. 1E). The two ring-shaped domains, likely to be Fab domains, were similar to each other in size (overall diameter) and shape (aspect ratio). The two small rod-shaped domains were similar to each other and were likely the C_H_2 and C_H_3 domains of the heavy chain, *i*.*e*., an Fc domain in which the two heavy chains were separated from each other (Fig. 1E). Two domains of the bow-tie shaped particles have a similar size and shape to each other and Fab domains, while the Fc domain is apparently absent, possibly denatured. The i-shaped particles were similar to half of an X-shaped particle in size and shape, likely composed of a Fab domain and half of an Fc domain (Fig. 1E–I).

To evaluate whether the observed X-shaped particle was statistically significant in its abundance, we performed a reference-free classification based on 13,546 particles. The high-contrast 2D class averages confirmed all Y-shaped, X-shaped, i-shaped and bow-tie shaped particles (Fig. 1J–L). The difference between the Y- and X-shaped particles was within the conformation of the Fc domains, in which one of three domains in the Y-shaped particles converted into two small rod-shaped domains in the X-shaped particles.

The size and shape of domains within Y- and X-shaped class averages were similar to those of an IgG crystal structure (PDB entry 1HZH^41^), except for the split Fc domain of the X-shaped averages. The similarity allowed us to map 3D models to the 2D class averages following methods previously used in EM^64–67^ and AFM^68,69^. Although the class averages are insufficient for determining the 3D particle structures, they help us to understand the overall shape and conformational variety in terms of the domain locations (Fig. 1M–P). During the mapping, we found that no matter how we changed the orientations of the antibody, the projection of the model could not match well with some Y-shaped and all X-shaped averages, unless we adjusted the structure of the hinge region to allow the domains to move and change their orientations. The new locations and orientations allowed us to match the Y-shaped particle easily (Fig. 1M–P, first column). For X-shaped particles, an additional operation was needed; *i*.*e*., the two halves of the heavy chains of the Fc domain (two copies of C_H_2 and C_H_3 domains) must be separated from each other to match to the rod-shaped densities in the 2D averages of the X-shaped particles (Fig. 1M–P, last four columns). The wide range of orientations between the two copies of the C_H_2 and C_H_3 domains of the Fc suggested that X-shaped particles were more flexible than Y-shaped particles. Similarly, the above mapping method was also applied to the raw images of Y-shaped and X-shaped particles to confirm the flexibilities of particles (Fig. 1F–I).

### Evaluation of potential artifacts from NS and substrate interactions using the NISTmAb

Although the OpNS protocol has been used to examine more than 20 types of macromolecules for high-contrast imaging^37,53^, one may still question whether the antibody-substrate interaction or the low pH of the uranyl formate NS causes noticeable artifacts. These artifacts could include conformational changes or the domain dissociation observed in the X-shaped particles.

In principle, cryo-EM is the ideal approach to examine the proteins in near-native solution conditions without artifacts from NS. Practically, however, it is very challenging to image the antibody by itself (not antibody-antigen complex) due to its low molecular weight and floppy structure (three relatively isolated ~50 kDa domains linked by a flexible hinge region). Generally, proteins of molecular weight less than 50 kDa are beyond current limitations of cryo-EM structural determination. Although antibodies comprise more than 10% of human plasma proteins, and are highly desired for pharmaceutical drug design, no cryo-EM 3D reconstruction has been reported according to our best knowledge. While cryo-electron tomography (ET) 3D reconstructions have been reported^70,71^, no raw data or intermediate results have been shown in the publications. Moreover, one publication using the same approach was retracted due to low reliability (more than 90% of the reconstructions were invalid based on third-party evaluation using IMOD reconstructions)^72,73^. Thus, cryo-EM is still challenging as a practical approach for studying antibody structure. We therefore supported our NS methodology using identical studies of a control antibody.

**Figure 2:**
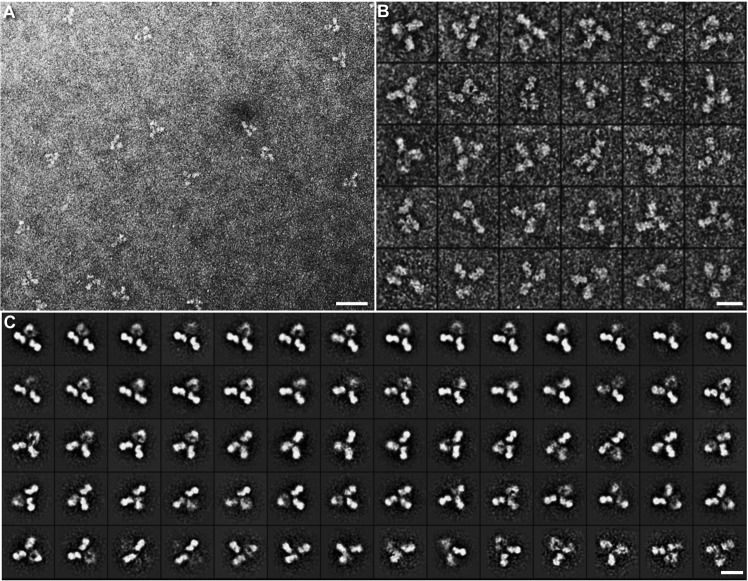
OpNS-EM images of NISTmAb sample. (**A**) A survey OpNS image of NISTmAb sample. (**B**) Representative images of particles, and (**C**) representative class-averages from 4,961 particles. Scale bars = 30 nm in **A**, 10 nm in (**B**,**C**).

To examine whether the X-shaped particles were related to artifacts from the NS or substrate interaction, we used the same NS protocol and supporting substrate to examine the NISTmAb, a standard monoclonal antibody provided by National Institute of Standard Technology (NIST), which is available as a reference material (RM 8671)^74^. NISTmAb is an IgG1 antibody performance standard useful for evaluation/development of state-of-the-art and emerging analytical measurement technologies^75,76^. The material has been used extensively to evaluate current best practices for mAb characterization and develop innovative analytical technologies. The survey image showed evenly distributed NISTmAb particles with a “Y” shape with dimensions of ~150–180 Å (Fig. 2A). The particles were composed of three ring-shaped domains of ~55–75 Å in diameter (Fig. 2B), which were similar to those of crystal structures (PDB entry, 1HZH^41^). No X-shaped particle was observed. The reference-free class averages from ~4,961 particles confirmed that a “Y”-shaped structure was the only species in the sample (Fig. 2C). The result was consistent to the early reports; *i*.*e*., no X-shaped particles were observed in other IgG samples by using same NS protocol^24,27,37–39^. Although the pH of the staining is as low as ~4.5, which is different from the pH of the samples, it has been reported that UF can fix protein structure within a few milliseconds^77^, which may explain the lack of apparent of pH induced artifacts.

Although we could not exclude the possibility that the flexibility of antibodies could be influenced by the substrate interactions, we find it unlikely that the X-shaped antibody was solely an artifact of NS, and conclude that the X-shape is an intrinsic conformation of the hole-hole IgG homodimer. This result is also consistent with multiple orthogonal methods, including chromatography, native MS and HDX-MS^27^.

### The limitations of conventional single-particle averaging in 3D reconstruction

To understand the detailed structure of the X-shaped particles, a 3D structure was required as projections were often insufficient. Although single-particle class averaging is the dominant technique for 3D structural determination, the method has limitations in the reconstruction of flexible proteins. This is because it requires not only an initial model but also the assumption of identical particles in 3D structure (or at least, a small number of discrete structures)^78^. Using this averaging method, the 3D reconstructions were either significantly dependent on the given initial models or gave structurally impossible solutions^49^. To demonstrate the limitations of single-particle reconstruction on the hole-hole homodimer, we used the EMAN software package^78^ for reconstruction. Since the underlying methodology of single-particle reconstruction is similar in other software packages, the phenomenon illustrated in the example is also expected from other software packages.

For 3D reconstruction by single-particle averaging, two types of initial models were used: a cluster of ellipsoids representing domains  and a single Gaussian blob representing an entire antibody (Supplementary Fig. S1)^78^. In the domain models, four arrangements of ellipsoids were tried: two models consisted of three ellipsoidal blobs forming a Y-shape with angles of 150° and 45° respectively (Supplementary Fig. S1A, left two); while the other two models consisted of two large and two small ellipsoidal blobs forming an X-shape and cross-shape, respectively (Supplementary Fig. S1A, right two). In the full-antibody models, the initial models consisted of a single featureless ellipsoidal Gaussian blob at four noise levels (Supplementary Fig. S1C).

Through a multi-reference refinement process, the final single-particle 3D reconstructions achieved from the domain models showed domains having similar angles to the initial models (Supplementary Fig. S1B), suggesting an initial model bias. The final 3D reconstructions from the full-antibody models showed significantly different structures from the initial models, but none of them was consistent with the crystal structure (PDB: 1HZH). This result demonstrates the limitations of single-particle 3D reconstruction on flexible proteins is consistent with the problems reported in previous publications on IgG1 molecules^49^. Furthermore, these phenomena are anticipated for single-particle 3D reconstructions regardless of which software packages are used. It has been reported that common artifacts in single-particle 3D reconstruction (averaging) include the presence of blurry domains^79^, smaller than the expected dimensions of domains, uneven distribution of resolutions^32^, and the absence of protein domains/regions^80^, such as two ankyrin repeat regions, which were absent in the atomic structure of TRPV1^28^. Given the limitations of the single-particle averaging reconstruction method in the structural determination of flexible proteins, we studied the hole-hole homodimer using the IPET approach, which has been tested on small and flexible macromolecules^24,39,40,42,49–55,57–59,62^.

### IPET 3D reconstruction of individual particles

**Figure 3:**
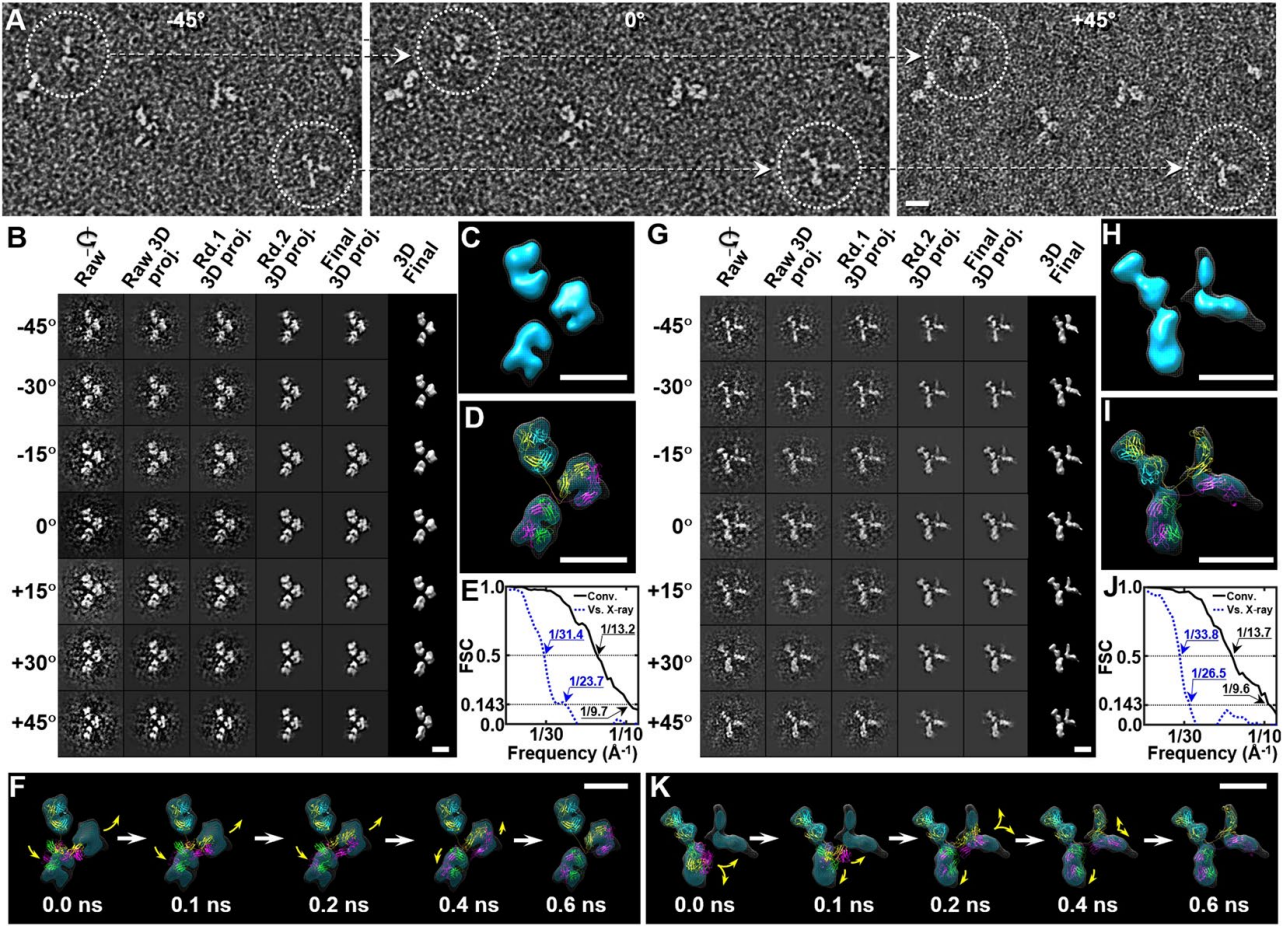
3D reconstruction of two representative IgG homodimers by IPET. (**A**) OpNS samples of the IgG homodimer were imaged using ET from a series of tilt angles (from −45° to +45° at 1.5° intervals). Two homodimer particles (white circled) with orthogonal views are indicated by linked dashed arrows in three selected ET tilt micrographs. (**B**) Seven representative tilt images of an individual Y-shaped particle are displayed in the first column from the left. Using IPET, the tilt images (after CTF correction) were gradually aligned to a common center for 3D reconstruction via iterative refinement. Projections of raw, intermediate and final 3D reconstructions at corresponding tilt angles are displayed in the next five columns according to their corresponding tilt angles. (**C**) Final IPET 3D density map of targeted individual particle. The density map was low-pass filtered to 20 Å to reduce over-interpretation of high frequency or local noise. The ‘hide dust’ function was applied by Chimera. (**D**) The final 3D density map was flexibly docked with IgG crystal structure by using TMD simulation. (**E**) The resolution of IPET reconstruction was estimated by two Fourier shell correlation (FSC) curves. The first FSC curve (black solid line) was computed from two 3D maps that were back-projected from two sets of self-aligned tilt images (with even and odd tilt order numbers). The FSC curve fell below 0.5 at ~13.2 Å, and fell below 0.143 at ~9.7 Å. The second FSC curve (blue dash line) was computed from the IPET reconstruction and the atomic resolution density map calculated from the fitted model. The FSC curve fell below 0.5 at ~31.4 Å and 0.143 at ~23.7 Å. (**F**) Five snapshots illustrated the conformational changes of the IgG model during TMD simulation. (**G**–**K**) The 3D density map of a second individual IgG homodimer was reconstructed from the tilt images using IPET. FSC analysis between the reconstructions from odd and even tilt images showed that FSC curve fell below 0.5 at ~13.7 Å and fell below 0.143 at ~9.6 Å (black solid line). The FSC analyses between the IPET final map and the map from the fitting model showed the FSC curve fell below 0.5 at ~33.8 Å and fell below 0.143 at ~26.5 Å (blue dash line). IgG models are shown in ribbon representation, in which heavy chains are in yellow and magenta, and light chains are in cyan and green. Scale bars = 10 nm.

**Figure 4:**
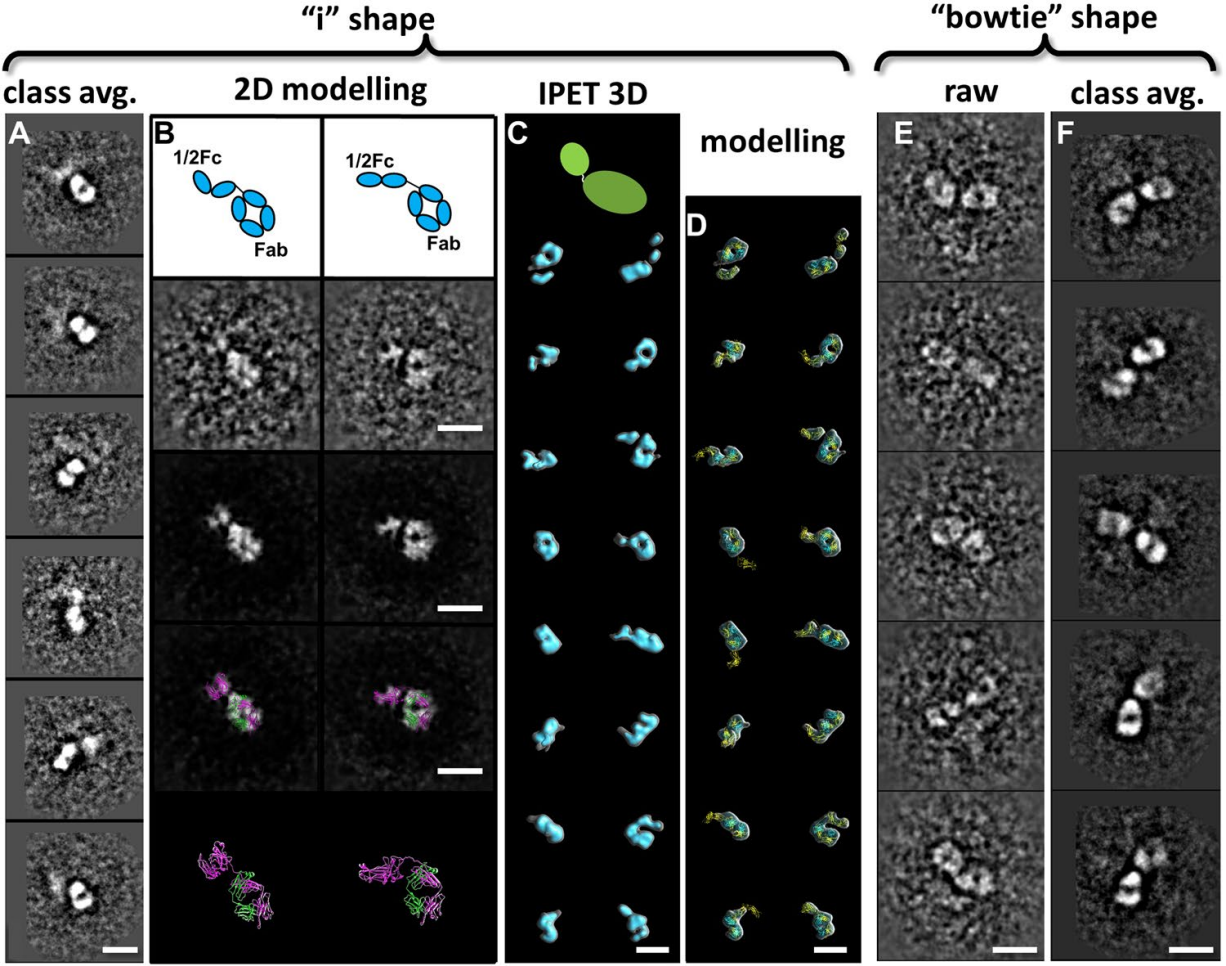
Minor species, i-shaped and bow-tie-shaped particles, presented in IgG homodimer. (**A**) Six representative class averages of i-shaped particle based on OpNS images. (**B**) Two representative i-shaped particles were superposed by using a half-IgG model. (**C**) Sixteen density maps of i-shaped particle were reconstructed by using IPET. These density maps are presented in double contours. The inner contours are shown in cyan; outer contours are shown in transparent gray. (**D**) The density maps were flexibly docked with half-IgG models. (**E**) Five representative bow-tie-shaped particles. (**F**) Five representative bow-tie-shaped class averages. Scale bars = 10 nm.

The IPET 3D reconstruction method was used to obtain the 3D map from each individual particle of the X-shaped, Y-shaped and i-shaped isomers (Figs 3 and 4). This method has demonstrated its ability to reconstruct 3D structures of flexible proteins^49,52,57–59^, including IgG1^49^, peptide-conjugated IgG1^24^, cholesteryl ester transfer protein (CETP) bound to lipoprotein^54^, and antibody bound to lipoproteins^58,81^. In IPET approach, particles were imaged from a series of tilt angles from −45° to 45° in 1.5° increments by electron tomography (ET) (Fig. 3A). The targeted particle was tracked and selected from each tilt series after contrast transfer function (CTF) correction. Unlike the single-particle 3D reocnstruction (an averaging approach), no initial model was required. The first *ab initio* 3D reconstruction was directly generated from the experimental tilt images via a back-projection algorithm. These tilt images were iteratively aligned to their global centers to achieve a final 3D reconstruction via IPET approach. During the iterative alignment process, automatically generated Gaussian low-pass filters and particle shaped soft-boundary masks were used to reduce the noise. A missing-wedge correction was also applied during the process (see Methods). The step-by-step refinement procedures and intermediate results are shown in Fig. 3B. The final 3D density map showed an overall Y-shaped particle (Fig. 3C) at a resolution of ~13.2 Å (Fig. 3E) based on the Fourier shell correlation (FSC) analysis as described^49^.

In the IPET reconstructions, the Y-shaped particle had an overall diameter of ~195 Å, and two of the three domains were similar to each other in size and shape (approximately 90 Å in diameter), suggesting that they were Fab domains. The third domain, which was different from other two domains in size, was likely the Fc domain. This IPET structure was consistent with the IgG crystal structure in overall size, domain shape and domain size, which allowed us to determine the overall particle conformation by flexibly docking the crystal structure (PDB: 1HZH) into the map (details provided in the Methods section). To understand molecular conformation, we used an established method, flexible fitting of the high-resolution crystal structure into the low-resolution EM density map using molecular dynamics^82–89^. Although the docked model could not reveal the atomic resolution structure, it was sufficient to reveal the low-resolution conformation^82,89,90^.

During the docking process, which was similar to what has been reported^49^, the IPET 3D map was used as a constraint. The Fab domains and half Fc domain of the crystal structure were treated as rigid bodies to be oriented and inserted into the EM envelope through the rotation operator, maximizing the overlap between the domain structure and map (notably, the heavy chain and light chain in Fab domains are not distinguishable at the current resolution). The hinge region was treated as a flexible structure to respond to the domain position changes, but under the constraints of chemical bonds and energy minimization. The final 3D conformation of the IgG homodimer was achieved by targeted molecular dynamics (TMD) simulations, maximizing the overlap between the structure and the density map while minimizing the energy of the structure. The quality of the fitting to the envelope was evaluated by the FSC curve computed between the model and density map (Fig. 3E,J).

By repeating the IPET 3D reconstruction on an X-shaped particle, we reconstructed a 3D map at ~13.7 Å resolution (Fig. 3G–K, Supplementary Video 1). The intermediate results of the iterative tilt series alignment (Fig. 3G) and the final 3D reconstruction showed a particle with an overall diameter of ~190 Å, containing two large dumbbell-shaped domains and two smaller domains (Fig. 3H). The two large domains, with similar lengths of ~80–90 Å and widths of ~40–50 Å, were likely the Fab domains. The small domains, with a length of ~80 Å and width of ~30 Å, were likely to be two halves of the Fc domain.

After flexibly docking the crystal structure into this map, the 3D conformation of the X-shaped particle (Fig. 3I,K, Supplementary Video 1) showed that the two halves of the Fc domain formed an angle of ~100° with a distance between the far ends of ~44 Å. This distance was rather large compared to the distance between two C_H_3 domains in the Y-shaped particle, where two C_H_3 domains were attached to each other. This large distance between two C_H_3 domains suggested a weak interaction.

By further repeating the above process, a total of 80 particles were targeted for IPET 3D reconstruction from a pool of ~250 particles acquired from four tilt series (Figs 3, 4 and 5, Supplementary Figs 2–45, Supplementary Table 1). A large number of particles were excluded due to particle-particle overlapping at certain tilt angles, missing tilted views, uneven surrounding stain backgrounds or already sufficient examples to present each species. The 80 IPET 3D maps included 16 maps from the Y-shaped particles, 48 maps from the X-shaped particles and 16 maps from the i-shaped particles. The map resolutions were within a range of ~12 to ~15 Å. By flexibly fitting the crystal structure into these maps, we obtained 16 conformations for Y-shaped antibodies (Fig. 5B, Supplementary Figs 2–11), 48 conformations for X-shaped antibodies (Fig. 5D, Supplementary Figs 12–35) and 16 conformations for half antibodies (Fig. 4A–D, Supplementary Figs 36–45).

**Figure 5:**
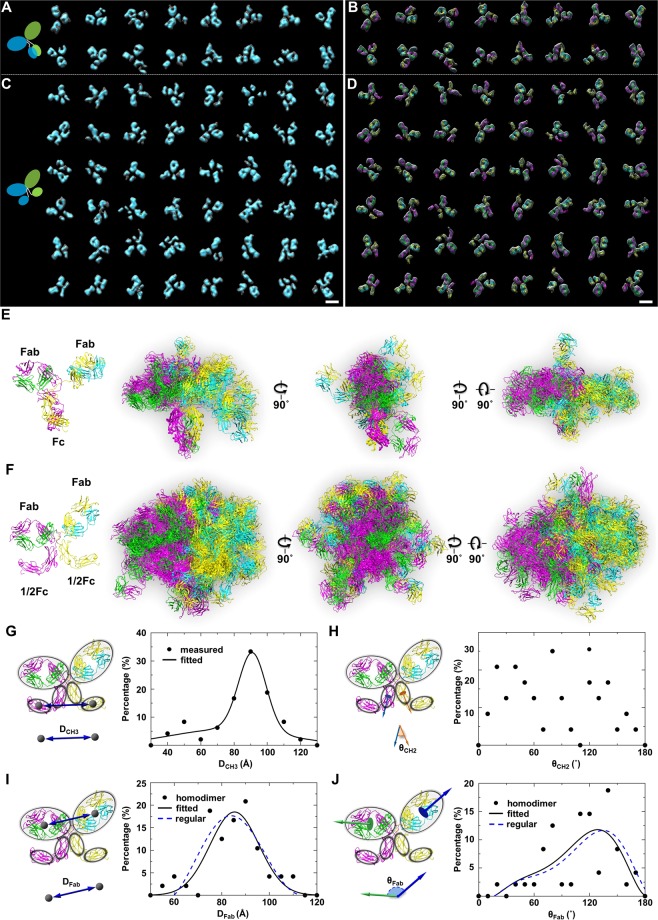
3D conformation and conformational flexibility of IgG homodimer. (**A**) Sixteen density maps of Y-shaped IgG homodimers by IPET. (**B**) The density maps were flexibly docked using the crystal structure of a IgG to determine the overall conformations of corresponding maps. (**C**) Forty-eight density maps of X-shaped IgG homodimers by IPET. (**D**) The density maps were flexibly docked using the crystal structure of a IgG to determine the overall conformations of corresponding maps. The density maps are presented in double contours. The inner contours contoured surfaces are shown in cyan; outer contours are shown as transparent gray surfaces. (**E**) Sixteen Y-shaped conformations were aligned based on their C_H_2 domains. (**F**) Forty-eight X-shaped conformations were aligned based on their C_H_2 domains. Distributions of Y- and X-shaped IgG homodimers are shown from three orthogonal views. (**G**) Schematic model illustrating the distance between centers of C_H_3 domains (left) and the histogram of measured distance from 48 X-shaped homodimers (right). The histogram was fitted by using 2-term Gaussian model. (**H**) Schematic model illustrating the angle between the C_H_2 domains (left) and the histogram of the measured angle (right). (**I**) Schematic model illustrating the distance between centers of Fab domains (left) and the histogram of the measured distance (right). The histogram was fitted by using Gaussian model, and compared with the distance curve reported for Y-shaped IgG^49^. (**J**) Schematic model illustrating the angle between Fab domains (left) and the histogram of the measured angle (right). The histogram was fitted by using sixth degree polynomial curve and compared with the angle curve reported for regular IgG^49^.

### Resolution analysis on IPET 3D reconstruction

The resolution of IPET reconstruction was estimated by the following methods as reported^49^. (i) The single-particle method^91,92^, in which the resolution of the IPET reconstruction was estimated based on the matching of data to themselves by splitting the tilted images into two halves for reconstruction independently. The points where the FSC curve fell below 0.5 were used to represent the reconstruction resolution^91,92^. In this case, the resolution of the first reconstructed Y-shaped antibody was ~13.2 Å, while that of the first reconstructed X-shaped antibody was 13.7 Å (black solid lines in Fig. 3E,J). However, if we chose the “gold-standard” criteria^93^, *i*.*e*. using the point where the FSC curve fell below 0.143 as the resolution, the resolution of the Y-shaped antibody was ~9.7 Å, while that of the X-shaped antibody was ~9.6 Å (black solid lines in Fig. 3E,J). (ii) A model-to-map method, in which the resolution was estimated based on the matching of data to the best fitted model. The 3D density map of the best fitted structure using *pdb2mrc* software (EMAN package)^78^ was used to calculate the FSC curve against the IPET 3D map. The resolutions at which the FSC curves fell below 0.5 were ~31.4 Å for the Y-shaped antibody and ~33.8 Å for the X-shaped antibody (blue dash line in Fig. 3E,J). If we used “gold standard” criteria^93^, the resolutions were ~23.7 Å for the Y-shaped antibody and ~26.5 Å for the X-shaped antibody. Notably, because the domains in the crystal structure were docked into the IPET maps as rigid bodies, the flexibility within the Fab and half Fc domains was not included in the resolution estimation, which may lead to an underestimated resolution. iii) A structural feature comparison method. The structural features included the maps of two heavy chain segments (C_H_2 and C_H_3) in the Fc domain. Since the dimension of C_H_2/C_H_3 was ~17 × ~18 × ~35 Å, the success in reconstructing the domains suggested the resolutions were roughly ~20 Å, consistent with the previous study of IgG1^49^. To simplify estimation, we chose the frequency at which the FSC curve from single particle method fell below 0.5 (instead of 0.143) as an estimate of the resolution, as the previous publications^24,39,40,49,52,57–59^.

### Statistical analysis of the conformational flexibility of 64 conformations

To reveal the flexibility of the newly found X-shaped particles, the 48 X -shaped particles were aligned to each other based on the structure of their C_H_2 domains. This alignment yielded an overall ball-like distribution in which both Fab domains and Fc domains adopted a wide range of angles and orientations (Fig. 5F). Compared to the aligned 3D conformations of the Y-shaped particles (Fig. 5E), the Fc domains in the X-shaped particles presented with a wider distribution of spatial orientations, suggesting that the X-shaped particles have increased flexibility, particularly in the Fc domain.

To quantify the conformational flexibility of the Fc domains, we analyzed the distribution of distances between the centers of the C_H_3 domains and the distribution of angles between the C_H_2 domains of the 48 X-shaped particles. The histogram of the distances (fitted by a 2-term Gaussian model) showed that ~72.1% of the X-shaped particles have C_H_3 distances ranging from 70–110 Å (Fig. 5G). The angles measured between two C_H_2 domains were distributed in a range of 0° to 180° (Fig. 5H). The histogram of the angles showed no obvious peak population, suggesting that two halves of the Fc domains can move and rotate freely, with little constraint or force against each other.

Likewise, to quantify the conformational flexibility between two Fab domains of X-shaped particles, and to compare it to that of regular Y-shaped particles reported before^49^, the distances and angles between Fab domains were measured. The histogram of the distances from ~50 to ~115 Å (Fig. 5I, fitted by a 1-term Gaussian model) showed a peak population at a distance of ~85 Å. The histogram of the angles from ~10° to 180° (Fig. 5J, mostly asymmetrical, therefore fitted by a sixth-degree polynomial curve) showed a peak at ~130°. The distributions of distances and angles were similar to those of the published IgG1^49^ by IPET (Fig. 5I,J, dashed lines). In addition, the peak distance and angle of the X-shaped particles (~85 Å and ~130°) were similar to the distance and angle measured between two Fab domains in the IgG1 crystal structure (~89 Å and ~140°, PDB entry 1HZH). These results suggested that the flexibility of the two C_H_3 domains within the Fc domain does not influence the flexibility of the two Fab domains. This may result from the highly flexible hinge region that decouples conformational changes in the Fc domain from the F domains.

## Discussion

EM images give insight into the structural variety of the IgG homodimer, especially the interactions between the heavy chain segments forming the Fc domain (C_H_2 and C_H_3 domains). In addition to the Y-shaped conformations seen in the reported IgG1 molecules^49^, X-shaped conformations were also observed, in which the two heavy chain segments of the Fc domain are apart. These results are also consistent with previous biophysical studies^27^.

### Structural interpretation of the interaction between two C_H_3 chains

The IPET reconstructions show that the interaction between the two halves of the Fc domain in X-shaped IgG hole-hole homodimers is weakened compared to that of Y-shaped particles, which allows the two halves to move freely. To aid in the structural interpretation of this observation, we used the crystal structure of an IgG1 (PDB: 1HZH) as a template, as well as an Fc fragment with hole-hole mutations (PDB entry 4NQT)^94^ as a template to construct a homology model of the IgG hole-hole homodimer (see Methods). The crystal structure of IgG1 shows that multiple residues contribute to the interaction between two C_H_3 domains in forming the Fc domain. The interactions include (i) hydrogen bonding between T366 and Y407^95^, which are within hydrogen-bonding distance, as reported^95^; (ii) π-stacking between Y407 residues, where the benzene rings of two Y407 residues in the two halves of the Fc domain are located side-by-side with a distance of 4.31 Å, as an energetically stable conformation from π-stacking^96^; and (iii) inter-domain hydrogen bonding mediated by a network of water molecules^97^.

In the hole-hole homodimer, mutations were introduced into the Fc domain (T366S, L368A and Y407V^27^), located at the C_H_3-C_H_3 interface (Supplementary Fig. 46A). These mutations seem to reduce the interactions between two C_H_3 domains (Supplementary Fig. 46B). It is plausible that upon mutation, V407 and S366 become too far from each other to maintain the original hydrogen bond. Additionally, the mutation from tyrosine to valine removes the original π-stacking interaction between the two Y407 residues. Together, these mutations lead to reduced interactions between C_H_3 domains, thus the two C_H_3 domains can move freely from each other and lead to the observation of X-shaped particles in the homodimer. Hydrogen-deuterium exchange mass spectrometry also shows decreased protection of the same regions in the C_H_3 domains, consistent with fewer interactions^27^.

Interestingly, Y-shaped homodimer particles with intact Fc domains were still observed in the homodimer sample, suggesting that the two halves of the Fc domain still maintain some interactions after mutation, but with reduced strength. For example, the mutation of hydrophilic Y407 to hydrophobic V407 increases the hydrophobicity of the local surface (Supplementary Fig. 46C,D), and therefore may increase the nonspecific hydrophobic interactions between the two halves of the Fc domain. The observation of both X- and Y-shaped particles may relate to the equilibrium between the conformations.

### Minor components in the hole-hole homodimer sample

In addition to Y- and X-shaped particles, low percentages of bow-tie-shaped particles (~10.0%) and i-shaped particles (~2.5%) were also observed in the sample of IgG hole-hole homodimer (Figs 1D and 4A,E,F). The bow-tie-shaped particles contain two domains that are similar to each other in size and shape, and similar to the Fab domains in the Y- and X-shaped particles. However, the third domain, likely the Fc domain, is not seen. Given that physically truncated homodimers were not detected by chromatography, native MS or HDX-MS^27^, the apparently absent Fc domain is likely still present but denatured or disordered, resulting in a lack of visibility in EM images.

The i-shaped particles have an overall diameter of ~110 Å, with a similar size and shape to half of an X-shaped IgG. These particles are likely formed by Fab domain and half of an Fc domain (Fig. 4A,B), *i*.*e*. a single heavy and light chain. This hypothesis is consistent with our previous study in which a species with half of an intact IgG mass was identified in the hole-hole homodimer sample by native MS^27^. However, in that study the species observed by native MS could not be reliably detected by SEC, possibly due to its low abundance. Our 16 IPET maps of i-shaped particles showed that the particle is composed of a large and a small globular domain, which are similar to half of the X-shaped antibody in size and shape (Fig. 4C and Supplementary Figs 36–45). By flexibly fitting a half-antibody model into each map (Fig. 4D and Supplementary Figs 36–45), the agreement in overall size and shape strongly supports the hypothesis that the i-shaped particles are halves of antibodies. The observation of half-antibodies, especially the half Fc domain (approximately 25 kDa), is far below the limitations of EM imaging using other methods, suggesting that the combination of the OpNS and IPET methods can be used as a supplementary tool to study the structure of small and flexible proteins, including therapeutic IgGs.

## Conclusion

In this study, we characterized the newly discovered X-shaped particles in a hole-hole homodimer using NS-EM with IPET technology. Through the 3D reconstruction of 64 individual particles, we confirmed that the apparent X-shape was due to the weakened interaction between the two halves of the Fc domain of this homodimer. The X-shaped particle was not observed in regular IgG1 samples. The weakened interaction between the two halves of the Fc domain in the homodimer appears to be due to the mutations in the C_H_3 domain. We also observed several minor species using the IPET method. Our study demonstrates the capability of the IPET with NS-EM method in the topological study of small and flexible proteins in heterogeneous samples, as a supplementary tool for future engineering optimization of therapeutic antibodies.

## Methods

### Production of the hole−hole homodimer and NISTmAb

The hole-hole homodimer was produced as described in literature^27^. In brief, the harvested cell culture fluid containing the hole half antibody was first purified on protein-A affinity chromatography. The pH of the protein-A pool was adjusted from 3.3 to 5.0, and Poros cation exchange chromatography was used to separate the hole-hole homodimer from the half antibody and other species. The resulting solution was then eluted at pH 5.5. The solution was finally submitted to ultrafiltration/diafiltration and conditioned at pH 5.8 in 20 mM histidine acetate buffer. Intact mass analysis confirmed that the hole-hole homodimer was a covalent homodimer. The NISTmAb is a recombinant, humanized IgG1 expressed in a murine suspension culture^74^, and is available as a reference material (RM 8671). As supplied, the reference material (lot 14HB-D-002) typically contains approximately 3% oligomers (dimer/trimer) by mass. For EM studies, monomeric NISTmAb was isolated by size exclusion chromatography in phosphate buffered saline (PBS) using a Superdex 200 16/60 Prep Grade column (GE Healthcare) and flash-frozen until needed.

### Preparation of OpNS-EM specimens

The OpNS specimens of IgG hole-hole homodimers and the NISTmAb were prepared by using the protocol as described^35–38^. In brief, IgG samples were diluted to ~0.04 μg mL^−1^ with Dulbecco’s phosphate-buffered saline (DPBS). An aliquot (approximately 4 μL) of diluted sample was placed on an ultra-thin carbon-coated 200-mesh copper grid (CF200-Cu-UL, Electron Microscopy Sciences, Hatfield, PA, USA, and Cu-200CN, Pacific Grid-Tech, San Francisco, CA, USA) that had been glow-discharged for 15 s. After 1-min incubation, the excess solution on the grid was blotted with filter paper. The grid was then washed with water and stained with 1% (w/v) uranyl formate (UF) before air-drying with nitrogen.

### TEM un-tilted data acquisition and image processing

OpNS samples were examined by using a Zeiss Libra 120 Plus TEM (Carl Zeiss NTS) operated at 120 kV high tension with a 10–20 eV energy filter. The OpNS micrographs were acquired under defocus at ~0.6 μm to ~0.9 μm and a dose of ~40–90 e^−^Å^−2^ using a Gatan UltraScan 4 K X 4 K CCD under a magnification of 80 kx (each pixel of the micrographs corresponds to 1.48 Å in specimens). The contrast transfer function (CTF) of each micrograph was examined by using *ctffind3* software^98^ and corrected by using the “*TF CTS*” command in SPIDER^99^ software or GCTF^100^ after the X-ray speckles were removed. Particles were then selected from the micrographs with a box size of 192 × 192 by using *boxer* (EMAN^78^ software). All particles were masked by using a round mask generated from SPIDER software after a Gaussian high-pass filtering. The reference-free class averages of particles were obtained by using *refine2d* (EMAN software) based on 13,546 particles of hole-hole homodimer and 4,961 particles of NISTmAb.

### Overall structure of X-shaped particles in IgG homodimer and half-IgG by 2D mapping

Although individual images or class averages provide only 2D information of the particles and are insufficient to determine their 3D structures, raw images and class averages with high contrast could still provide important clues about the 3D orientation or even the overall structure of the particles. Here, we tried to determine the overall structure of the X-shaped particles in IgG homodimer by flexibly superposing an IgG model (the crystal structure of a human IgGl, PDB entry 1HZH^41^) onto images or class averages via the following protocol. First, the missing residues in the crystal structure were recovered using UCSF Chimera software. Next, the two Fab domains and the two heavy chain Fc regions in the crystal structure were separately rigid-body translated/rotated to obtain the best-fit for the corresponding domains obtained from images or class averages. To distinguish Fab domains from Fc regions obtained from the images and averages, the following criteria were used: i) two Fab domains are similar in size and shape; ii) the two halves of the Fc are both distinguishably smaller than an Fab domain. The above protocol was also used for determining the overall structure of half-IgG.

### Conformations of IgG homodimer and half-IgG

The resolution (approximately 12–15 Å) of the IPET 3D reconstructions was insufficient to determine the high-resolution atomic structure of each individual IgG. However, the reconstructions were sufficient to determine the overall domain orientations and positions and reveal the structural heterogeneity and dynamics of the homodimer with mutations in the Fc region. We chose the crystal structure of human IgG (PDB entry 1HZH^41^) as a model to reflect the IgG structural dynamics and flexibly docked it into the reconstruction of homodimer and half-IgG by using the following protocol. After the missing residues in the crystal structure were recovered by using Chimera, the Fab domains and heavy chain Fc regions were truncated from the crystal structure and separately rigid-body docked into the density map envelope using Chimera. Finally, the positions and orientations of the docked domains were used as guides for performing targeted molecular dynamics (TMD) simulations.

To distinguish Fab domains from the heavy chain Fc region and determine their overall location and orientation in the 3D density maps, the following criteria were used in addition to these in the previous section: the distance between the Fab domain and corresponding heavy chain Fc region should be allowed by the length of central loop.

By using the best-fit positions as the target positions, we drove the model to correlate with the density map by using a TMD simulation technique. The dragging and moving forces were pre-calculated and applied to all backbone atoms in each domain of the crystal structure to gradually steer the domains toward their corresponding best-fit positions and orientations. During this process, the domain structure and the chemical structure, including disulfide bonds, were constrained as the original crystal structure, whereas the loop regions were left flexible, allowing conformational changes to occur. As a result, the newly modeled structure of the IgG had the same domain structure as in the crystal structure, but had different relative domain positions and orientations.

The TMD simulation was performed using the NAMD2^101^ software and CHARMM27^102^ force fields. A cut-off distance for van der Waals interactions was set to 12 Å. The whole system was heated from 0 K to 310 K over a 62-ps simulation using weakly coupled Langevin dynamics, and the temperature was then maintained at 310 K. The pressure was maintained at 1 atm using a Langevin piston Nose-Hoover barostat (with a piston period of 100 fs and a decay time of 50 fs). It took 300 k steps to complete the conformational changes, and the simulation length was 0.6 ns.

### ET data acquisition and image pre-processing

The TEM holder was tilted at angles ranging from −45° to +45° in 1.5° increments, and imaging was controlled using Gatan tomography software (Zeiss Libra 120 Plus TEM). The TEM was operated at 120 kV high tension with a 20-eV energy filter. The tilt series was acquired under low defocus conditions (<1 μm) using a Gatan UltraScan 4 K X 4 K CCD under a magnification of 80 kx (each pixel of the micrographs corresponds to 1.48 Å in specimens). The total electron doses were 2,800–6,600 e^−^Å^−2^. The micrographs were initially aligned using the IMOD^103^ software package. The CTF was then corrected using TomoCTF^104^. The tilt series of the particles in square windows of 256 × 256 pixels (~38 nm) were semi-automatically tracked, windowed using IPET software^39^, and finally binned by 2 to reduce computation time in the subsequent reconstruction.

### IPET 3D reconstruction

In the IPET reconstruction process^39^, a tilt series of CTF-corrected images (~38 nm) containing a single IgG homodimer or half-IgG particle was directly back-projected into an *ab initi*o 3D density map as an initial model based on the corresponding goniometer tilt angles of images. The refinement was started using this initial model to align each tilt image via translational alignment to the projections of the initial model. During refinement, automatically generated low-pass filters, a circular-shaped mask with a Gaussian boundary, and particle-shaped masks with a Gaussian boundary were sequentially applied to the tilt images and references to increase the alignment accuracy^39^. The resolution was defined based on Fourier shell correction (FSC) calculation, in which the aligned images were split into two halves based on an odd- or even-numbered index to generate two 3D reconstructions for computing their FSC curve against the spatial frequency shells in Fourier space. The frequency at which the FSC curve first fell to a value of 0.5 was used to represent the resolution of the IPET 3D density map. All IPET density maps presented in the figures were low-pass filtered to 20 Å.

Notably, a tilt angle range of ±45° was used for IPET 3D reconstruction, which could lead to missing wedge artifacts, such as elongation, blurring, and distracting caustics^105^. However, the effects of the missing wedge can be reduced via computational algorithms as reported by numerous groups^105–108^. Agard and Stroud reported a computational approach to fill the missing data in 2D electron crystallography^109^. Recently, Kovacik, *et* *al*. used a simple Fourier angular filter^106^, Ruotsalainen *et* *al*. developed a statistical reconstruction method^110^, Sun *et* *al*. reported an iterative compressed-sensing^107^, and Miao *et* *al*. proposed a generalized Fourier iterative reconstruction algorithm (GENFIRE) to reduce the missing wedge artifact to achieve a 3D structure with more isotropic resolution^108^. In our IPET 3D reconstruction, we used our real-Fourier space iteration algorithm to fill the missing wedge^51,52,57,58^. The related methodology paper is in preparation.

### Statistical analysis of IgG homodimer dynamics

To quantify the conformational dynamics of the IgG homodimers, all 64 IgG homodimer conformations were aligned based on their C_H_2 domains using VMD software^111^. The distribution of distance and angle between Fab domains, distance between C_H_3 domains and angle between C_H_2 domains were then measured to study the fluctuations and dynamic features of the IgG homodimer. To measure distance, we first calculated the mass center of each Fab and C_H_3 domain and then measured the distance between the mass centers of the Fab domains (Fig. 5I), and between the mass centers of the C_H_3 domains (Fig. 5G). To measure angles, we first introduced vectors to determine the orientation of each Fab domain and each C_H_2 domain (Fig. 5H,J), and then measured angles between the vectors. These vectors were defined by using backbone atoms at the two far ends of Fab domains and C_H_2 domains.

The measured distances and angles from 48 X-shaped IgG homodimers were plotted onto histograms and fitted with polynomial or Gaussian curves using MATLAB. These histograms represented the conformational space of the IgG homodimer and indicated the extent of the structural dynamics of the homodimer.

### Construction of a homology model for IgG homodimer Fc domain

To study contacts between C_H_3 domains after hole mutations, a homology model for the Fc domain of the hole-hole homodimer was constructed. First, the two halves of the Fc region with hole mutations were derived from the crystal structure of homodimeric hole Fc fragment (PDB entry 4NQT^94^). The two halves were then separately superposed onto the corresponding halves of Fc region in the typical IgG using Chimera.

### Data deposition

The TEM 3D density maps of 64 IgG homodimers and 16 half-IgGs are available from the EM data bank as EMDB IDs 7353–7368, 7369–7417 and 7418–7433.

## Supplementary information

Supporting information

Supporting video
