# Supplementary material for: Single-Molecule 3D Images of “Hole-Hole” IgG1 Homodimers by Individual-Particle Electron Tomography
Source: Sci Rep. 2019 Jun 20;9:8864. doi: 10.1038/s41598-019-44978-7 (PMC6586654; doi:10.1038/s41598-019-44978-7)
Supplement: Supplementary file 1 — Supporting information [file 41598_2019_44978_MOESM1_ESM.pdf]

Supplementary Information

for

Single-Molecule 3D Images of "Hole-Hole" IgG1 Homodimers by Individual-Particle Electron Tomography

Dongsheng Lei,[1] Jianfang Liu,[1] Hongbin Liu,[2] Thomas E. Cleveland,[3] John P. Marino,[3] Ming Lei,[2,] *Gang Ren[1,]*

[1] The Molecular Foundry, Lawrence Berkeley National Laboratory, Berkeley, CA 94720, USA
[2] Genentech Inc, South San Francisco, CA 94080, USA
[3] Institute for Bioscience and Biotechnology Research, National Institute of Standards and Technology and the University of Maryland, Rockville, MD 20850, USA

* Correspondence should be addressed to: G. R. (gren@lbl.gov) or M.L. (lei.ming@gene.com)

Supplementary Table 1. The parameters used for IPET 3D reconstructions

#	EMDB#[1]	Shape	Dose/img.[2] ($e^-/Å^2$)	Dose/set[3] ($e^-/Å^2$)	Acq. angle range[4]	Total img.[5]	Reconst. angle range[6]	Cont.[7]	Resol.[8] (Å)	D_{CH3} (Å)	ϑ_{CH2} (˚)	D_{Fab} (Å)	ϑ_{Fab} (˚)	Fig. [9]
1	EMD-7353	Y-shape 01	70.31	4570.47	-48˚ to +48˚	65	-45˚ to 45˚	0.442	13.2	-	-	-	-	S2
2	EMD-7354	Y-shape 02	43.24	2983.37	-51˚ to +51˚	69	-45˚ to 45˚	0.239	13.6	-	-	-	-	S3
3	EMD-7355	Y-shape 03	99.42	6461.98	-48˚ to +48˚	65	-45˚ to 45˚	0.343	12.5	-	-	-	-	S4
4	EMD-7356	Y-shape 04	100.03	6501.88	-48˚ to +48˚	65	-45˚ to 45˚	0.249	12.8	-	-	-	-	S5
5	EMD-7357	Y-shape 05	98.94	6431.26	-48˚ to +48˚	65	-45˚ to 45˚	0.324	12.7	-	-	-	-	S6
6	EMD-7358	Y-shape 06	71.24	4630.30	-48˚ to +48˚	65	-45˚ to 45˚	0.362	14.1	-	-	-	-	S7
7	EMD-7359	Y-shape 07	100.13	6508.28	-48˚ to +48˚	65	-45˚ to 45˚	0.260	12.8	-	-	-	-	S8
8	EMD-7360	Y-shape 08	70.09	4556.14	-48˚ to +48˚	65	-45˚ to 45˚	0.265	12.9	-	-	-	-	S9
9	EMD-7361	Y-shape 09	70.57	4586.78	-48˚ to +48˚	65	-45˚ to 45˚	0.241	14.1	-	-	-	-	S10
10	EMD-7362	Y-shape 10	70.10	4556.44	-48˚ to +48˚	65	-45˚ to 45˚	0.203	13.5	-	-	-	-	S11
11	EMD-7363	Y-shape 11	40.72	2809.94	-51˚ to +51˚	69	-45˚ to 45˚	0.335	13.5	-	-	-	-	-
12	EMD-7364	Y-shape 12	43.73	3017.69	-51˚ to +51˚	69	-45˚ to 45˚	0.257	13.3	-	-	-	-	-
13	EMD-7365	Y-shape 13	41.79	2883.78	-51˚ to +51˚	69	-45˚ to 45˚	0.419	13.3	-	-	-	-	-
14	EMD-7366	Y-shape 14	41.36	2854.06	-51˚ to +51˚	69	-45˚ to 45˚	0.251	13.2	-	-	-	-	-
15	EMD-7367	Y-shape 15	70.30	4569.18	-48˚ to +48˚	65	-45˚ to 45˚	0.208	12.3	-	-	-	-	-
16	EMD-7368	Y-shape 16	97.65	6347.29	-48˚ to +48˚	65	-45˚ to 45˚	0.228	12.9	-	-	-	-	-
17	EMD-7369	X-shape 01	70.75	4598.56	-48˚ to +48˚	65	-45˚ to 45˚	0.306	13.7	106.4	77.3	79.3	125.4	S12
18	EMD-7370	X-shape 02	42.66	2943.60	-51˚ to +51˚	69	-45˚ to 45˚	0.288	13.2	87.8	173.2	88.8	119.2	S13
19	EMD-7371	X-shape 03	99.26	6451.61	-48˚ to +48˚	65	-45˚ to 45˚	0.289	12.5	96.7	149.9	57.4	40.4	S14
20	EMD-7372	X-shape 04	71.10	4621.51	-48˚ to +48˚	65	-45˚ to 45˚	0.394	13.9	80.1	81.8	74.0	73.8	S15
21	EMD-7373	X-shape 05	43.17	2978.80	-51˚ to +51˚	69	-45˚ to 45˚	0.270	13.6	88.2	44.5	77.9	137.7	S16
22	EMD-7374	X-shape 06	39.99	2759.35	-51˚ to +51˚	69	-45˚ to 45˚	0.341	13.4	97.9	138.1	80.9	109.4	S17
23	EMD-7375	X-shape 07	100.03	6501.94	-48˚ to +48˚	65	-45˚ to 45˚	0.387	12.8	94.4	137.3	66.8	75.0	S18
24	EMD-7376	X-shape 08	70.34	4571.86	-48˚ to +48˚	65	-45˚ to 45˚	0.358	12.6	39.3	80.0	72.8	107.0	S19
25	EMD-7377	X-shape 09	70.07	4554.84	-48˚ to +48˚	65	-45˚ to 45˚	0.332	12.7	106.9	120.6	87.2	137.8	S20
26	EMD-7378	X-shape 10	71.03	4616.89	-48˚ to +48˚	65	-45˚ to 45˚	0.392	14.1	100.6	136.2	91.4	69.3	S21
27	EMD-7379	X-shape 11	70.50	4582.44	-48˚ to +48˚	65	-45˚ to 45˚	0.326	12.8	92.1	15.6	92.7	114.4	S22
28	EMD-7380	X-shape 12	70.31	4570.41	-48˚ to +48˚	65	-45˚ to 45˚	0.346	11.9	87.2	115.4	74.2	122.9	S23
29	EMD-7381	X-shape 13	41.74	2880.14	-51˚ to +51˚	69	-45˚ to 45˚	0.394	13.5	99.2	122.5	90.6	120.6	S24
30	EMD-7382	X-shape 14	40.76	2812.50	-51˚ to +51˚	69	-45˚ to 45˚	0.325	14.0	53.8	22.9	72.9	56.2	S25
31	EMD-7383	X-shape 15	99.09	6440.60	-48˚ to +48˚	65	-45˚ to 45˚	0.299	12.5	47.1	27.6	87.7	112.8	S26
32	EMD-7384	X-shape 16	95.16	6185.12	-48˚ to +48˚	65	-45˚ to 45˚	0.383	13.5	88.5	58.4	82.6	141.3	S27
33	EMD-7385	X-shape 17	100.38	6524.42	-48˚ to +48˚	65	-45˚ to 45˚	0.370	12.3	89.6	137.1	84.1	85.0	S28
34	EMD-7386	X-shape 18	71.09	4620.67	-48˚ to +48˚	65	-45˚ to 45˚	0.263	13.7	95.8	52.6	97.4	143.1	S29
35	EMD-7387	X-shape	44.02	3037.25	-51˚ to +51˚	69	-45˚ to 45˚	0.262	12.9	106.7	126.7	98.4	150.1	S30

		19												
36	EMD-7388	X-shape 20	70.48	4581.01	-48˚ to +48˚	65	-45˚ to 45˚	0.387	12.0	81.0	36.3	86.3	141.1	S31
37	EMD-7389	X-shape 21	70.14	4558.92	-48˚ to +48˚	65	-45˚ to 45˚	0.324	12.2	85.7	48.7	106.8	159.9	S32
38	EMD-7390	X-shape 22	70.15	4559.58	-48˚ to +48˚	65	-45˚ to 45˚	0.401	12.3	76.1	69.9	108.0	110.6	S33
39	EMD-7391	X-shape 23	70.63	4591.20	-48˚ to +48˚	65	-45˚ to 45˚	0.275	13.6	73.1	16.4	91.2	125.7	S34
40	EMD-7392	X-shape 24	99.79	6486.67	-48˚ to +48˚	65	-45˚ to 45˚	0.372	12.2	101.8	164.4	59.4	80.5	S35
41	EMD-7393	X-shape 25	70.44	4578.89	-48˚ to +48˚	65	-45˚ to 45˚	0.364	12.3	97.1	92.2	77.3	71.8	-
42	EMD-7394	X-shape 26	70.68	4593.88	-48˚ to +48˚	65	-45˚ to 45˚	0.472	12.4	94.5	162.3	111.4	171.5	-
43	EMD-7395	X-shape 27	44.29	3056.08	-51˚ to +51˚	69	-45˚ to 45˚	0.304	13.6	81.3	25.9	84.6	118.8	-
44	EMD-7396	X-shape 28	43.16	2978.33	-51˚ to +51˚	69	-45˚ to 45˚	0.334	13.1	89.1	85.7	91.8	139.6	-
45	EMD-7397	X-shape 29	100.25	6516.05	-48˚ to +48˚	65	-45˚ to 45˚	0.380	12.6	50.0	49.9	90.1	74.7	-
46	EMD-7398	X-shape 30	70.44	4578.57	-48˚ to +48˚	65	-45˚ to 45˚	0.335	12.6	119.9	77.3	92.5	122.5	-
47	EMD-7399	X-shape 31	70.57	4587.12	-48˚ to +48˚	65	-45˚ to 45˚	0.304	12.2	85.5	53.2	78.9	96.0	-
48	EMD-7400	X-shape 32	40.53	2796.79	-51˚ to +51˚	69	-45˚ to 45˚	0.411	13.8	114.2	128.0	86.2	116.0	-
49	EMD-7401	X-shape 33	70.63	4590.67	-48˚ to +48˚	65	-45˚ to 45˚	0.392	13.2	91.5	59.7	86.9	113.5	-
50	EMD-7402	X-shape 34	70.23	4564.90	-48˚ to +48˚	65	-45˚ to 45˚	0.333	11.9	66.8	37.3	80.1	80.3	-
51	EMD-7404	X-shape 35	43.85	3025.72	-51˚ to +51˚	69	-45˚ to 45˚	0.229	13.6	37.0	86.5	98.7	145.3	-
52	EMD-7405	X-shape 36	70.63	4590.93	-48˚ to +48˚	65	-45˚ to 45˚	0.372	12.1	61.6	35.7	85.3	136.4	-
53	EMD-7406	X-shape 37	70.70	4595.56	-48˚ to +48˚	65	-45˚ to 45˚	0.248	12.1	93.9	82.6	90.3	123.6	-
54	EMD-7407	X-shape 38	70.50	4582.72	-48˚ to +48˚	65	-45˚ to 45˚	0.303	13.9	100.3	55.0	89.1	146.4	-
55	EMD-7408	X-shape 39	70.61	4589.47	-48˚ to +48˚	65	-45˚ to 45˚	0.296	13.6	85.5	12.6	62.1	15.7	-
56	EMD-7409	X-shape 40	70.90	4608.70	-48˚ to +48˚	65	-45˚ to 45˚	0.299	13.4	45.6	8.0	80.0	83.9	-
57	EMD-7410	X-shape 41	70.12	4557.54	-48˚ to +48˚	65	-45˚ to 45˚	0.275	12.5	95.4	117.4	73.2	113.5	-
58	EMD-7411	X-shape 42	70.87	4606.42	-48˚ to +48˚	65	-45˚ to 45˚	0.395	12.3	86.9	125.8	93.1	145.1	-
59	EMD-7412	X-shape 43	69.81	4537.84	-48˚ to +48˚	65	-45˚ to 45˚	0.228	12.6	76.4	41.4	76.6	79.2	-
60	EMD-7413	X-shape 44	69.72	4531.56	-48˚ to +48˚	65	-45˚ to 45˚	0.275	12.0	83.1	34.5	92.4	139.3	-
61	EMD-7414	X-shape 45	70.65	4592.31	-48˚ to +48˚	65	-45˚ to 45˚	0.330	12.2	65.1	76.0	76.8	52.6	-
62	EMD-7415	X-shape 46	71.13	4623.45	-48˚ to +48˚	65	-45˚ to 45˚	0.239	14.3	94.5	20.9	103.2	169.6	-
63	EMD-7416	X-shape 47	70.84	4604.39	-48˚ to +48˚	65	-45˚ to 45˚	0.309	12.6	84.9	99.1	96.9	139.1	-
64	EMD-7417	X-shape 48	69.96	4547.66	-48˚ to +48˚	65	-45˚ to 45˚	0.376	12.6	76.9	23.0	75.8	77.5	-
65	EMD-7418	i-shape 01	99.06	6438.89	-48˚ to +48˚	65	-45˚ to 45˚	0.444	12.8	-	-	-	-	S36
66	EMD-7419	i-shape 02	41.73	2879.62	-51˚ to +51˚	69	-45˚ to 45˚	0.356	13.3	-	-	-	-	S37
67	EMD-7420	i-shape 03	70.60	4588.70	-48˚ to +48˚	65	-45˚ to 45˚	0.579	13.6	-	-	-	-	S38
68	EMD-7421	i-shape 04	97.47	6335.45	-48˚ to +48˚	65	-45˚ to 45˚	0.465	13.0	-	-	-	-	S39
69	EMD-7422	i-shape 05	70.55	4585.67	-48˚ to +48˚	65	-45˚ to 45˚	0.472	12.3	-	-	-	-	S40
70	EMD-7423	i-shape 06	98.09	6375.59	-48˚ to +48˚	65	-45˚ to 45˚	0.397	12.8	-	-	-	-	S41
71	EMD-7424	i-shape 07	39.63	2734.57	-51˚ to +51˚	69	-45˚ to 45˚	0.564	13.2	-	-	-	-	S42

72	EMD-7425	i-shape 08	70.44	4578.43	-48° to +48°	65	-45° to 45°	0.279	12.8	-	-	-	-	S43
73	EMD-7426	i-shape 09	70.62	4590.57	-48° to +48°	65	-45° to 45°	0.339	12.6	-	-	-	-	S44
74	EMD-7427	i-shape 10	71.21	4628.39	-48° to +48°	65	-45° to 45°	0.284	14.1	-	-	-	-	S45
75	EMD-7428	i-shape 11	43.26	2985.20	-51° to +51°	69	-45° to 45°	0.333	13.4	-	-	-	-	-
76	EMD-7429	i-shape 12	40.13	2769.02	-51° to +51°	69	-45° to 45°	0.428	13.3	-	-	-	-	-
77	EMD-7430	i-shape 13	70.67	4593.68	-48° to +48°	65	-45° to 45°	0.434	12.7	-	-	-	-	-
78	EMD-7431	i-shape 14	70.32	4570.79	-48° to +48°	65	-45° to 45°	0.435	13.7	-	-	-	-	-
79	EMD-7432	i-shape 15	70.81	4602.44	-48° to +48°	65	-45° to 45°	0.305	13.4	-	-	-	-	-
80	EMD-7433	i-shape 16	71.27	4632.59	-48° to +48°	65	-45° to 45°	0.429	13.9	-	-	-	-	-
**#**	**EMDB#[1]**	**Shape**	**Dose/img.[2] (e^-/Å^2)**	**Dose/set[3] (e^-/Å^2)**	**Acq. angle range[4]**	**Total img.[5]**	**Reconst. angle range[6]**	**Cont.[7]**	**Resol.[8] (Å)**	***D_{CH3}* (Å)**	***ϑ_{CH2}* (°)**	***D_{Fab}* (Å)**	***ϑ_{Fab}* (°)**	**Fig. [9]**

TEM: Zeiss 120 stands for Zeiss Libra 120 Plus TEM;
CCD: UltraScan for Gatan UltraScan 4000 4Kx4K CCD
Angstrom per pixel: 2.96 Å
[1]EMDB Index: https://www.ebi.ac.uk/pdbe/emdb/
[2]Dose used for each CCD frame
[3]Dose used for whole tilt series
[4]Data acquisition angle range
[5]Total images in the tilt series
[6]Reconstruction angle range
[7]Contour used for display
[8]IPET 3D reconstruction resolution
[9]The process of IPET 3D reconstruction showed in Supplementary Figure

Supplementary Figures

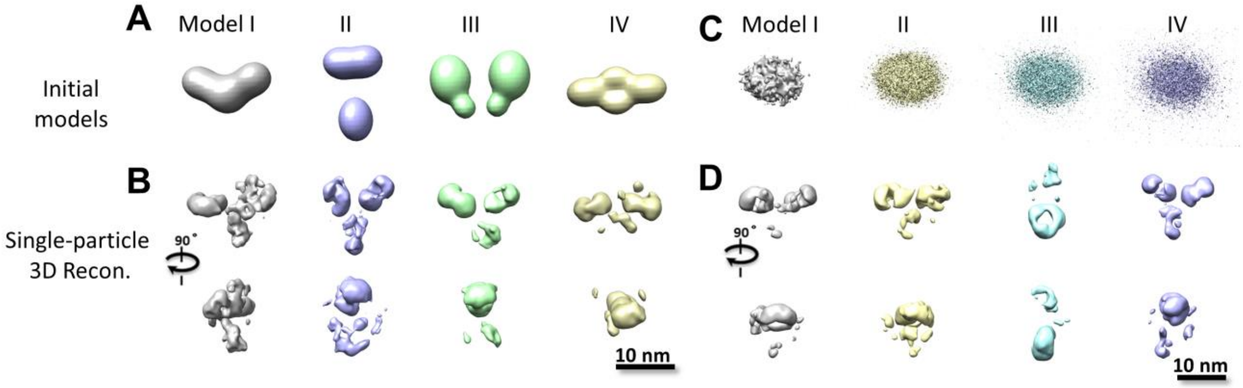

Supplementary Fig. 1 | Single-particle 3D reconstructions of hole-hole homodimer. Two sets of initial models were used to perform single particle reconstruction. **(A)** Four featureless blobs are used as the initial model for multi-model 3D refinement. **(B)** Final 3D reconstructions obtained based on corresponding initial models by using the multi-model 3D refinement algorithm. **(C)** Four single Gaussian blobs under different noise levels are used as the initial models for multi-model 3D refinement. **(D)** Final 3D reconstructions obtained based on corresponding initial models by using the multi-model 3D refinement algorithm. Scale bars=10 nm.

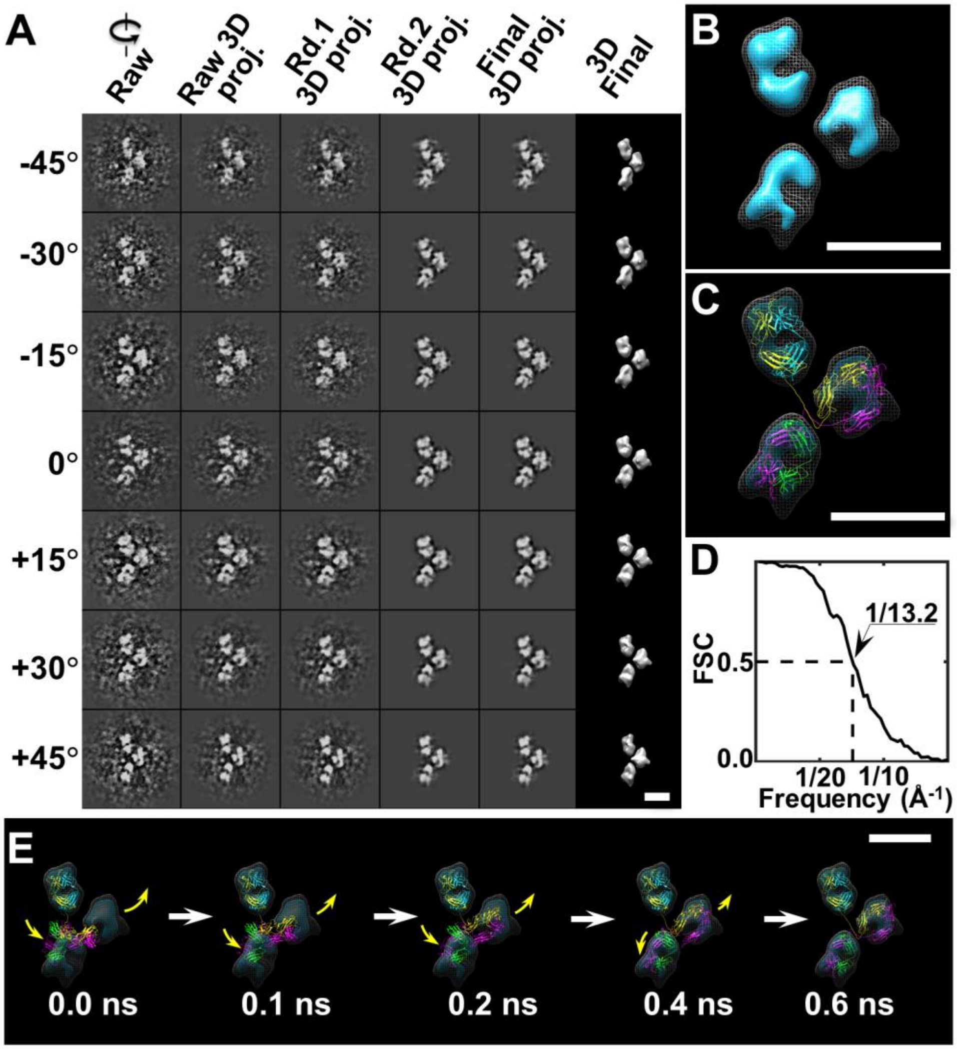

Supplementary Fig. 2 | 3D IPET reconstruction of the first Y-shaped IgG homodimer by IPET. (A) Seven representative tilt images of an individual Y-shaped particle are displayed in the first column from the left. Using IPET, the tilt images (after CTF correction) were gradually aligned to a common center for 3D reconstruction via iterative refinement. Projections of raw, intermediate and final 3D reconstructions at the corresponding tilt angles are displayed in the next five columns according to their corresponding tilt angles. **(B)** The final 3D density map. **(C)** The density map was flexibly docked with IgG crystal structure by using TMD simulation. **(D)** FSC analyses (from two density maps reconstructed from odd and even numbers of tilt images) showed that the resolution of the final 3D density map was ~13.2 Å. **(E)** Five snapshots illustrated the conformational changes of IgG model during TMD simulation. Scale bars=10 nm.

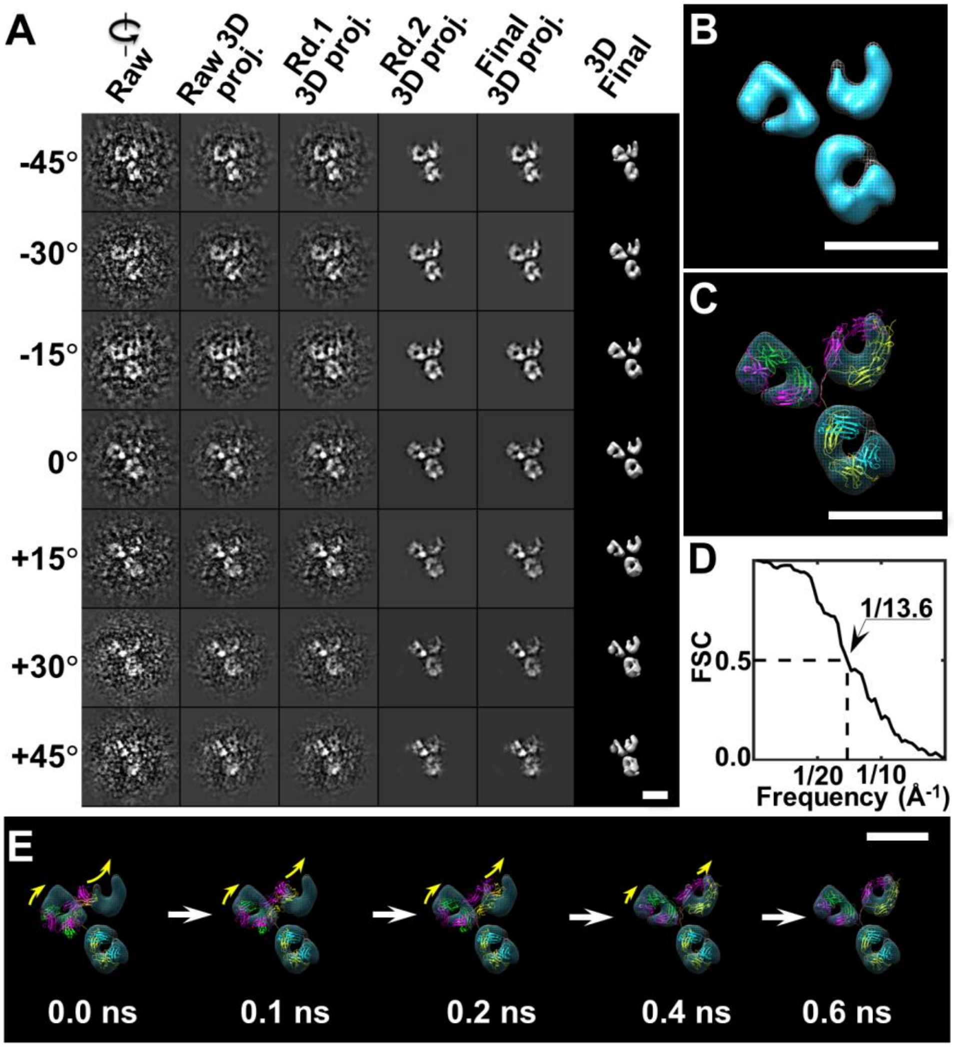

Supplementary Fig. 3 | 3D IPET reconstruction of the second Y-shaped IgG homodimer by IPET. (A) Seven representative tilt images of an individual Y-shaped particle are displayed in the first column from the left. Using IPET, the tilt images (after CTF correction) were gradually aligned to a common center for 3D reconstruction via iterative refinement. Projections of raw, intermediate and final 3D reconstructions at the corresponding tilt angles are displayed in the next five columns according to their corresponding tilt angles. **(B)** The final 3D density map. **(C)** The density map was flexibly docked with IgG crystal structure by using TMD simulation. **(D)** FSC analyses (from two density maps reconstructed from odd and even numbers of tilt images) showed that the resolution of the final 3D density map was ~13.6 Å. **(E)** Five snapshots illustrated the conformational changes of IgG model during TMD simulation. Scale bars=10 nm.

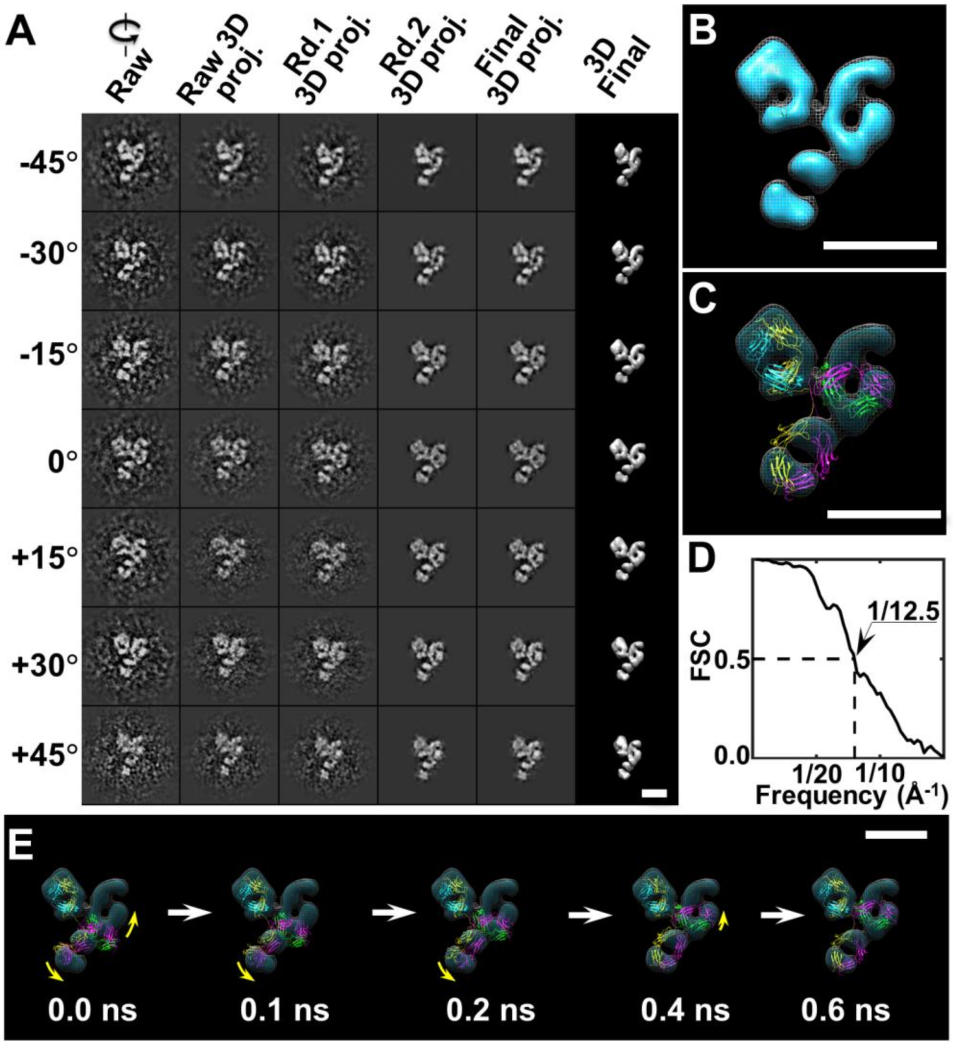

Supplementary Fig. 4 | 3D IPET reconstruction of the third Y-shaped IgG homodimer by IPET. (A) Seven representative tilt images of an individual Y-shaped particle are displayed in the first column from the left. Using IPET, the tilt images (after CTF correction) were gradually aligned to a common center for 3D reconstruction via iterative refinement. Projections of raw, intermediate and final 3D reconstructions at the corresponding tilt angles are displayed in the next five columns according to their corresponding tilt angles. **(B)** The final 3D density map. **(C)** The density map was flexibly docked with IgG crystal structure by using TMD simulation. **(D)** FSC analyses (from two density maps reconstructed from odd and even numbers of tilt images) showed that the resolution of the final 3D density map was ~12.5 Å. **(E)** Five snapshots illustrated the conformational changes of IgG model during TMD simulation. Scale bars=10 nm.

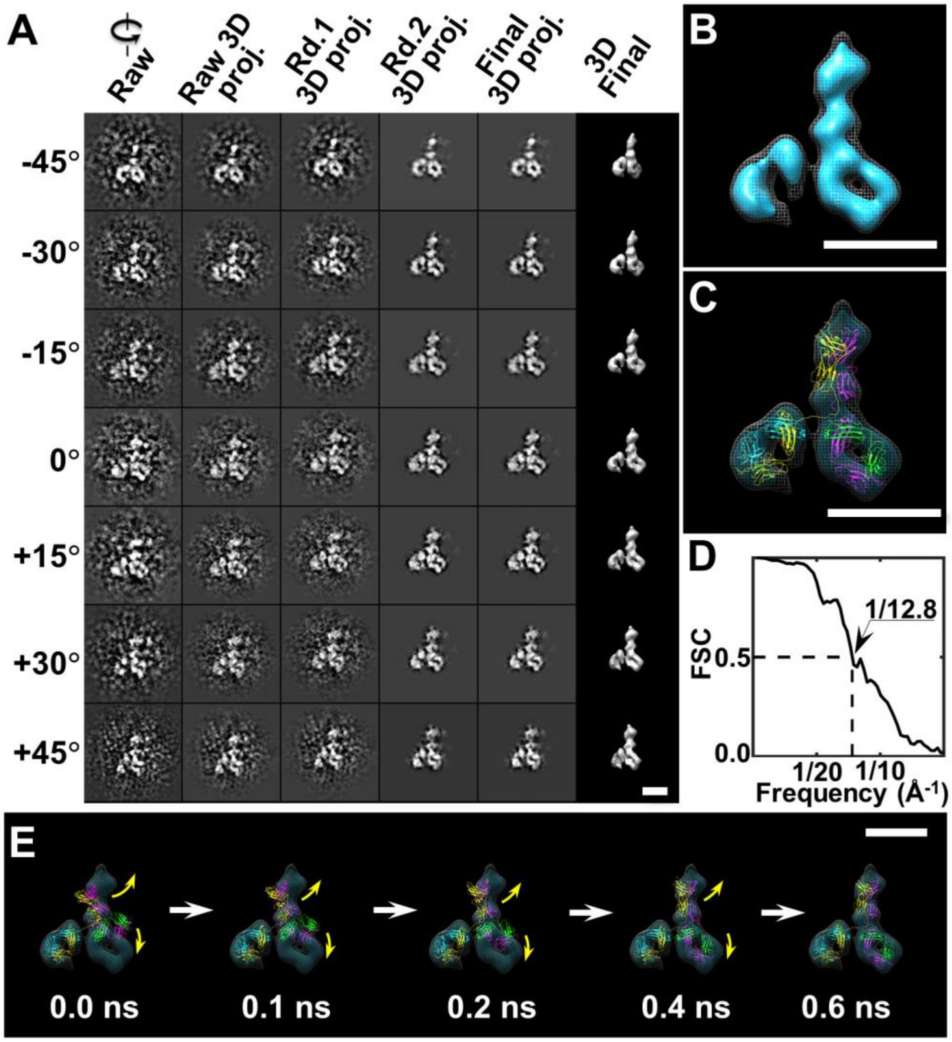

Supplementary Fig. 5 | 3D IPET reconstruction of the forth Y-shaped IgG homodimer by IPET. (A) Seven representative tilt images of an individual Y-shaped particle are displayed in the first column from the left. Using IPET, the tilt images (after CTF correction) were gradually aligned to a common center for 3D reconstruction via iterative refinement. Projections of raw, intermediate and final 3D reconstructions at the corresponding tilt angles are displayed in the next five columns according to their corresponding tilt angles. **(B)** The final 3D density map. **(C)** The density map was flexibly docked with IgG crystal structure by using TMD simulation. **(D)** FSC analyses (from two density maps reconstructed from odd and even numbers of tilt images) showed that the resolution of the final 3D density map was ~12.8 Å. **(E)** Five snapshots illustrated the conformational changes of IgG model during TMD simulation. Scale bars=10 nm.

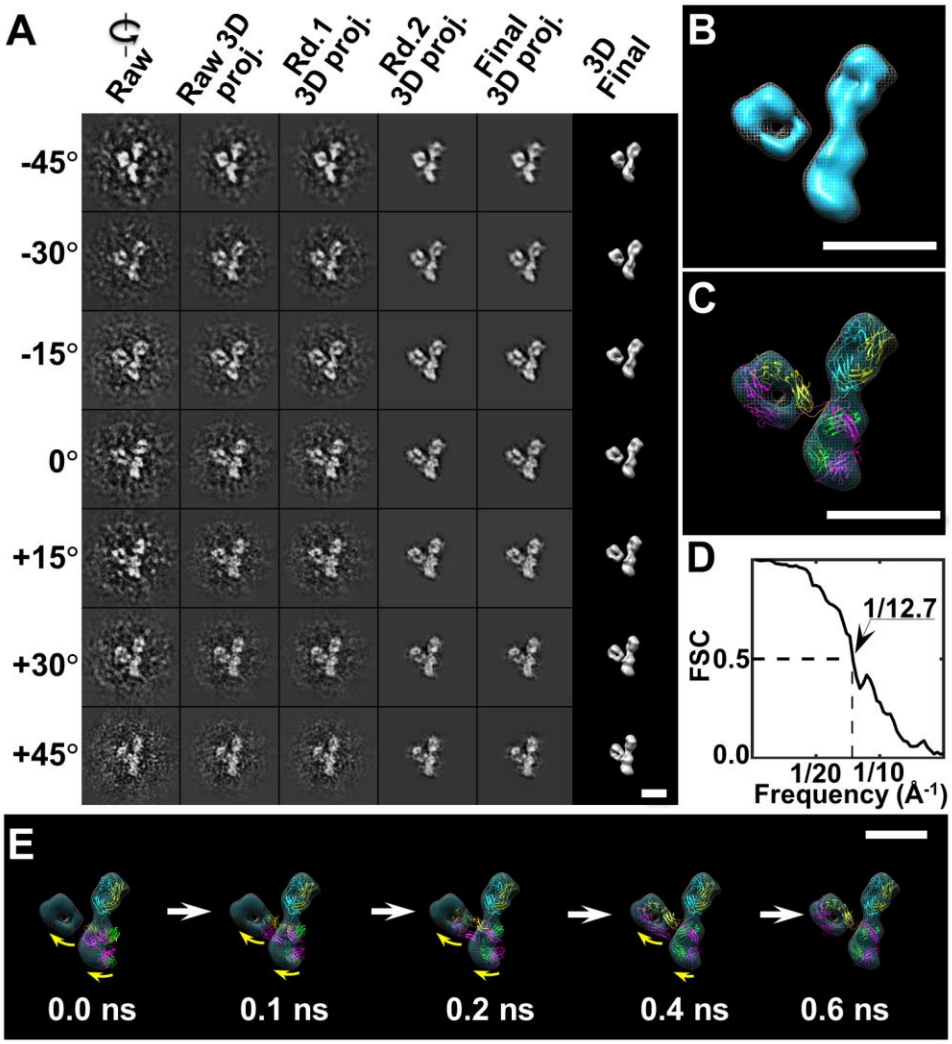

Supplementary Fig. 6 | 3D IPET reconstruction of the fifth Y-shaped IgG homodimer by IPET. (A) Seven representative tilt images of an individual Y-shaped particle are displayed in the first column from the left. Using IPET, the tilt images (after CTF correction) were gradually aligned to a common center for 3D reconstruction via iterative refinement. Projections of raw, intermediate and final 3D reconstructions at the corresponding tilt angles are displayed in the next five columns according to their corresponding tilt angles. **(B)** The final 3D density map. **(C)** The density map was flexibly docked with IgG crystal structure by using TMD simulation. **(D)** FSC analyses (from two density maps reconstructed from odd and even numbers of tilt images) showed that the resolution of the final 3D density map was ~12.7 Å. **(E)** Five snapshots illustrated the conformational changes of IgG model during TMD simulation. Scale bars=10 nm.

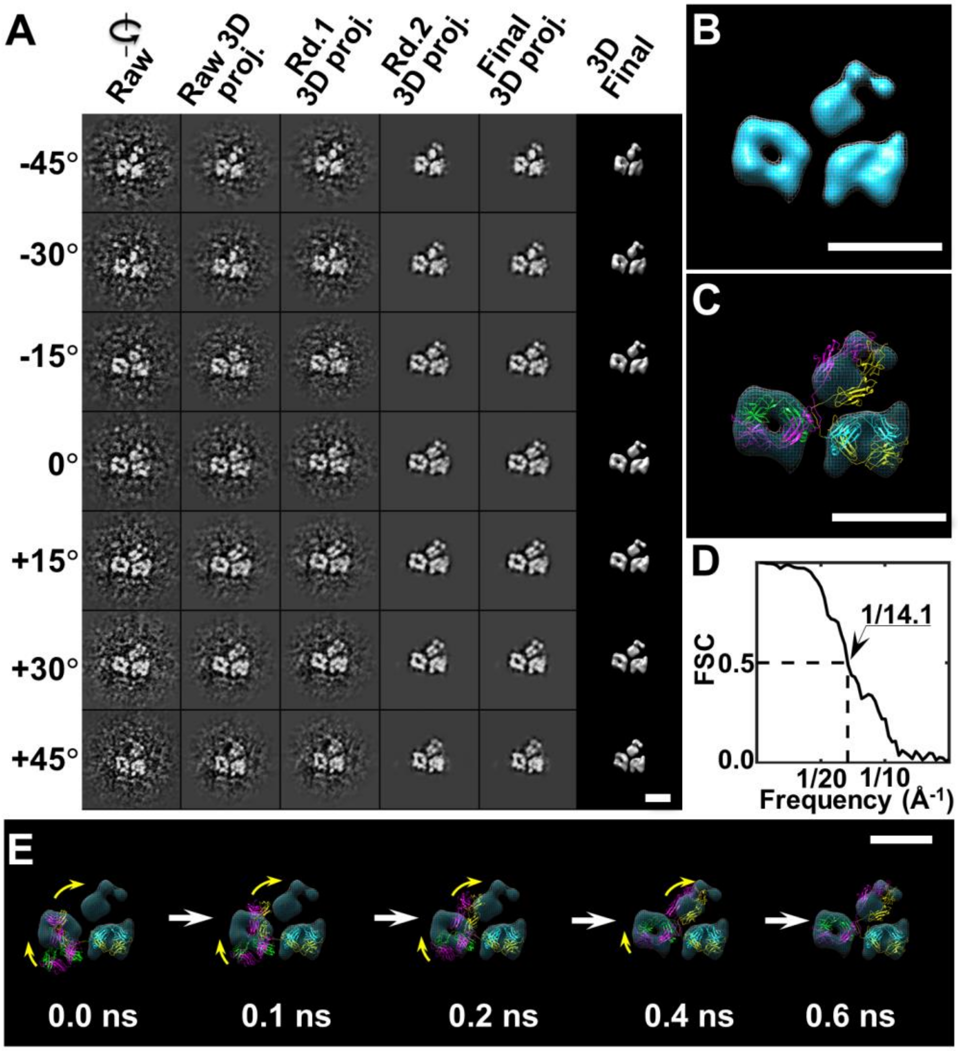

Supplementary Fig. 7 | 3D IPET reconstruction of the sixth Y-shaped IgG homodimer by IPET. (A) Seven representative tilt images of an individual Y-shaped particle are displayed in the first column from the left. Using IPET, the tilt images (after CTF correction) were gradually aligned to a common center for 3D reconstruction via iterative refinement. Projections of raw, intermediate and final 3D reconstructions at the corresponding tilt angles are displayed in the next five columns according to their corresponding tilt angles. **(B)** The final 3D density map. **(C)** The density map was flexibly docked with IgG crystal structure by using TMD simulation. **(D)** FSC analyses (from two density maps reconstructed from odd and even numbers of tilt images) showed that the resolution of the final 3D density map was ~14.1 Å. **(E)** Five snapshots illustrated the conformational changes of IgG model during TMD simulation. Scale bars=10 nm.

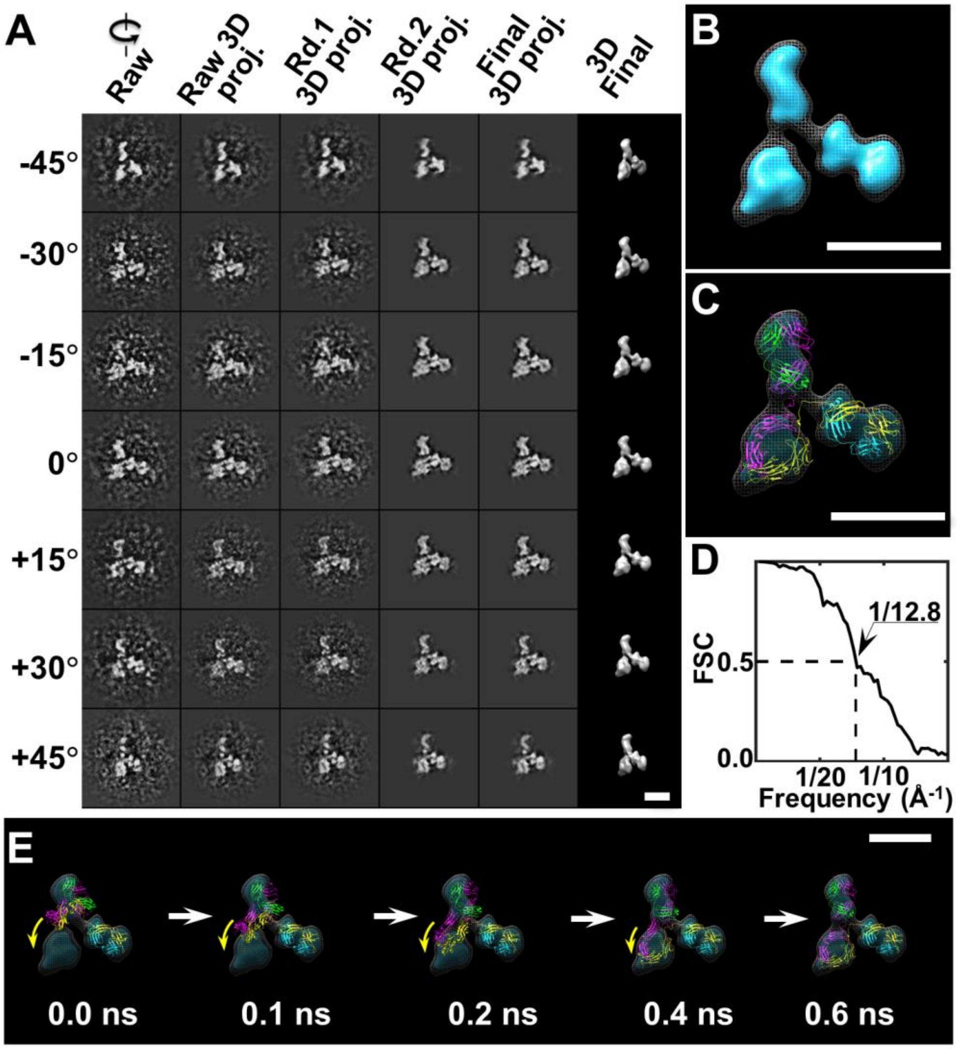

Supplementary Fig. 8 | 3D IPET reconstruction of the seventh Y-shaped IgG homodimer by IPET. **(A)** Seven representative tilt images of an individual Y-shaped particle are displayed in the first column from the left. Using IPET, the tilt images (after CTF correction) were gradually aligned to a common center for 3D reconstruction via iterative refinement. Projections of raw, intermediate and final 3D reconstructions at the corresponding tilt angles are displayed in the next five columns according to their corresponding tilt angles. **(B)** The final 3D density map. **(C)** The density map was flexibly docked with IgG crystal structure by using TMD simulation. **(D)** FSC analyses (from two density maps reconstructed from odd and even numbers of tilt images) showed that the resolution of the final 3D density map was ~12.8 Å. **(E)** Five snapshots illustrated the conformational changes of IgG model during TMD simulation. Scale bars=10 nm.

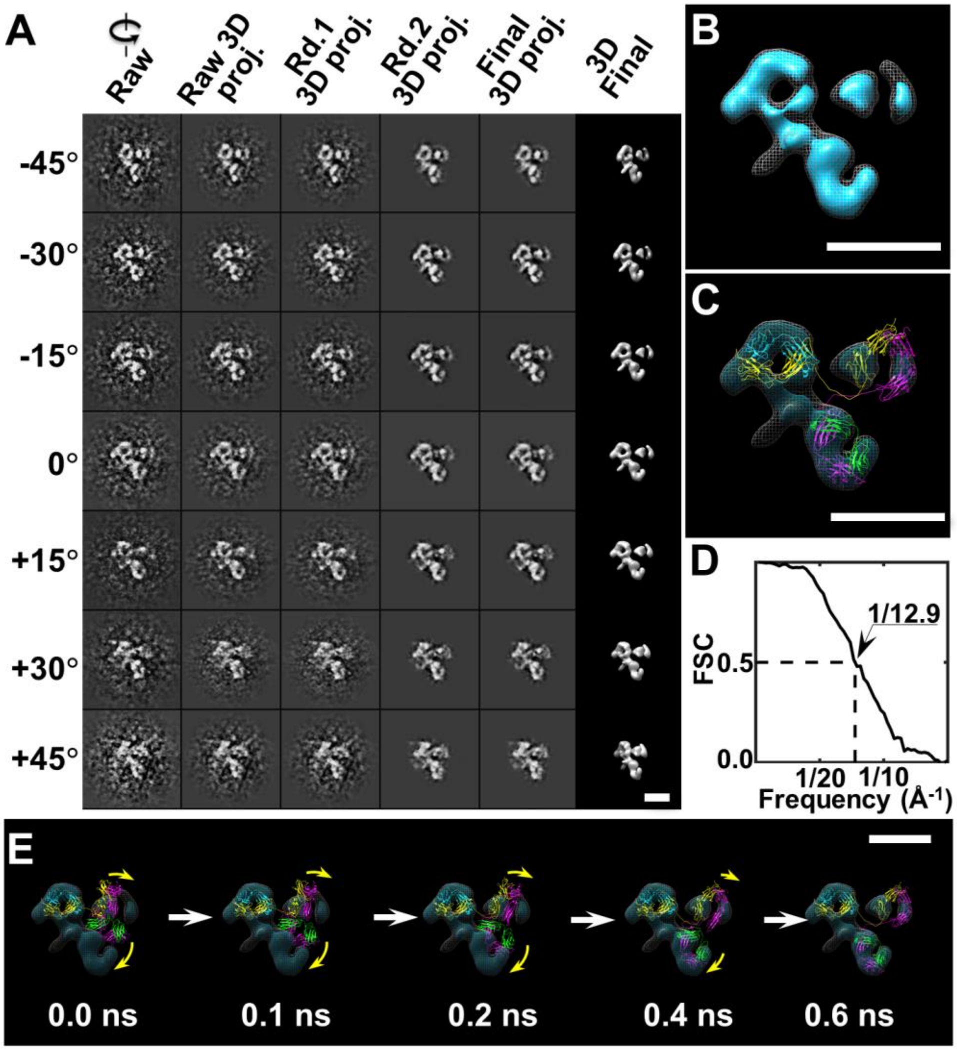

Supplementary Fig. 9 | 3D IPET reconstruction of the eighth Y-shaped IgG homodimer by IPET. (A) Seven representative tilt images of an individual Y-shaped particle are displayed in the first column from the left. Using IPET, the tilt images (after CTF correction) were gradually aligned to a common center for 3D reconstruction via iterative refinement. Projections of raw, intermediate and final 3D reconstructions at the corresponding tilt angles are displayed in the next five columns according to their corresponding tilt angles. **(B)** The final 3D density map. **(C)** The density map was flexibly docked with IgG crystal structure by using TMD simulation. **(D)** FSC analyses (from two density maps reconstructed from odd and even numbers of tilt images) showed that the resolution of the final 3D density map was ~12.9 Å. **(E)** Five snapshots illustrated the conformational changes of IgG model during TMD simulation. Scale bars=10 nm.

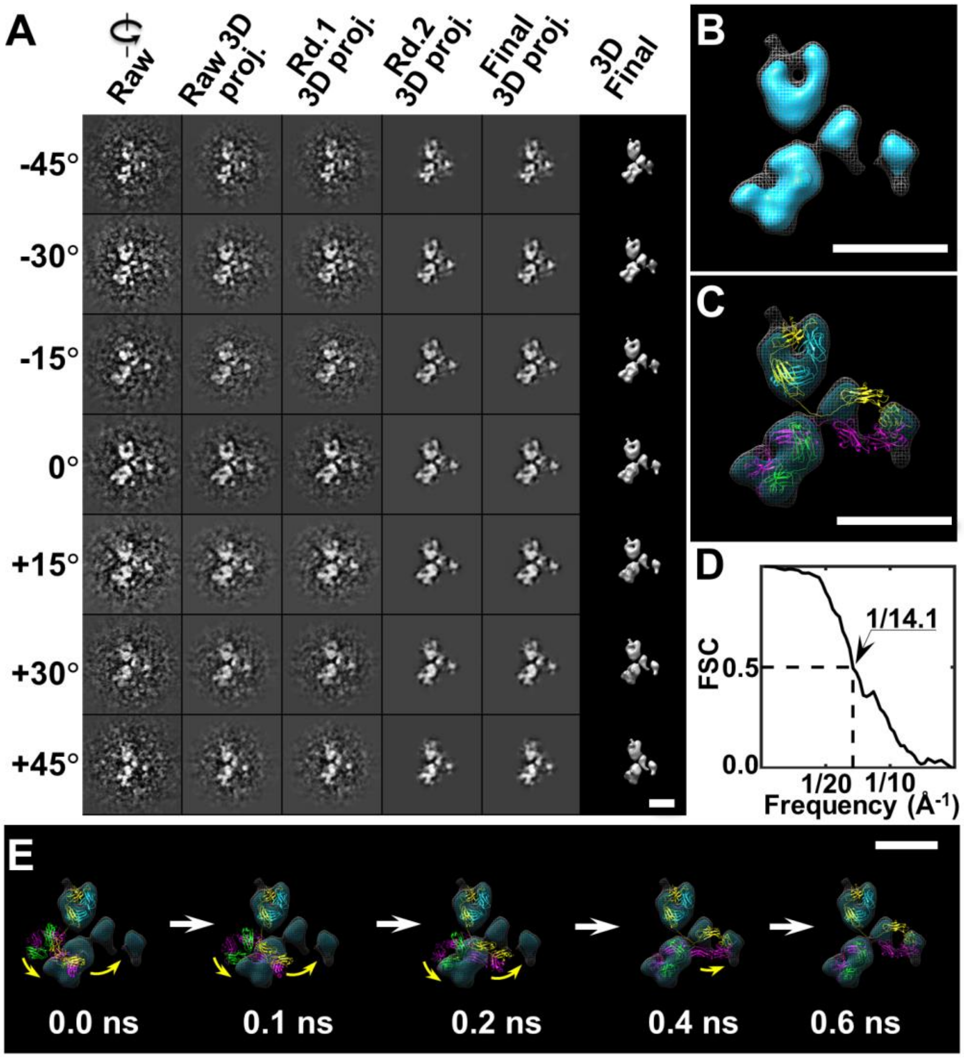

Supplementary Fig. 10 | 3D IPET reconstruction of the ninth Y-shaped IgG homodimer by IPET. (A) Seven representative tilt images of an individual Y-shaped particle are displayed in the first column from the left. Using IPET, the tilt images (after CTF correction) were gradually aligned to a common center for 3D reconstruction via iterative refinement. Projections of raw, intermediate and final 3D reconstructions at the corresponding tilt angles are displayed in the next five columns according to their corresponding tilt angles. **(B)** The final 3D density map. **(C)** The density map was flexibly docked with IgG crystal structure by using TMD simulation. **(D)** FSC analyses (from two density maps reconstructed from odd and even numbers of tilt images) showed that the resolution of the final 3D density map was ~14.1 Å. **(E)** Five snapshots illustrated the conformational changes of IgG model during TMD simulation. Scale bars=10 nm.

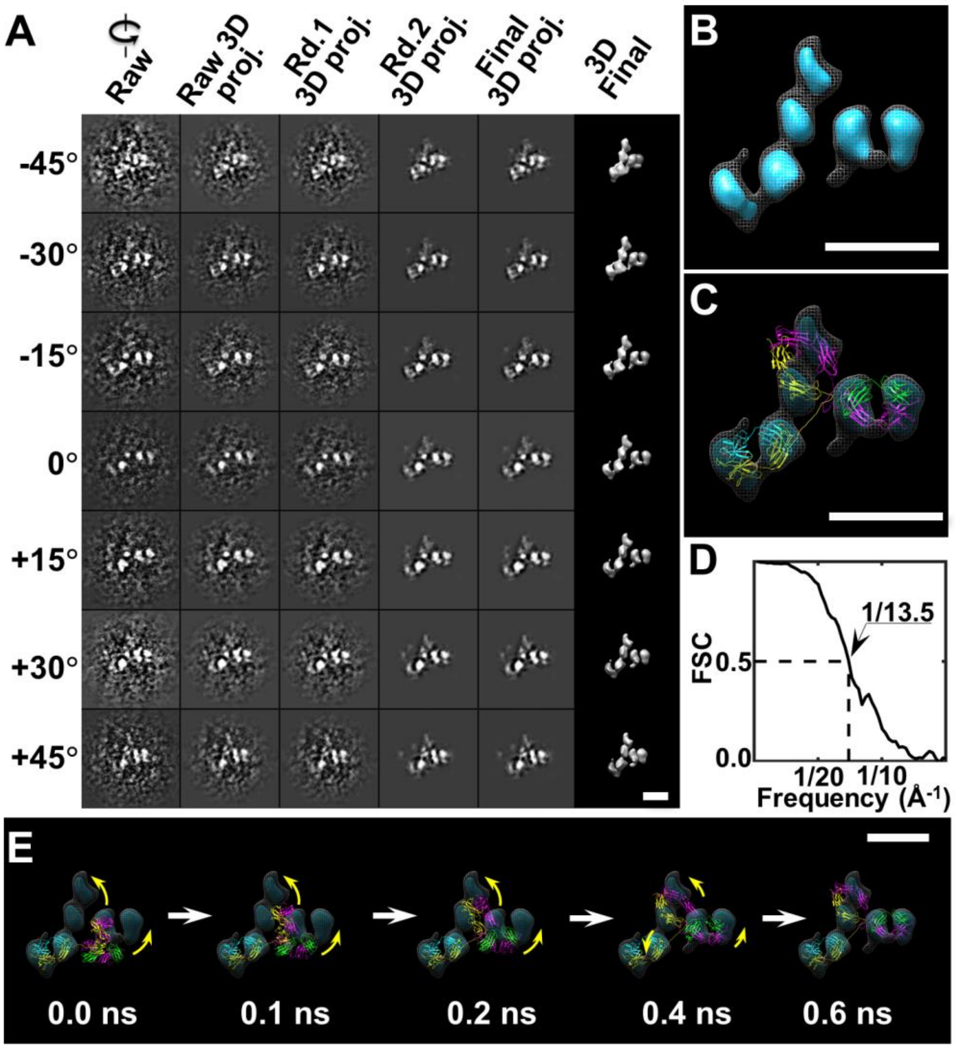

Supplementary Fig. 11 | 3D IPET reconstruction of the tenth Y-shaped IgG homodimer by IPET. **(A)** Seven representative tilt images of an individual Y-shaped particle are displayed in the first column from the left. Using IPET, the tilt images (after CTF correction) were gradually aligned to a common center for 3D reconstruction via iterative refinement. Projections of raw, intermediate and final 3D reconstructions at the corresponding tilt angles are displayed in the next five columns according to their corresponding tilt angles. **(B)** The final 3D density map. **(C)** The density map was flexibly docked with IgG crystal structure by using TMD simulation. **(D)** FSC analyses (from two density maps reconstructed from odd and even numbers of tilt images) showed that the resolution of the final 3D density map was ~13.5 Å. **(E)** Five snapshots illustrated the conformational changes of IgG model during TMD simulation. Scale bars=10 nm.

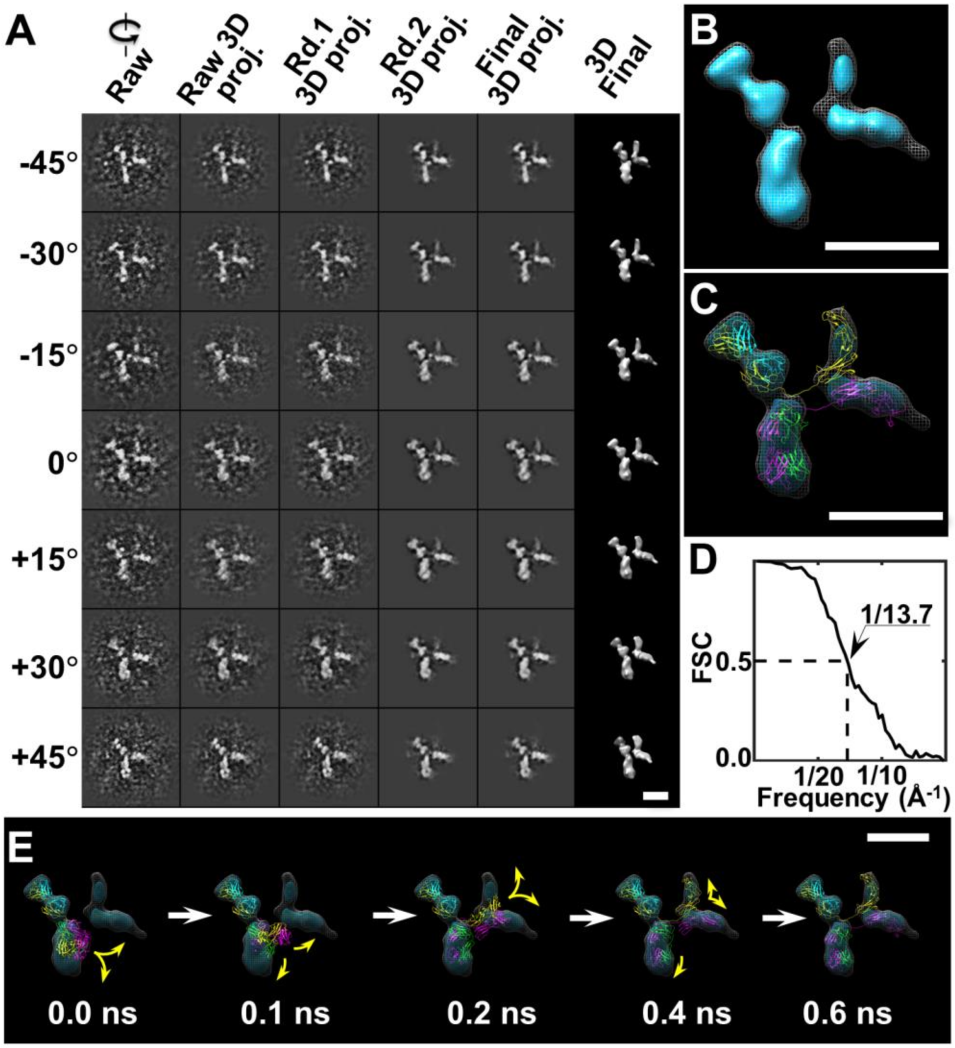

Supplementary Fig. 12 | 3D IPET reconstruction of the first X-shaped IgG homodimer by IPET. (A) Seven representative tilt images of an individual X-shaped particle are displayed in the first column from the left. Using IPET, the tilt images (after CTF correction) were gradually aligned to a common center for 3D reconstruction via iterative refinement. Projections of raw, intermediate and final 3D reconstructions at the corresponding tilt angles are displayed in the next five columns according to their corresponding tilt angles. **(B)** The final 3D density map. **(C)** The density map was flexibly docked with IgG crystal structure by using TMD simulation. **(D)** FSC analyses (from two density maps reconstructed from odd and even numbers of tilt images) showed that the resolution of the final 3D density map was ~13.7 Å. **(E)** Five snapshots illustrated the conformational changes of IgG model during TMD simulation. Scale bars=10 nm.

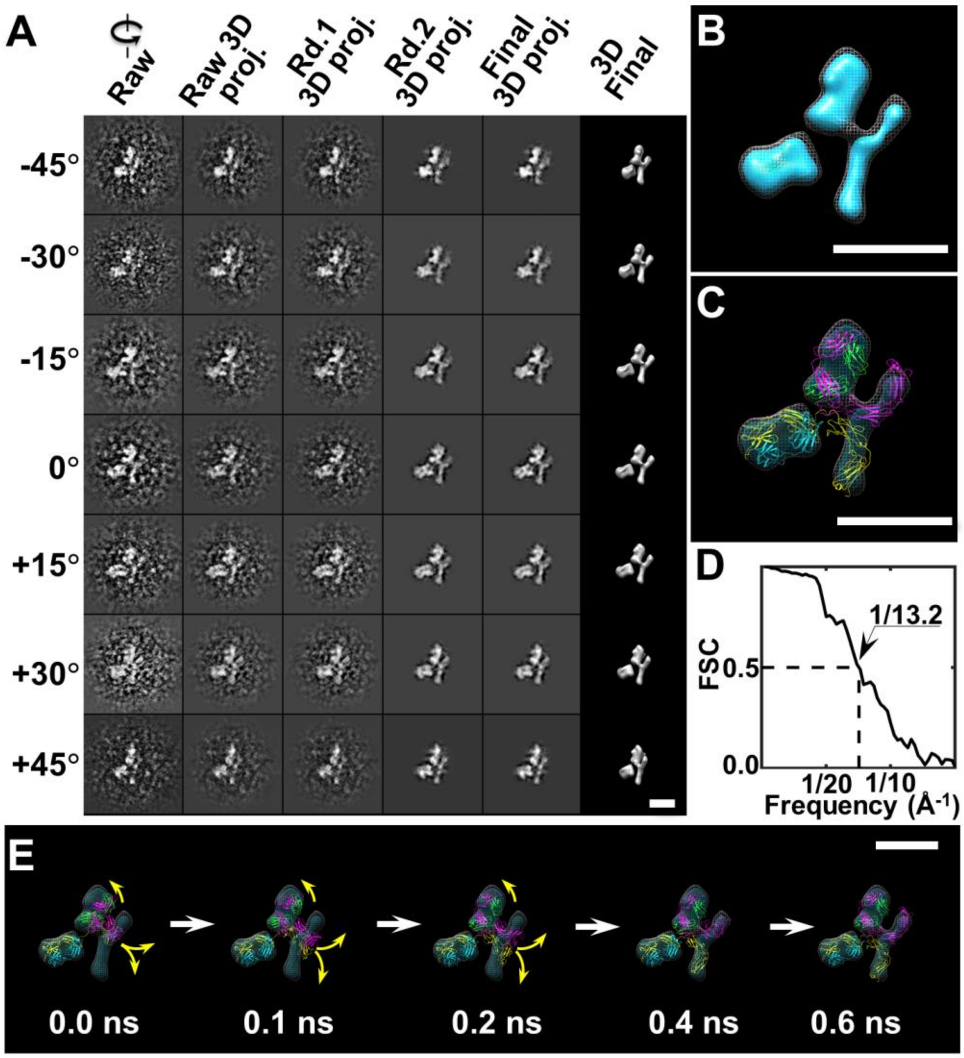

Supplementary Fig. 13 | 3D IPET reconstruction of the second X-shaped IgG homodimer by IPET. (A) Seven representative tilt images of an individual X-shaped particle are displayed in the first column from the left. Using IPET, the tilt images (after CTF correction) were gradually aligned to a common center for 3D reconstruction via iterative refinement. Projections of raw, intermediate and final 3D reconstructions at the corresponding tilt angles are displayed in the next five columns according to their corresponding tilt angles. **(B)** The final 3D density map. **(C)** The density map was flexibly docked with IgG crystal structure by using TMD simulation. **(D)** FSC analyses (from two density maps reconstructed from odd and even numbers of tilt images) showed that the resolution of the final 3D density map was ~13.2 Å. **(E)** Five snapshots illustrated the conformational changes of IgG model during TMD simulation. Scale bars=10 nm.

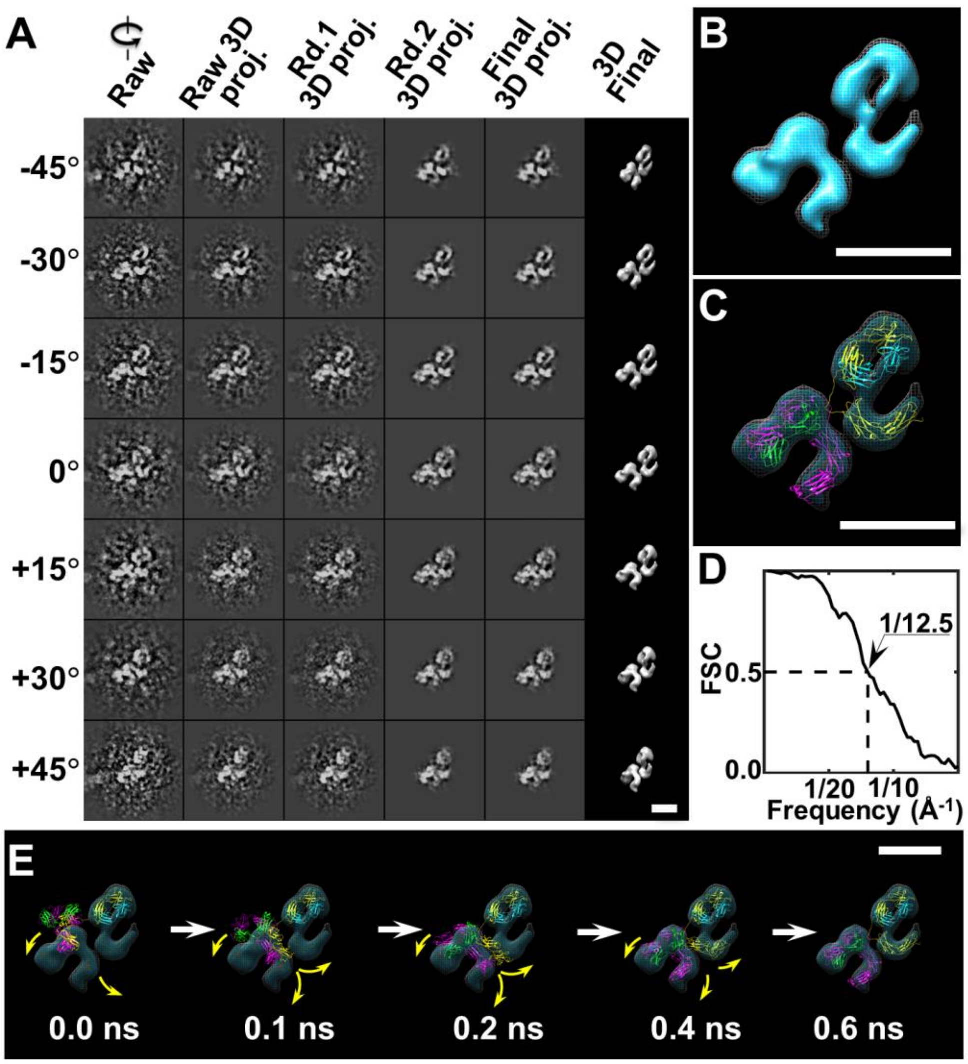

Supplementary Fig. 14 | 3D IPET reconstruction of the third X-shaped IgG homodimer by IPET. (A) Seven representative tilt images of an individual X-shaped particle are displayed in the first column from the left. Using IPET, the tilt images (after CTF correction) were gradually aligned to a common center for 3D reconstruction via iterative refinement. Projections of raw, intermediate and final 3D reconstructions at the corresponding tilt angles are displayed in the next five columns according to their corresponding tilt angles. **(B)** The final 3D density map. **(C)** The density map was flexibly docked with IgG crystal structure by using TMD simulation. **(D)** FSC analyses (from two density maps reconstructed from odd and even numbers of tilt images) showed that the resolution of the final 3D density map was ~12.5 Å. **(E)** Five snapshots illustrated the conformational changes of IgG model during TMD simulation. Scale bars=10 nm.

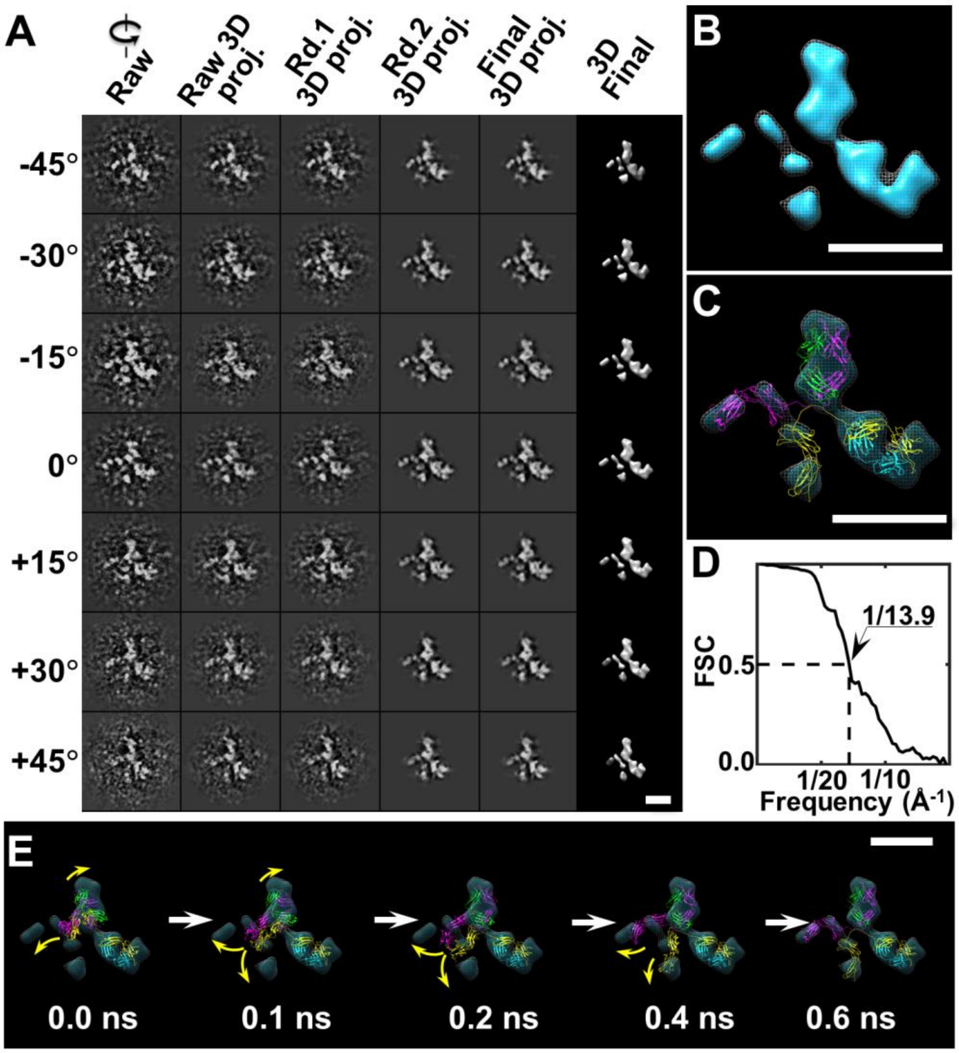

Supplementary Fig. 15 | 3D IPET reconstruction of the forth X-shaped IgG homodimers by IPET. (A) Seven representative tilt images of an individual X-shaped particle are displayed in the first column from the left. Using IPET, the tilt images (after CTF correction) were gradually aligned to a common center for 3D reconstruction via iterative refinement. Projections of raw, intermediate and final 3D reconstructions at the corresponding tilt angles are displayed in the next five columns according to their corresponding tilt angles. **(B)** The final 3D density map. **(C)** The density map was flexibly docked with IgG crystal structure by using TMD simulation. **(D)** FSC analyses (from two density maps reconstructed from odd and even numbers of tilt images) showed that the resolution of the final 3D density map was ~13.9 Å. **(E)** Five snapshots illustrated the conformational changes of IgG model during TMD simulation. Scale bars=10 nm.

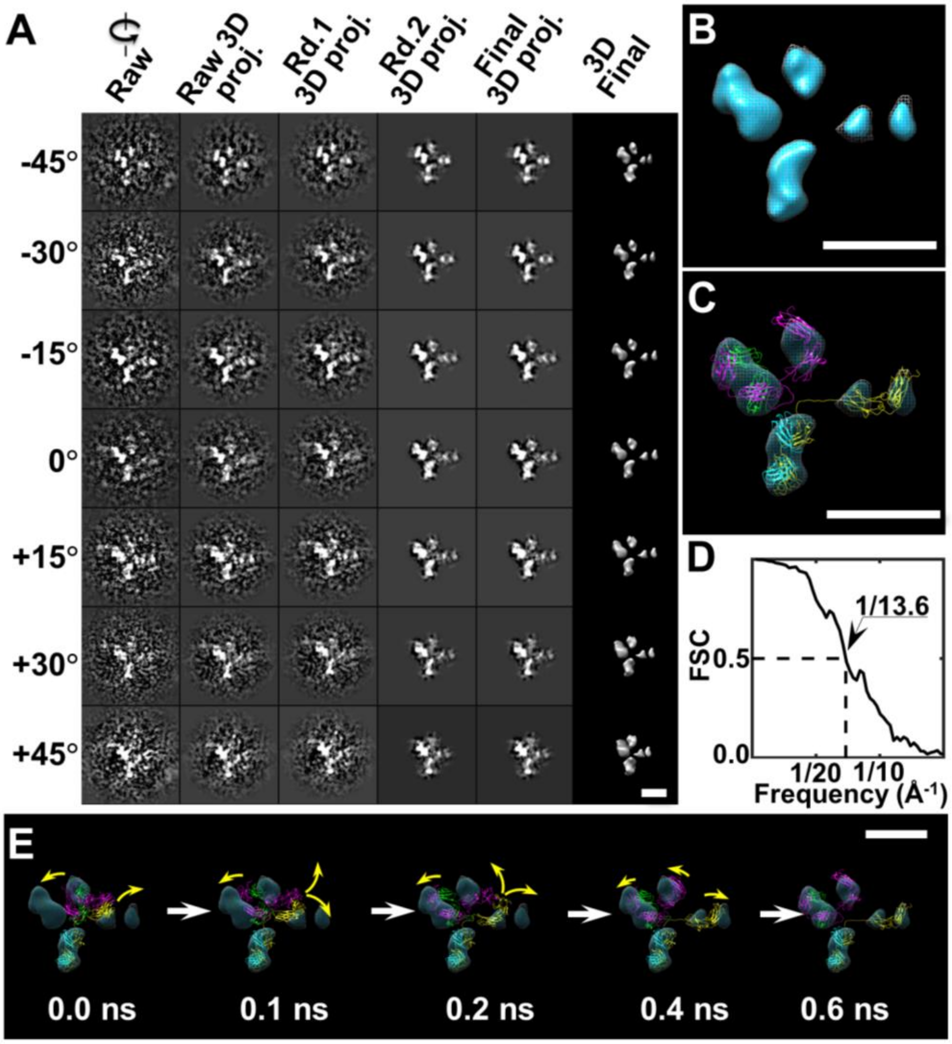

Supplementary Fig. 16 | 3D IPET reconstruction of the fifth X-shaped IgG homodimer by IPET. (A) Seven representative tilt images of an individual X-shaped particle are displayed in the first column from the left. Using IPET, the tilt images (after CTF correction) were gradually aligned to a common center for 3D reconstruction via iterative refinement. Projections of raw, intermediate and final 3D reconstructions at the corresponding tilt angles are displayed in the next five columns according to their corresponding tilt angles. **(B)** The final 3D density map. **(C)** The density map was flexibly docked with IgG crystal structure by using TMD simulation. **(D)** FSC analyses (from two density maps reconstructed from odd and even numbers of tilt images) showed that the resolution of the final 3D density map was ~13.6 Å. **(E)** Five snapshots illustrated the conformational changes of IgG model during TMD simulation. Scale bars=10 nm.

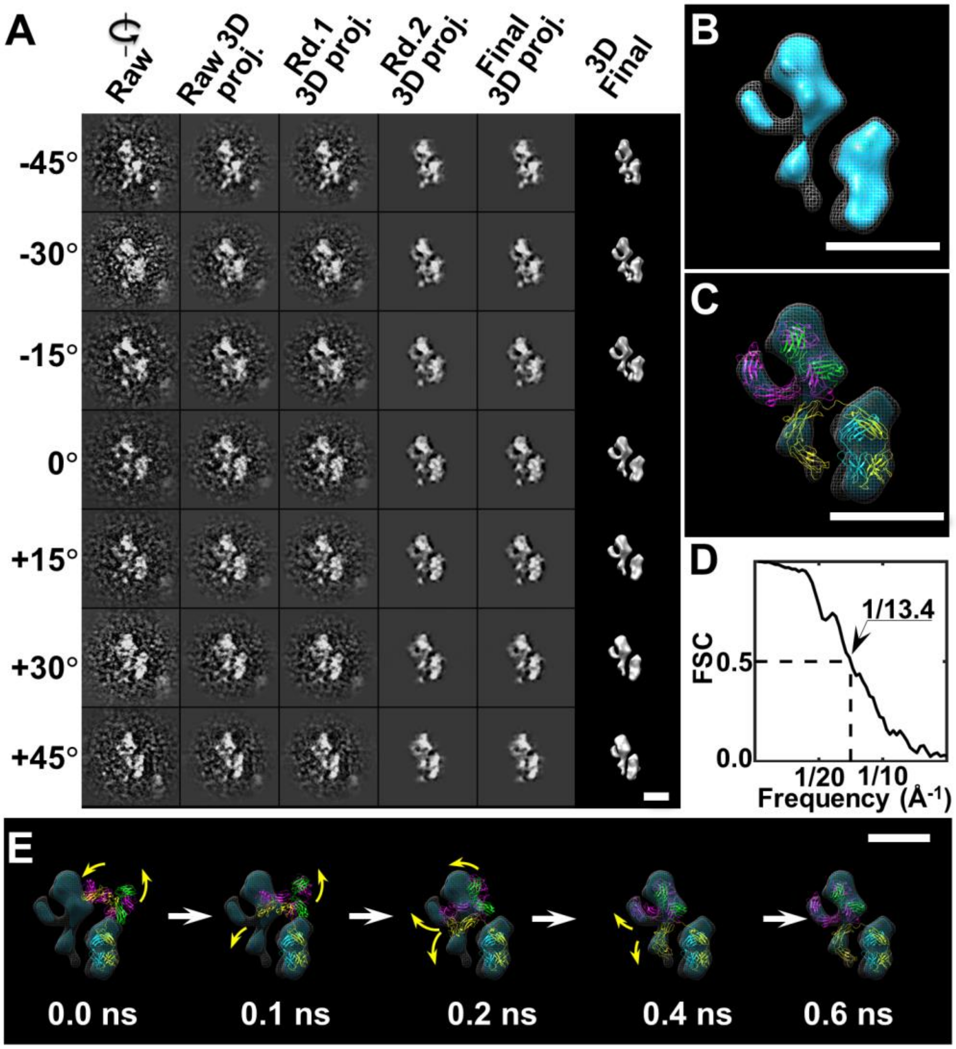

Supplementary Fig. 17 | 3D IPET reconstruction of the sixth X-shaped IgG homodimer by IPET. (A) Seven representative tilt images of an individual X-shaped particle are displayed in the first column from the left. Using IPET, the tilt images (after CTF correction) were gradually aligned to a common center for 3D reconstruction via iterative refinement. Projections of raw, intermediate and final 3D reconstructions at the corresponding tilt angles are displayed in the next five columns according to their corresponding tilt angles. **(B)** The final 3D density map. **(C)** The density map was flexibly docked with IgG crystal structure by using TMD simulation. **(D)** FSC analyses (from two density maps reconstructed from odd and even numbers of tilt images) showed that the resolution of the final 3D density map was ~13.4 Å. **(E)** Five snapshots illustrated the conformational changes of IgG model during TMD simulation. Scale bars=10 nm.

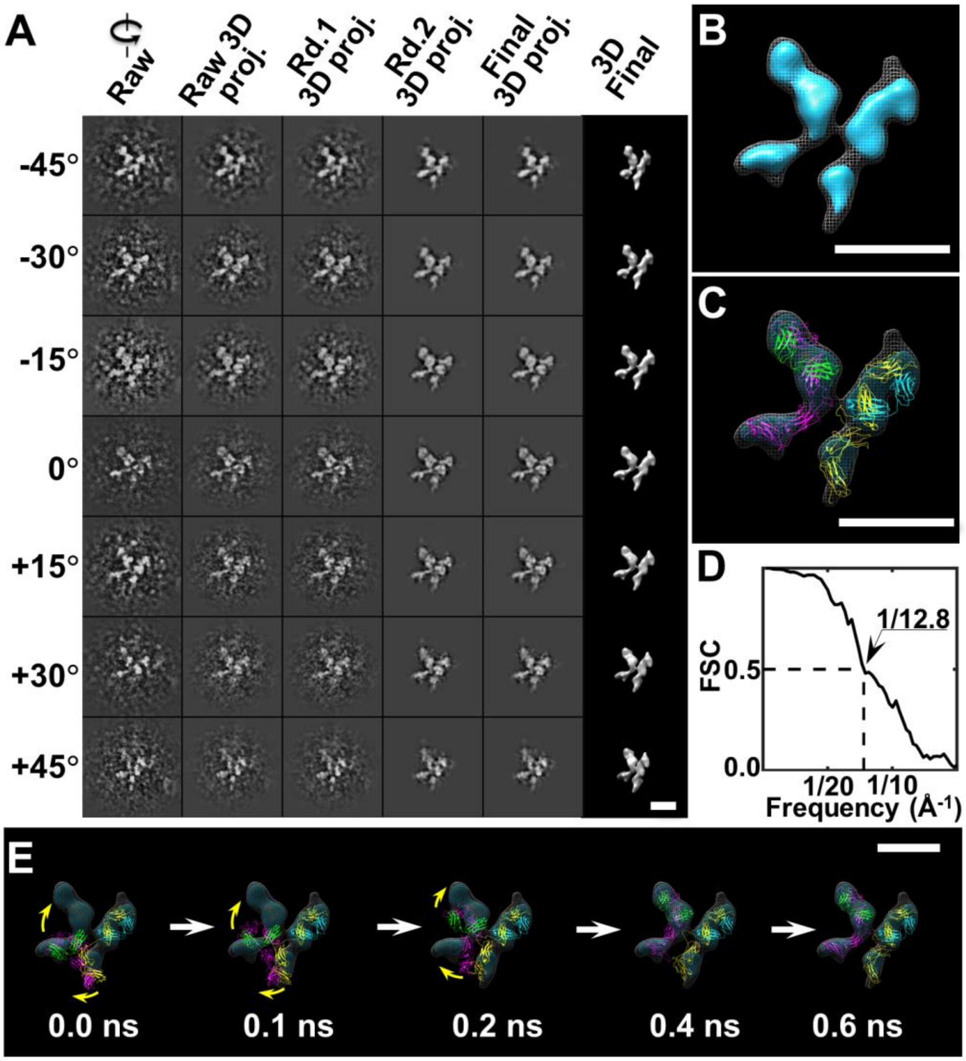

Supplementary Fig. 18 | 3D IPET reconstruction of the seventh X-shaped IgG homodimer by IPET. **(A)** Seven representative tilt images of an individual X-shaped particle are displayed in the first column from the left. Using IPET, the tilt images (after CTF correction) were gradually aligned to a common center for 3D reconstruction via iterative refinement. Projections of raw, intermediate and final 3D reconstructions at the corresponding tilt angles are displayed in the next five columns according to their corresponding tilt angles. **(B)** The final 3D density map. **(C)** The density map was flexibly docked with IgG crystal structure by using TMD simulation. **(D)** FSC analyses (from two density maps reconstructed from odd and even numbers of tilt images) showed that the resolution of the final 3D density map was ~12.8 Å. **(E)** Five snapshots illustrated the conformational changes of IgG model during TMD simulation. Scale bars=10 nm.

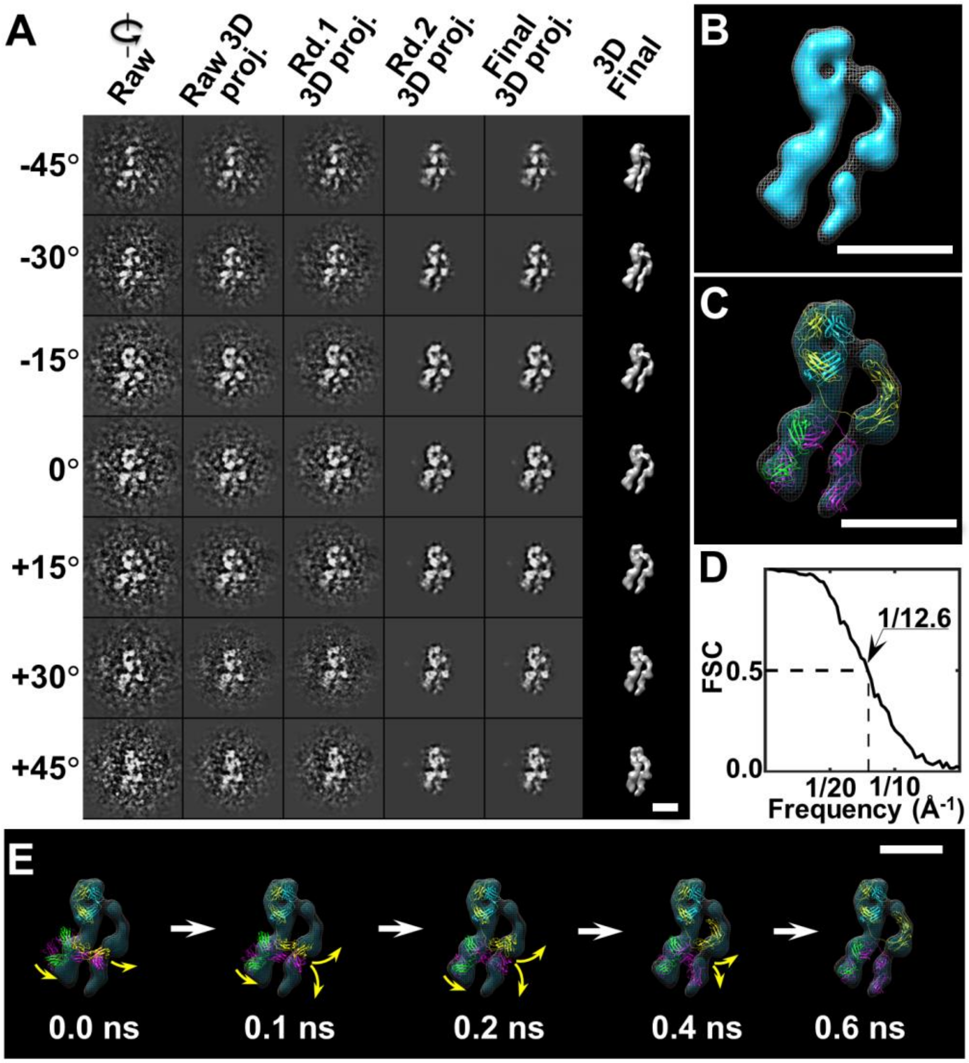

Supplementary Fig. 19 | 3D IPET reconstruction of the eighth X-shaped IgG homodimer by IPET. **(A)** Seven representative tilt images of an individual X-shaped particle are displayed in the first column from the left. Using IPET, the tilt images (after CTF correction) were gradually aligned to a common center for 3D reconstruction via iterative refinement. Projections of raw, intermediate and final 3D reconstructions at the corresponding tilt angles are displayed in the next five columns according to their corresponding tilt angles. **(B)** The final 3D density map. **(C)** The density map was flexibly docked with IgG crystal structure by using TMD simulation. **(D)** FSC analyses (from two density maps reconstructed from odd and even numbers of tilt images) showed that the resolution of the final 3D density map was ~12.6 Å. **(E)** Five snapshots illustrated the conformational changes of IgG model during TMD simulation. Scale bars=10 nm.

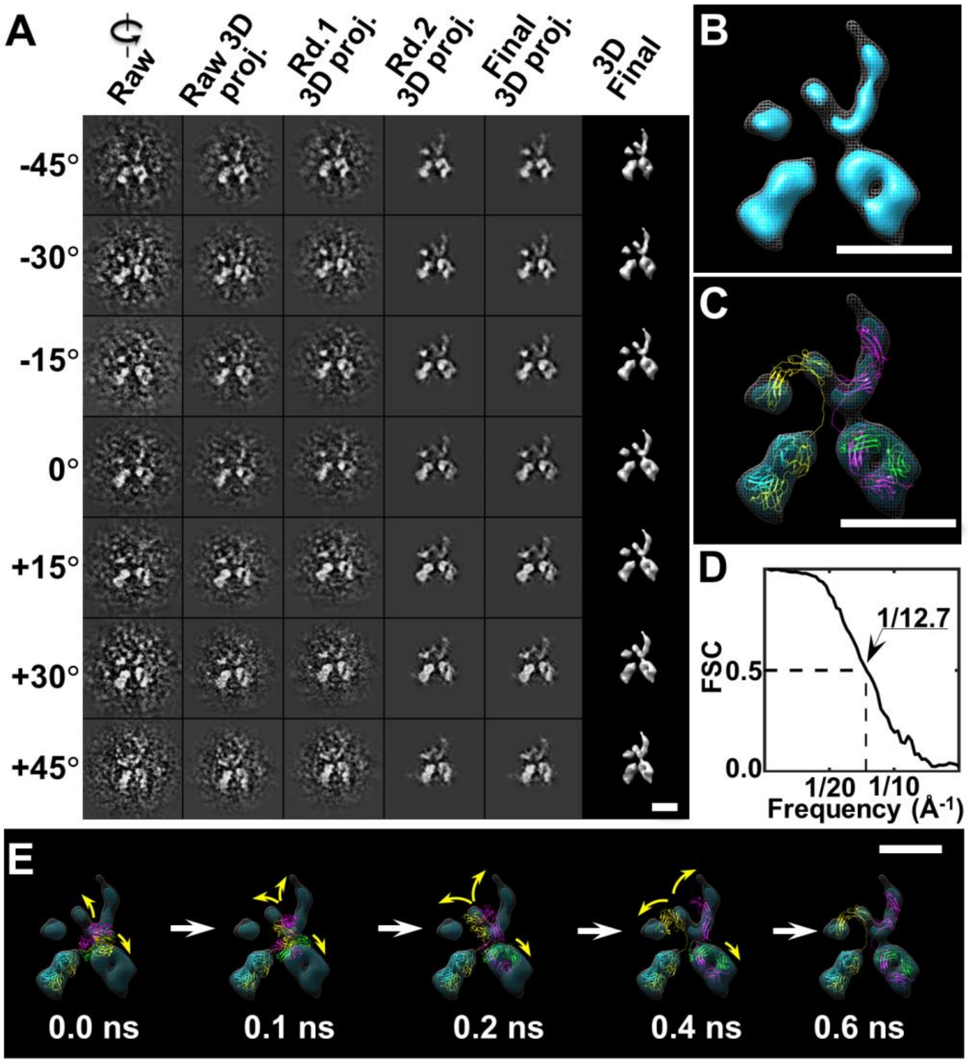

Supplementary Fig. 20 | 3D IPET reconstruction of the ninth X-shaped IgG homodimer by IPET. (A) Seven representative tilt images of an individual X-shaped particle are displayed in the first column from the left. Using IPET, the tilt images (after CTF correction) were gradually aligned to a common center for 3D reconstruction via iterative refinement. Projections of raw, intermediate and final 3D reconstructions at the corresponding tilt angles are displayed in the next five columns according to their corresponding tilt angles. **(B)** The final 3D density map. **(C)** The density map was flexibly docked with IgG crystal structure by using TMD simulation. **(D)** FSC analyses (from two density maps reconstructed from odd and even numbers of tilt images) showed that the resolution of the final 3D density map was ~12.7 Å. **(E)** Five snapshots illustrated the conformational changes of IgG model during TMD simulation. Scale bars=10 nm.

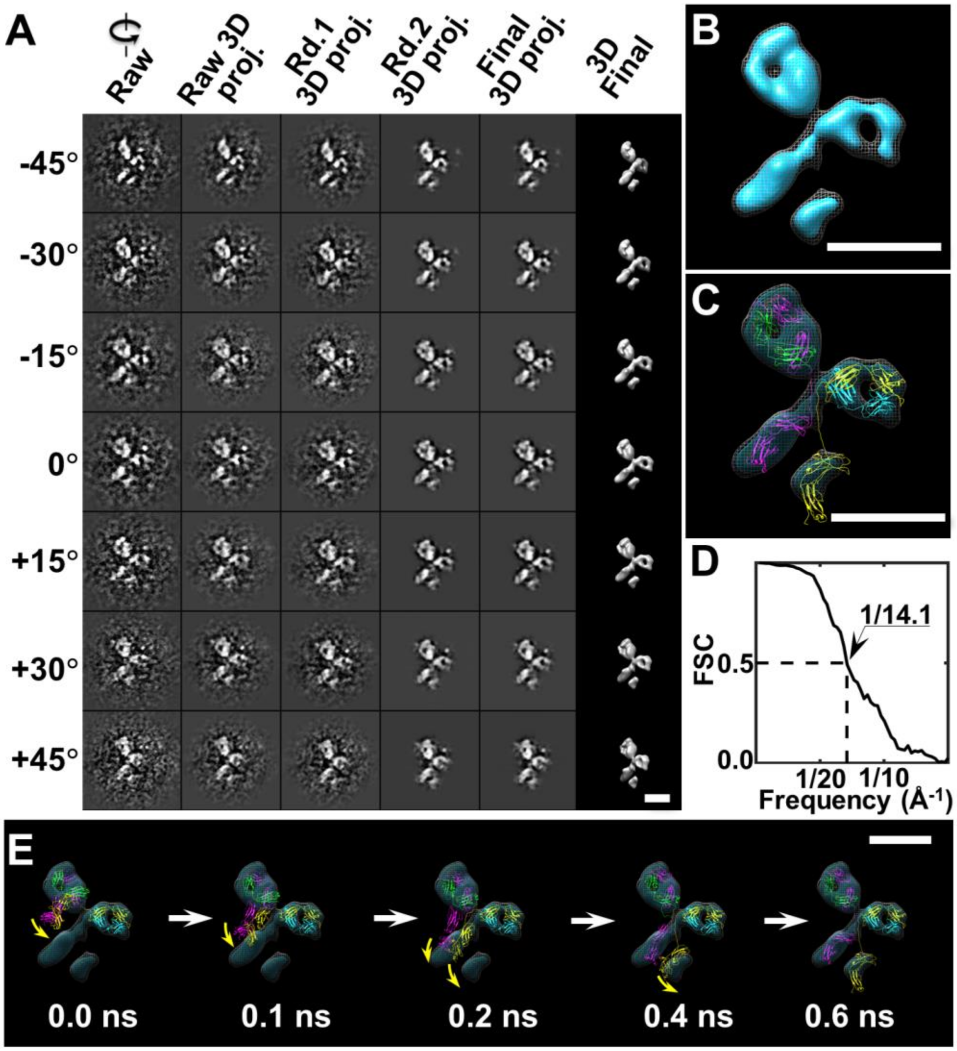

Supplementary Fig. 21 | 3D IPET reconstruction of the tenth X-shaped IgG homodimer by IPET. (A) Seven representative tilt images of an individual X-shaped particle are displayed in the first column from the left. Using IPET, the tilt images (after CTF correction) were gradually aligned to a common center for 3D reconstruction via iterative refinement. Projections of raw, intermediate and final 3D reconstructions at the corresponding tilt angles are displayed in the next five columns according to their corresponding tilt angles. **(B)** The final 3D density map. **(C)** The density map was flexibly docked with IgG crystal structure by using TMD simulation. **(D)** FSC analyses (from two density maps reconstructed from odd and even numbers of tilt images) showed that the resolution of the final 3D density map was ~14.1 Å. **(E)** Five snapshots illustrated the conformational changes of IgG model during TMD simulation. Scale bars=10 nm.

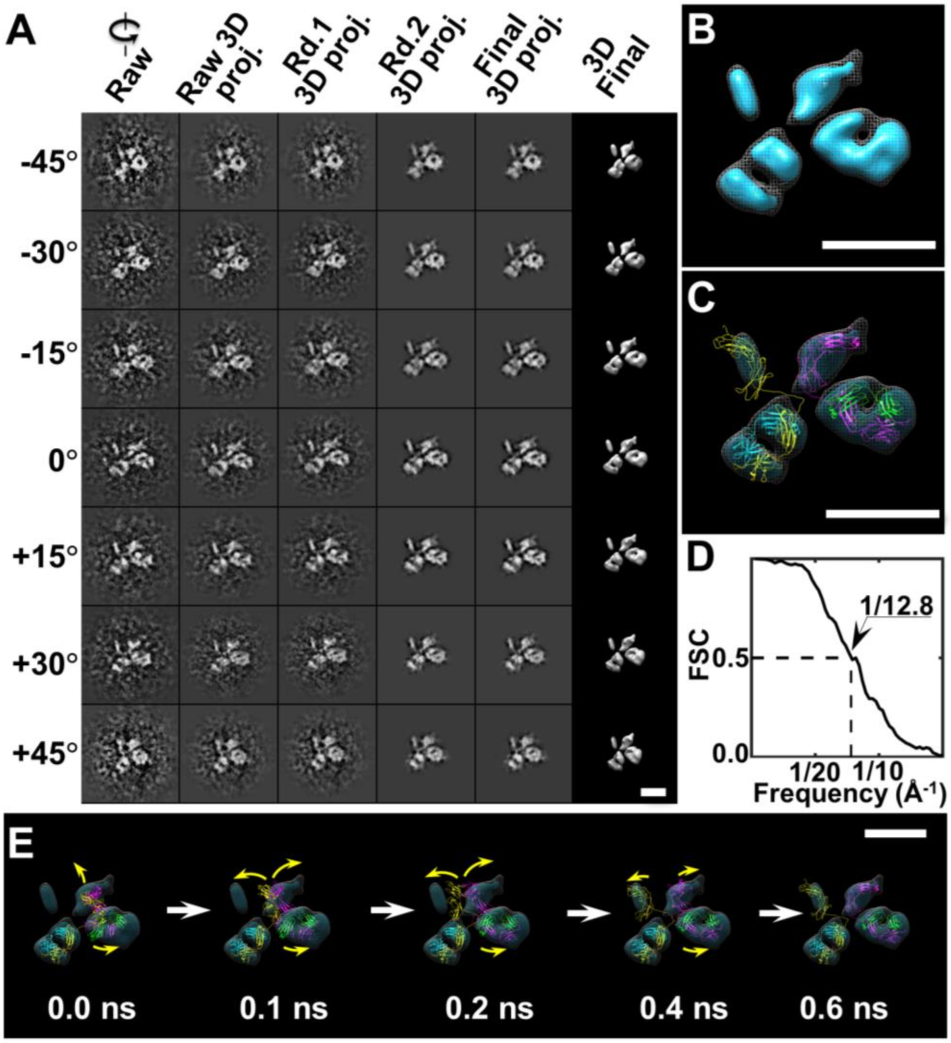

Supplementary Fig. 22 | 3D IPET reconstruction of the 11th X-shaped IgG homodimer by IPET. (A) Seven representative tilt images of an individual X-shaped particle are displayed in the first column from the left. Using IPET, the tilt images (after CTF correction) were gradually aligned to a common center for 3D reconstruction via iterative refinement. Projections of raw, intermediate and final 3D reconstructions at the corresponding tilt angles are displayed in the next five columns according to their corresponding tilt angles. **(B)** The final 3D density map. **(C)** The density map was flexibly docked with IgG crystal structure by using TMD simulation. **(D)** FSC analyses (from two density maps reconstructed from odd and even numbers of tilt images) showed that the resolution of the final 3D density map was ~12.8 Å. **(E)** Five snapshots illustrated the conformational changes of IgG model during TMD simulation. Scale bars=10 nm.

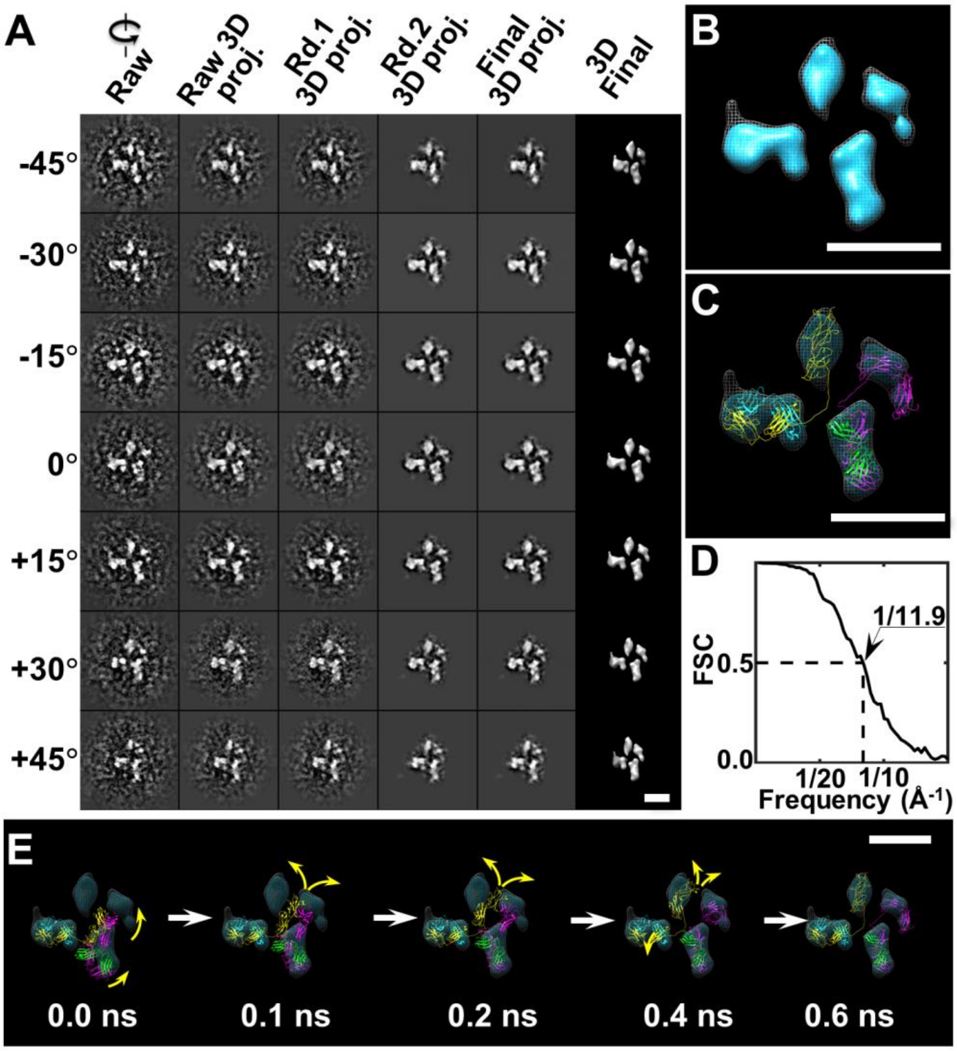

Supplementary Fig. 23 | 3D IPET reconstruction of the 12th X-shaped IgG homodimer by IPET. (A) Seven representative tilt images of an individual X-shaped particle are displayed in the first column from the left. Using IPET, the tilt images (after CTF correction) were gradually aligned to a common center for 3D reconstruction via iterative refinement. Projections of raw, intermediate and final 3D reconstructions at the corresponding tilt angles are displayed in the next five columns according to their corresponding tilt angles. **(B)** The final 3D density map. **(C)** The density map was flexibly docked with IgG crystal structure by using TMD simulation. **(D)** FSC analyses (from two density maps reconstructed from odd and even numbers of tilt images) showed that the resolution of the final 3D density map was ~11.9 Å. **(E)** Five snapshots illustrated the conformational changes of IgG model during TMD simulation. Scale bars=10 nm.

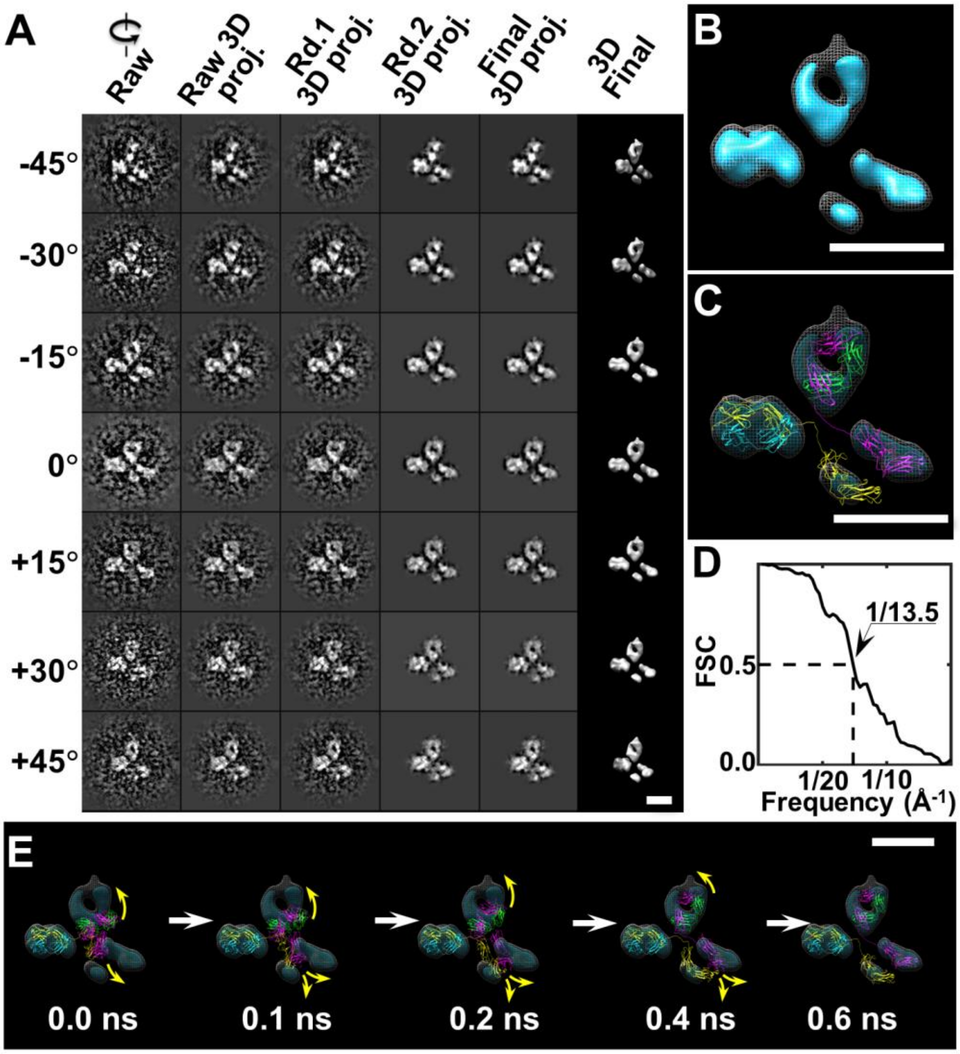

Supplementary Fig. 24 | 3D IPET reconstruction of the 13th X-shaped IgG homodimer by IPET. (A) Seven representative tilt images of an individual X-shaped particle are displayed in the first column from the left. Using IPET, the tilt images (after CTF correction) were gradually aligned to a common center for 3D reconstruction via iterative refinement. Projections of raw, intermediate and final 3D reconstructions at the corresponding tilt angles are displayed in the next five columns according to their corresponding tilt angles. **(B)** The final 3D density map. **(C)** The density map was flexibly docked with IgG crystal structure by using TMD simulation. **(D)** FSC analyses (from two density maps reconstructed from odd and even numbers of tilt images) showed that the resolution of the final 3D density map was ~13.5 Å. **(E)** Five snapshots illustrated the conformational changes of IgG model during TMD simulation. Scale bars=10 nm.

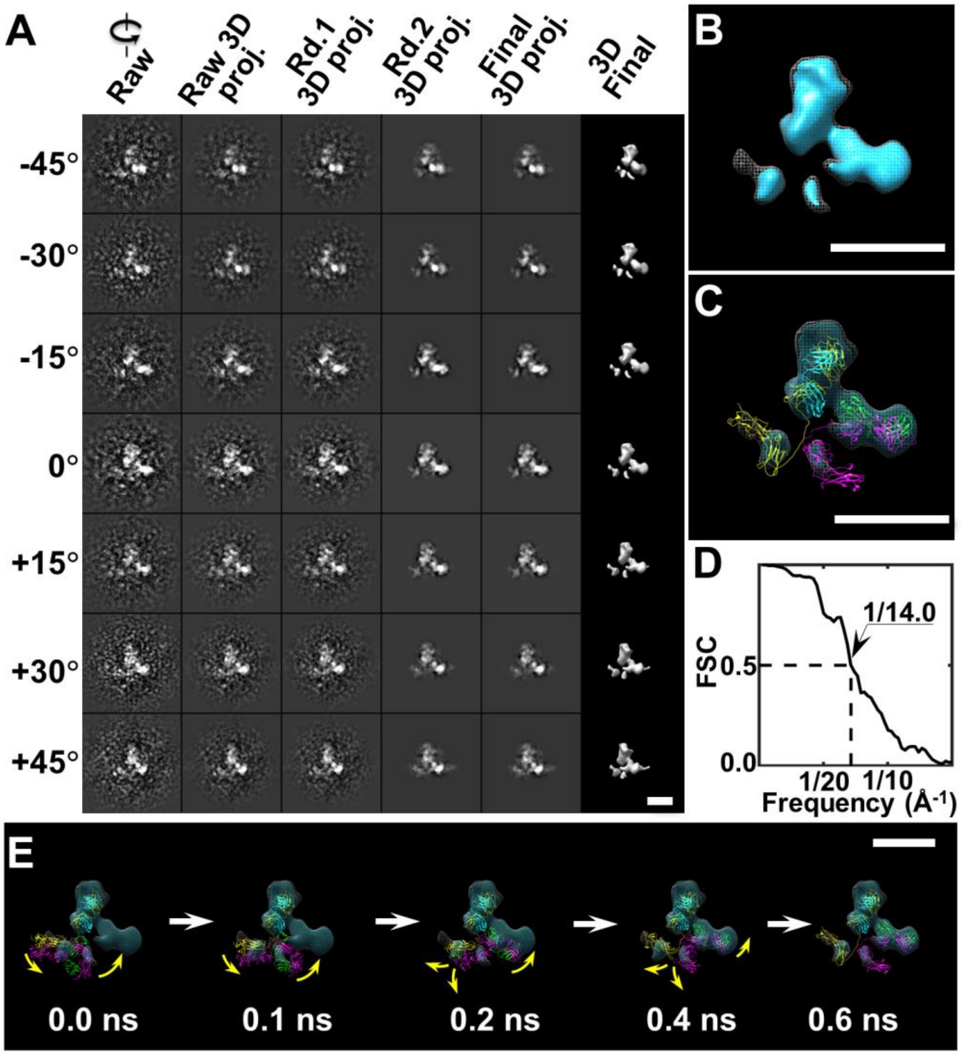

Supplementary Fig. 25 | 3D IPET reconstruction of the 14th X-shaped IgG homodimer by IPET. (A) Seven representative tilt images of an individual X-shaped particle are displayed in the first column from the left. Using IPET, the tilt images (after CTF correction) were gradually aligned to a common center for 3D reconstruction via iterative refinement. Projections of raw, intermediate and final 3D reconstructions at the corresponding tilt angles are displayed in the next five columns according to their corresponding tilt angles. **(B)** The final 3D density map. **(C)** The density map was flexibly docked with IgG crystal structure by using TMD simulation. **(D)** FSC analyses (from two density maps reconstructed from odd and even numbers of tilt images) showed that the resolution of the final 3D density map was ~14.0 Å. **(E)** Five snapshots illustrated the conformational changes of IgG model during TMD simulation. Scale bars=10 nm.

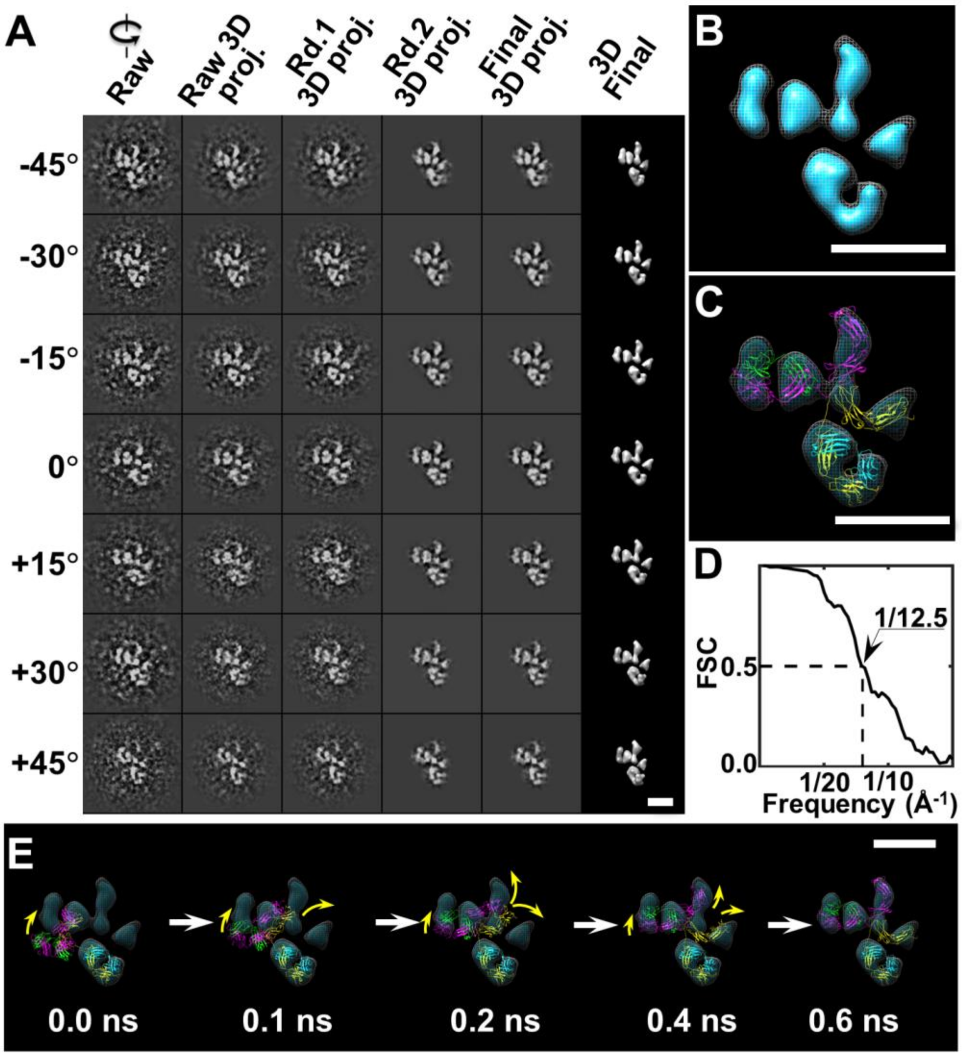

Supplementary Fig. 26 | 3D IPET reconstruction of the 15th X-shaped IgG homodimer by IPET. (A) Seven representative tilt images of an individual X-shaped particle are displayed in the first column from the left. Using IPET, the tilt images (after CTF correction) were gradually aligned to a common center for 3D reconstruction via iterative refinement. Projections of raw, intermediate and final 3D reconstructions at the corresponding tilt angles are displayed in the next five columns according to their corresponding tilt angles. **(B)** The final 3D density map. **(C)** The density map was flexibly docked with IgG crystal structure by using TMD simulation. **(D)** FSC analyses (from two density maps reconstructed from odd and even numbers of tilt images) showed that the resolution of the final 3D density map was ~12.5 Å. **(E)** Five snapshots illustrated the conformational changes of IgG model during TMD simulation. Scale bars=10 nm.

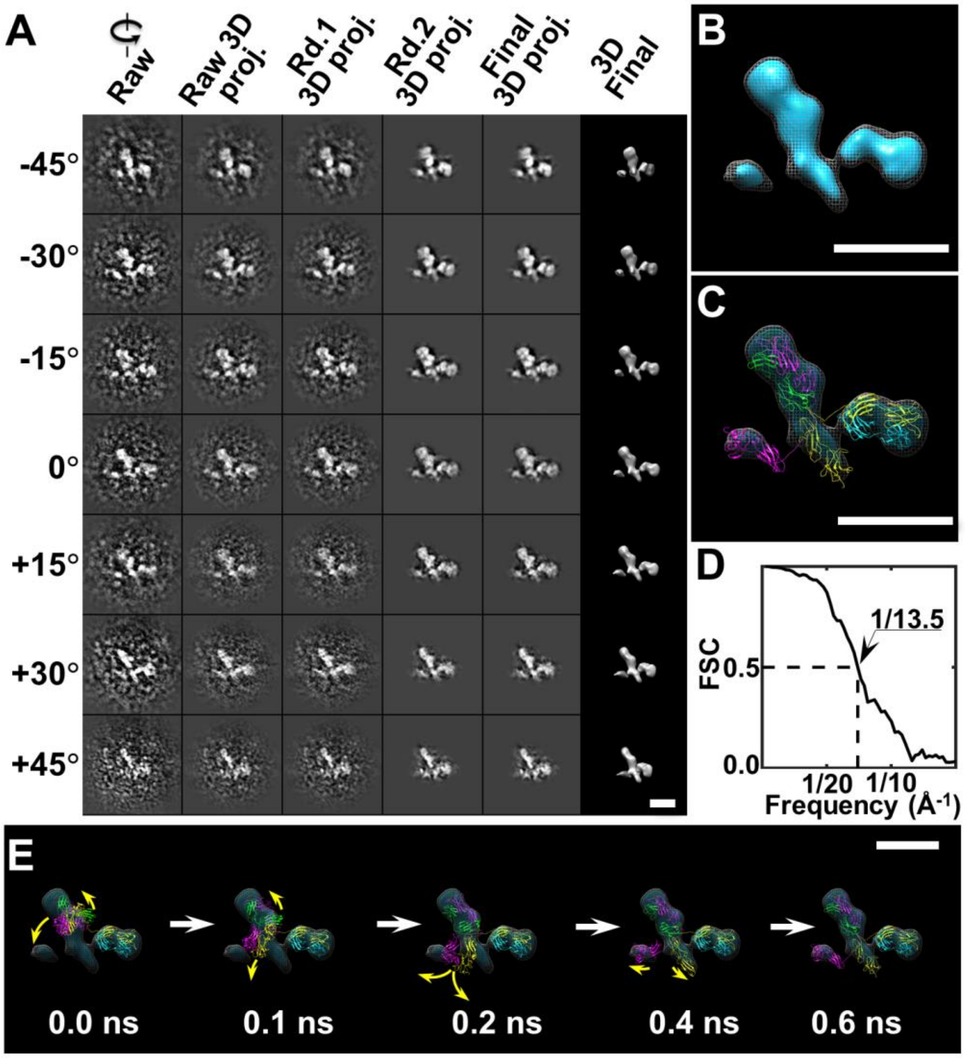

Supplementary Fig. 27 | 3D IPET reconstruction of the 16th X-shaped IgG homodimer by IPET. (A) Seven representative tilt images of an individual X-shaped particle are displayed in the first column from the left. Using IPET, the tilt images (after CTF correction) were gradually aligned to a common center for 3D reconstruction via iterative refinement. Projections of raw, intermediate and final 3D reconstructions at the corresponding tilt angles are displayed in the next five columns according to their corresponding tilt angles. **(B)** The final 3D density map. **(C)** The density map was flexibly docked with IgG crystal structure by using TMD simulation. **(D)** FSC analyses (from two density maps reconstructed from odd and even numbers of tilt images) showed that the resolution of the final 3D density map was ~13.5 Å. **(E)** Five snapshots illustrated the conformational changes of IgG model during TMD simulation. Scale bars=10 nm.

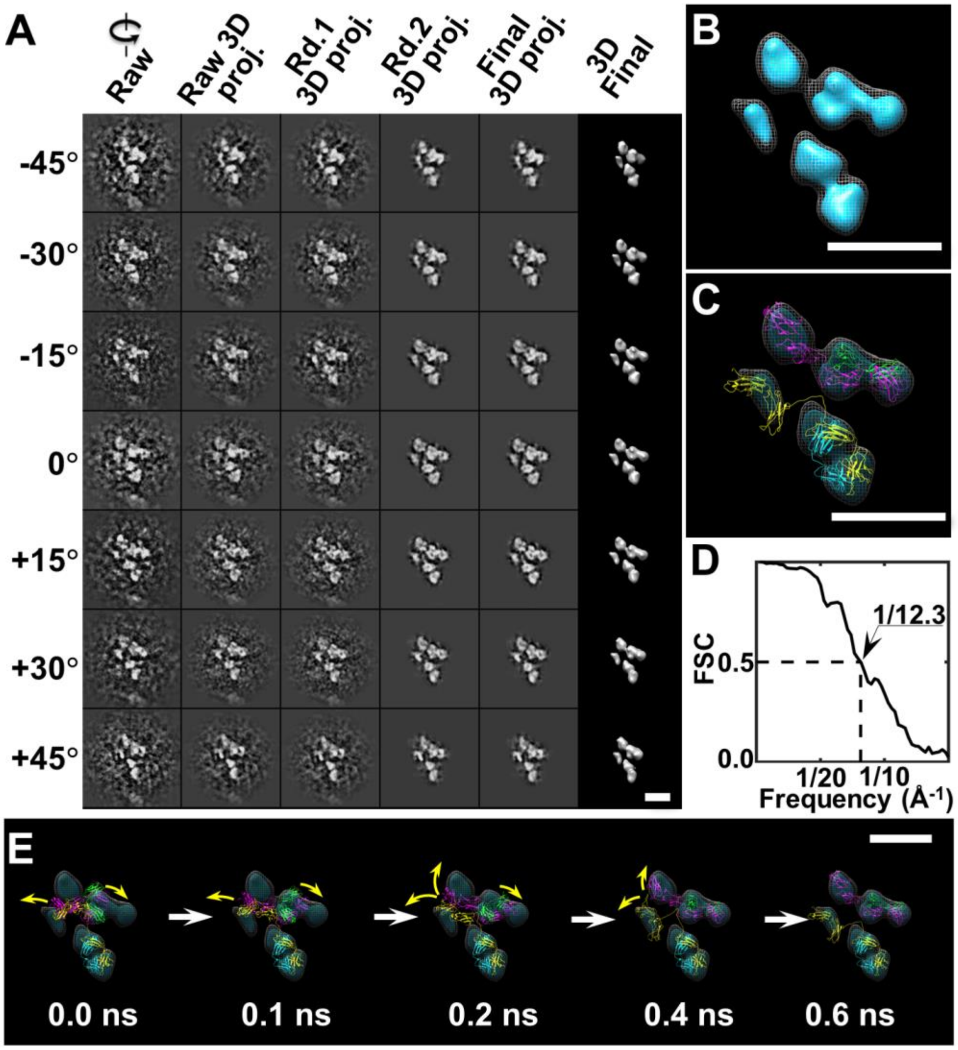

Supplementary Fig. 28 | 3D IPET reconstruction of the 17th X-shaped IgG homodimer by IPET. (A) Seven representative tilt images of an individual X-shaped particle are displayed in the first column from the left. Using IPET, the tilt images (after CTF correction) were gradually aligned to a common center for 3D reconstruction via iterative refinement. Projections of raw, intermediate and final 3D reconstructions at the corresponding tilt angles are displayed in the next five columns according to their corresponding tilt angles. **(B)** The final 3D density map. **(C)** The density map was flexibly docked with IgG crystal structure by using TMD simulation. **(D)** FSC analyses (from two density maps reconstructed from odd and even numbers of tilt images) showed that the resolution of the final 3D density map was ~12.3 Å. **(E)** Five snapshots illustrated the conformational changes of IgG model during TMD simulation. Scale bars=10 nm.

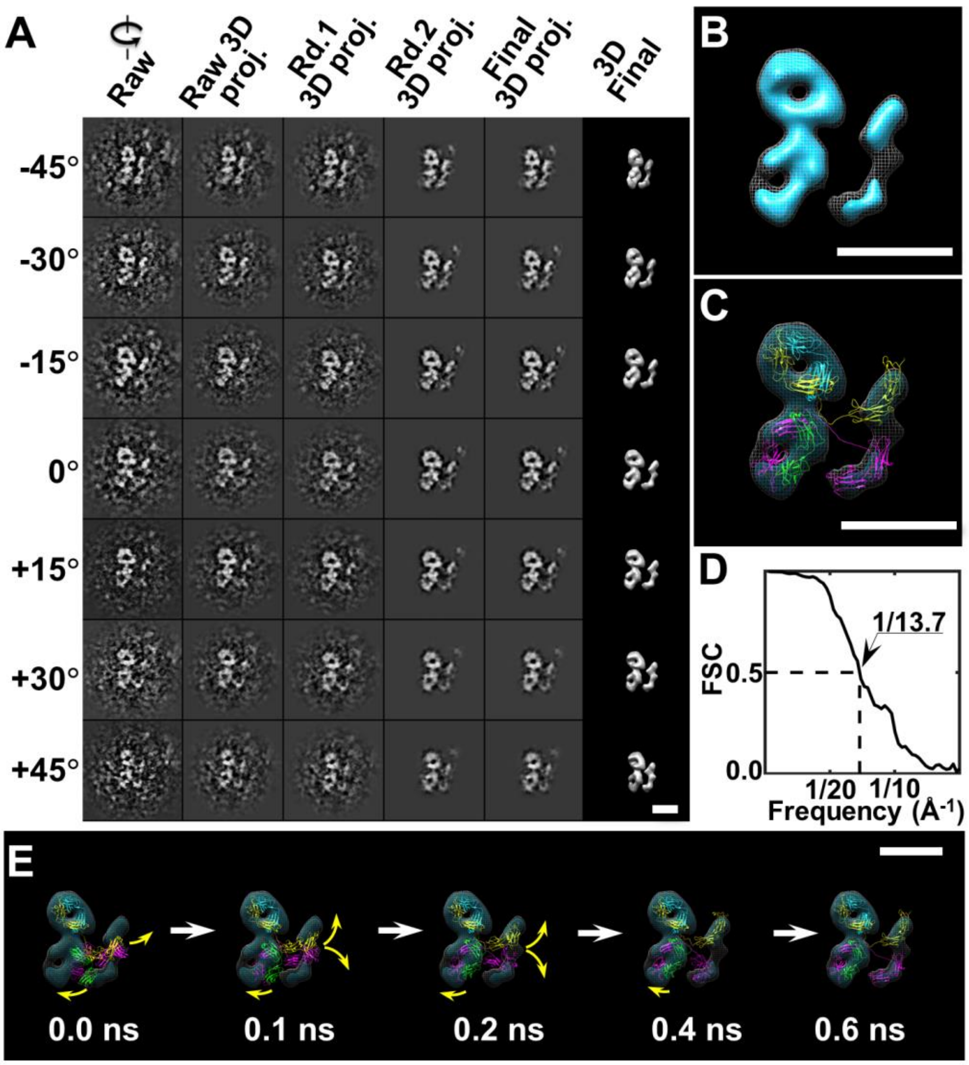

Supplementary Fig. 29 | 3D IPET reconstruction of the 18th X-shaped IgG homodimer by IPET. (A) Seven representative tilt images of an individual X-shaped particle are displayed in the first column from the left. Using IPET, the tilt images (after CTF correction) were gradually aligned to a common center for 3D reconstruction via iterative refinement. Projections of raw, intermediate and final 3D reconstructions at the corresponding tilt angles are displayed in the next five columns according to their corresponding tilt angles. **(B)** The final 3D density map. **(C)** The density map was flexibly docked with IgG crystal structure by using TMD simulation. **(D)** FSC analyses (from two density maps reconstructed from odd and even numbers of tilt images) showed that the resolution of the final 3D density map was ~13.7 Å. **(E)** Five snapshots illustrated the conformational changes of IgG model during TMD simulation. Scale bars=10 nm.

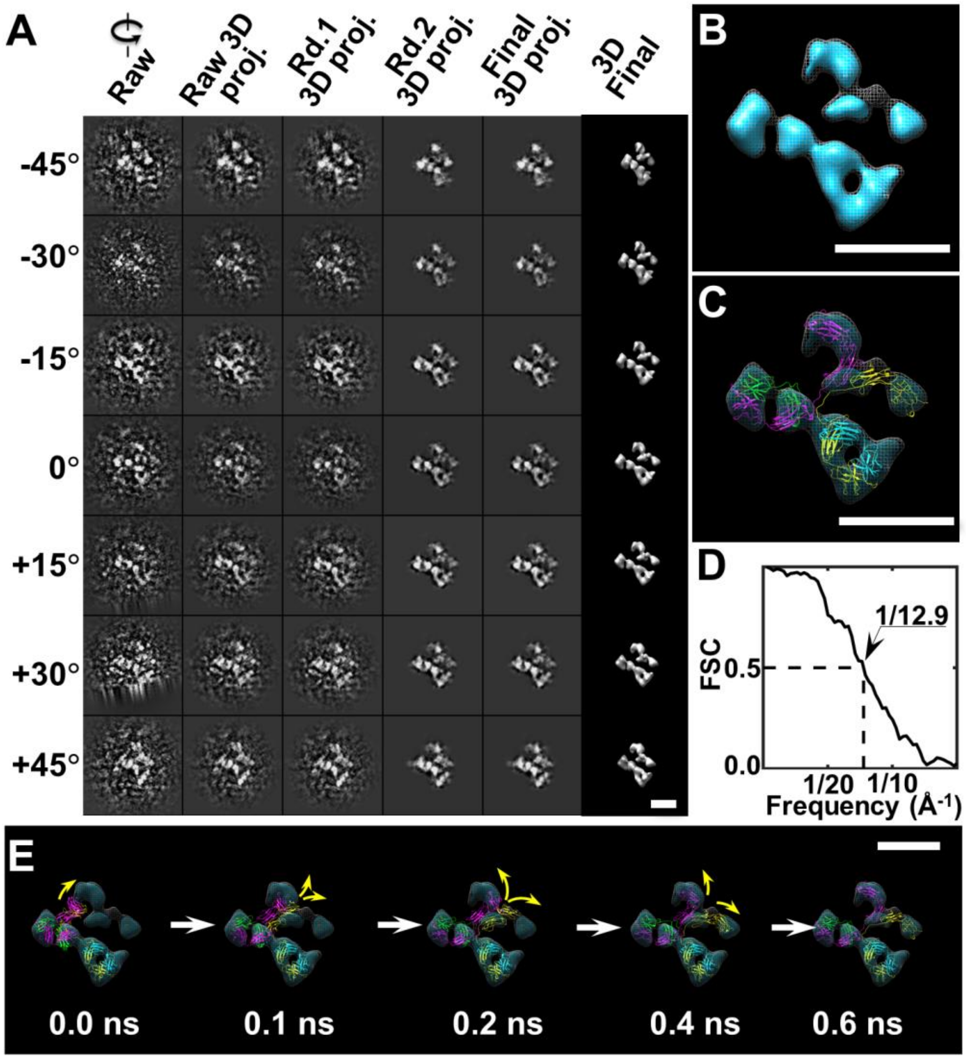

Supplementary Fig. 30 | 3D IPET reconstruction of the 19th X-shaped IgG homodimer by IPET. (A) Seven representative tilt images of an individual X-shaped particle are displayed in the first column from the left. Using IPET, the tilt images (after CTF correction) were gradually aligned to a common center for 3D reconstruction via iterative refinement. Projections of raw, intermediate and final 3D reconstructions at the corresponding tilt angles are displayed in the next five columns according to their corresponding tilt angles. **(B)** The final 3D density map. **(C)** The density map was flexibly docked with IgG crystal structure by using TMD simulation. **(D)** FSC analyses (from two density maps reconstructed from odd and even numbers of tilt images) showed that the resolution of the final 3D density map was ~12.9 Å. **(E)** Five snapshots illustrated the conformational changes of IgG model during TMD simulation. Scale bars=10 nm.

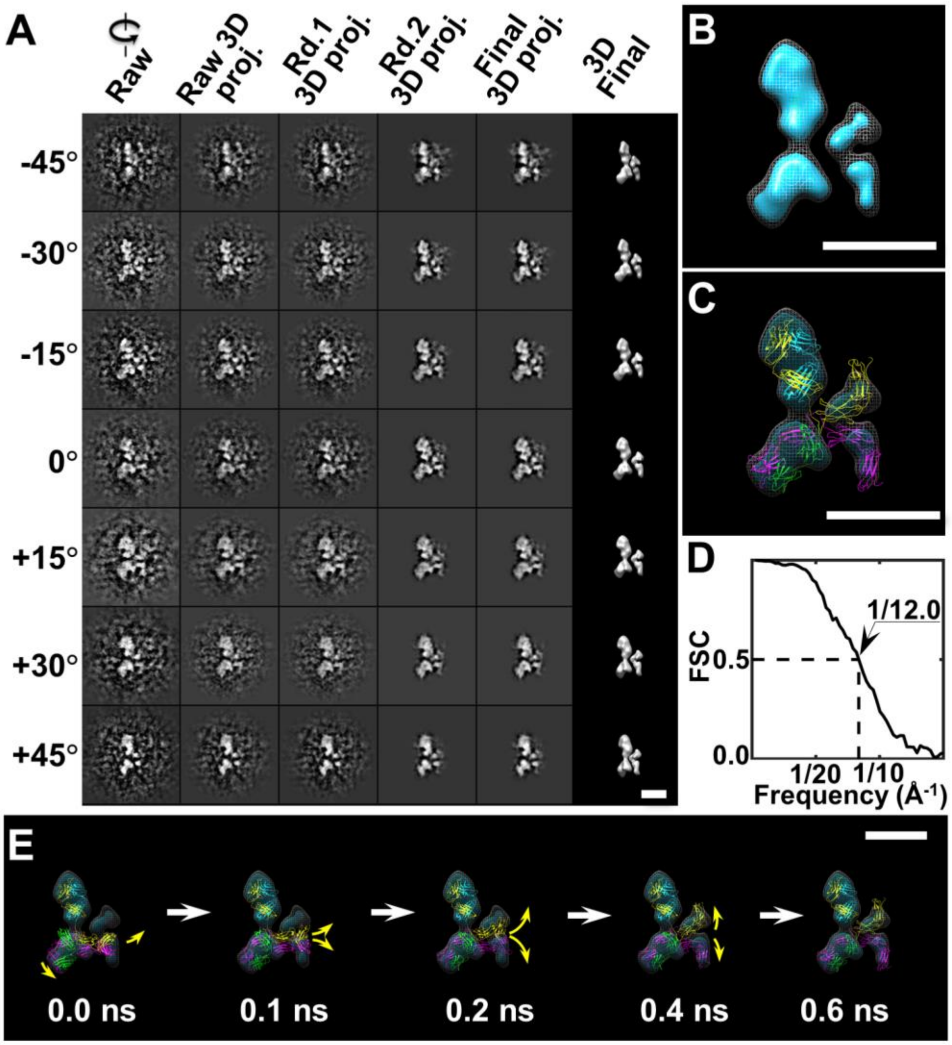

Supplementary Fig. 31 | 3D IPET reconstruction of the 20th X-shaped IgG homodimer by IPET. (A) Seven representative tilt images of an individual X-shaped particle are displayed in the first column from the left. Using IPET, the tilt images (after CTF correction) were gradually aligned to a common center for 3D reconstruction via iterative refinement. Projections of raw, intermediate and final 3D reconstructions at the corresponding tilt angles are displayed in the next five columns according to their corresponding tilt angles. **(B)** The final 3D density map. **(C)** The density map was flexibly docked with IgG crystal structure by using TMD simulation. **(D)** FSC analyses (from two density maps reconstructed from odd and even numbers of tilt images) showed that the resolution of the final 3D density map was ~12.0 Å. **(E)** Five snapshots illustrated the conformational changes of IgG model during TMD simulation. Scale bars=10 nm.

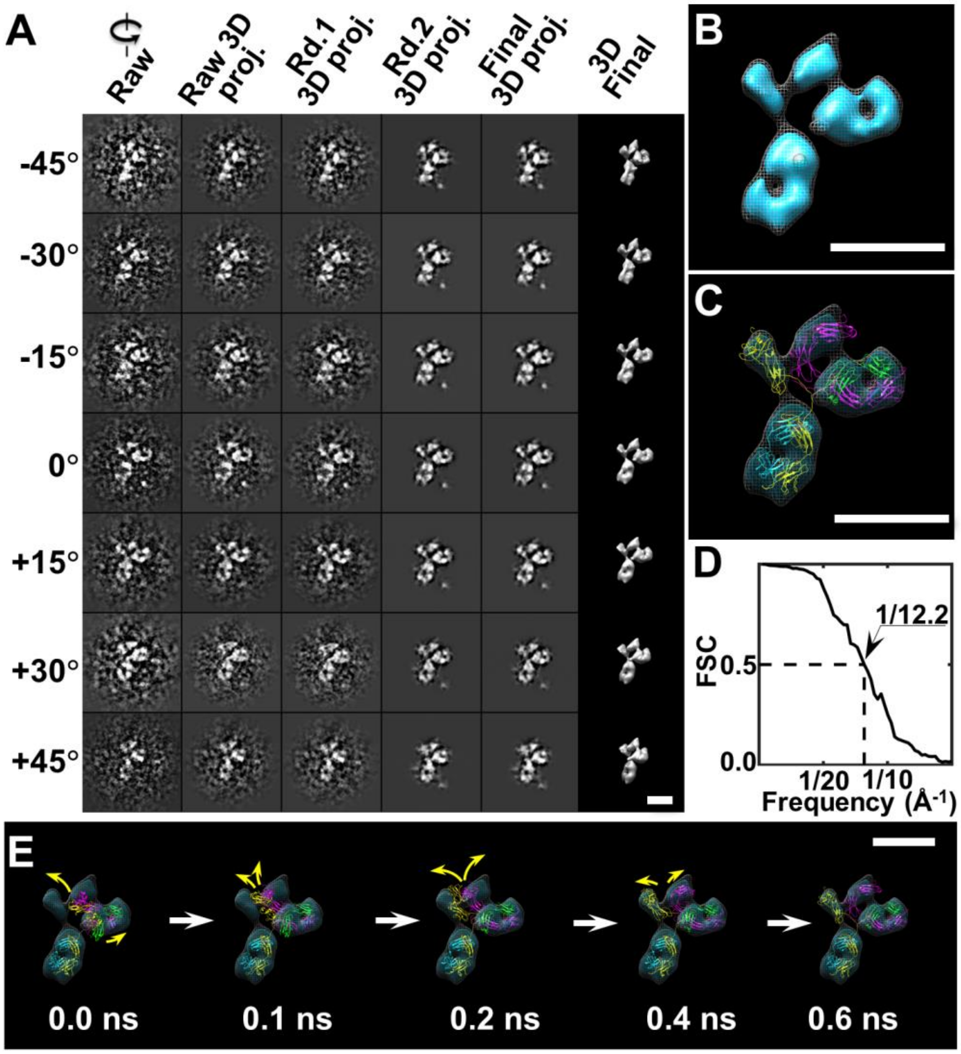

Supplementary Fig. 32 | 3D IPET reconstruction of the 21st X-shaped IgG homodimer by IPET. (A) Seven representative tilt images of an individual X-shaped particle are displayed in the first column from the left. Using IPET, the tilt images (after CTF correction) were gradually aligned to a common center for 3D reconstruction via iterative refinement. Projections of raw, intermediate and final 3D reconstructions at the corresponding tilt angles are displayed in the next five columns according to their corresponding tilt angles. **(B)** The final 3D density map. **(C)** The density map was flexibly docked with IgG crystal structure by using TMD simulation. **(D)** FSC analyses (from two density maps reconstructed from odd and even numbers of tilt images) showed that the resolution of the final 3D density map was ~12.2 Å. **(E)** Five snapshots illustrated the conformational changes of IgG model during TMD simulation. Scale bars=10 nm.

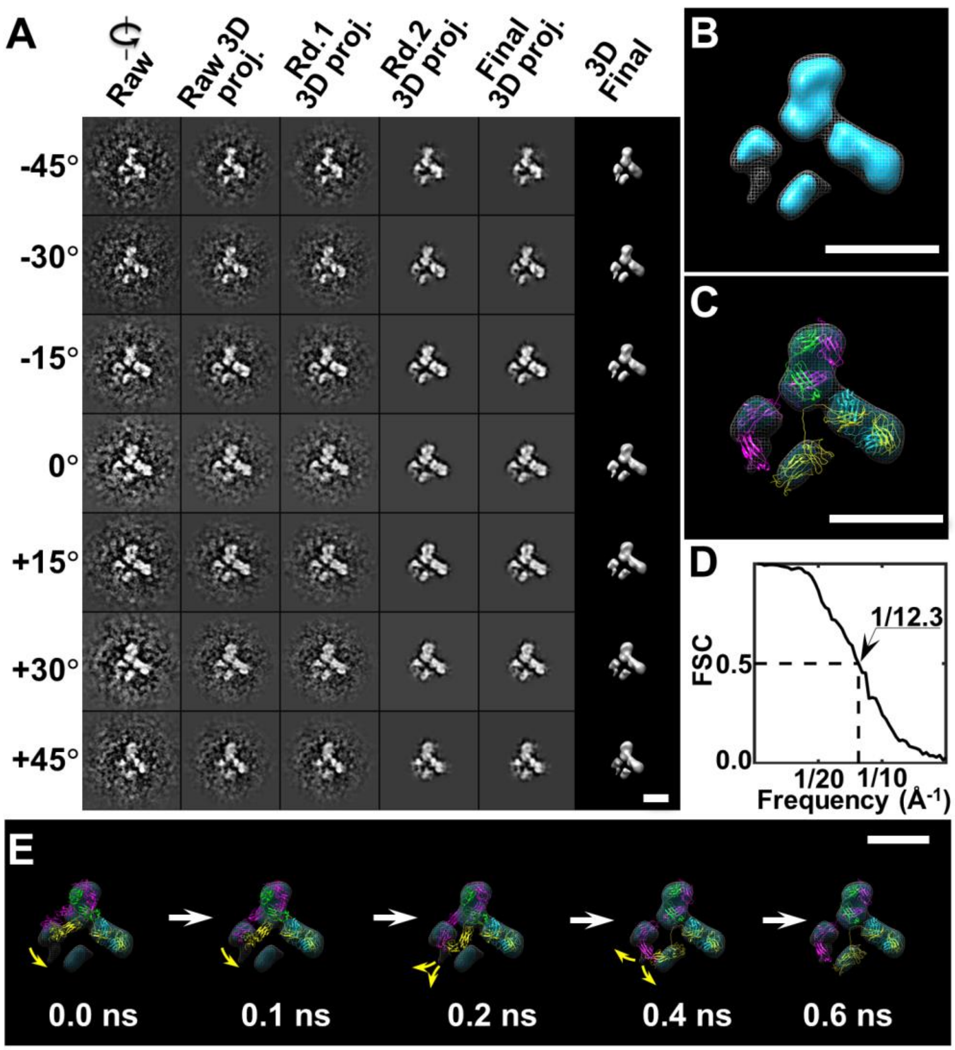

Supplementary Fig. 33 | 3D IPET reconstruction of the 22nd X-shaped IgG homodimer by IPET. (A) Seven representative tilt images of an individual X-shaped particle are displayed in the first column from the left. Using IPET, the tilt images (after CTF correction) were gradually aligned to a common center for 3D reconstruction via iterative refinement. Projections of raw, intermediate and final 3D reconstructions at the corresponding tilt angles are displayed in the next five columns according to their corresponding tilt angles. **(B)** The final 3D density map. **(C)** The density map was flexibly docked with IgG crystal structure by using TMD simulation. **(D)** FSC analyses (from two density maps reconstructed from odd and even numbers of tilt images) showed that the resolution of the final 3D density map was ~13.3 Å. **(E)** Five snapshots illustrated the conformational changes of IgG model during TMD simulation. Scale bars=10 nm.

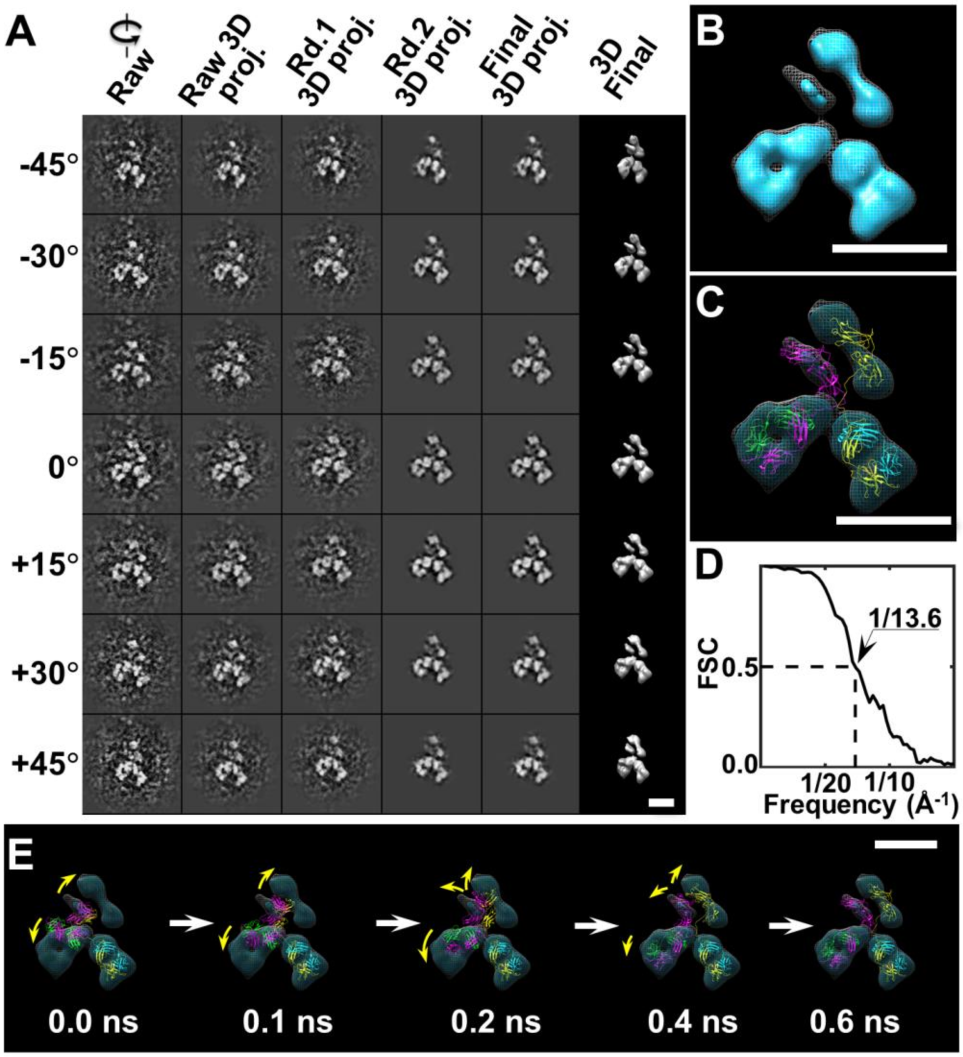

Supplementary Fig. 34 | 3D IPET reconstruction of the 23rd X-shaped IgG homodimer by IPET. (A) Seven representative tilt images of an individual X-shaped particle are displayed in the first column from the left. Using IPET, the tilt images (after CTF correction) were gradually aligned to a common center for 3D reconstruction via iterative refinement. Projections of raw, intermediate and final 3D reconstructions at the corresponding tilt angles are displayed in the next five columns according to their corresponding tilt angles. **(B)** The final 3D density map. **(C)** The density map was flexibly docked with IgG crystal structure by using TMD simulation. **(D)** FSC analyses (from two density maps reconstructed from odd and even numbers of tilt images) showed that the resolution of the final 3D density map was ~13.6 Å. **(E)** Five snapshots illustrated the conformational changes of IgG model during TMD simulation. Scale bars=10 nm.

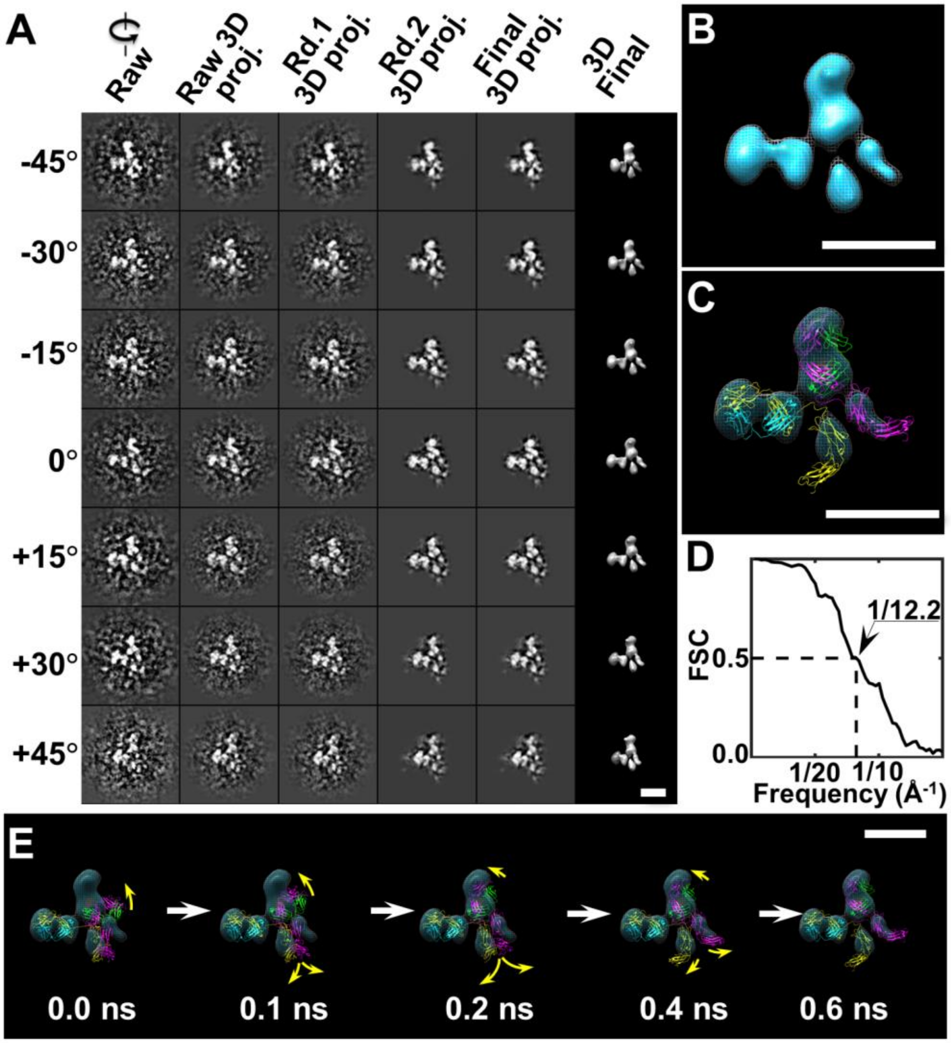

Supplementary Fig. 35 | 3D IPET reconstruction of the 24th X-shaped IgG homodimer by IPET. (A) Seven representative tilt images of an individual X-shaped particle are displayed in the first column from the left. Using IPET, the tilt images (after CTF correction) were gradually aligned to a common center for 3D reconstruction via iterative refinement. Projections of raw, intermediate and final 3D reconstructions at the corresponding tilt angles are displayed in the next five columns according to their corresponding tilt angles. **(B)** The final 3D density map. **(C)** The density map was flexibly docked with IgG crystal structure by using TMD simulation. **(D)** FSC analyses (from two density maps reconstructed from odd and even numbers of tilt images) showed that the resolution of the final 3D density map was ~12.2 Å. **(E)** Five snapshots illustrated the conformational changes of IgG model during TMD simulation. Scale bars=10 nm.

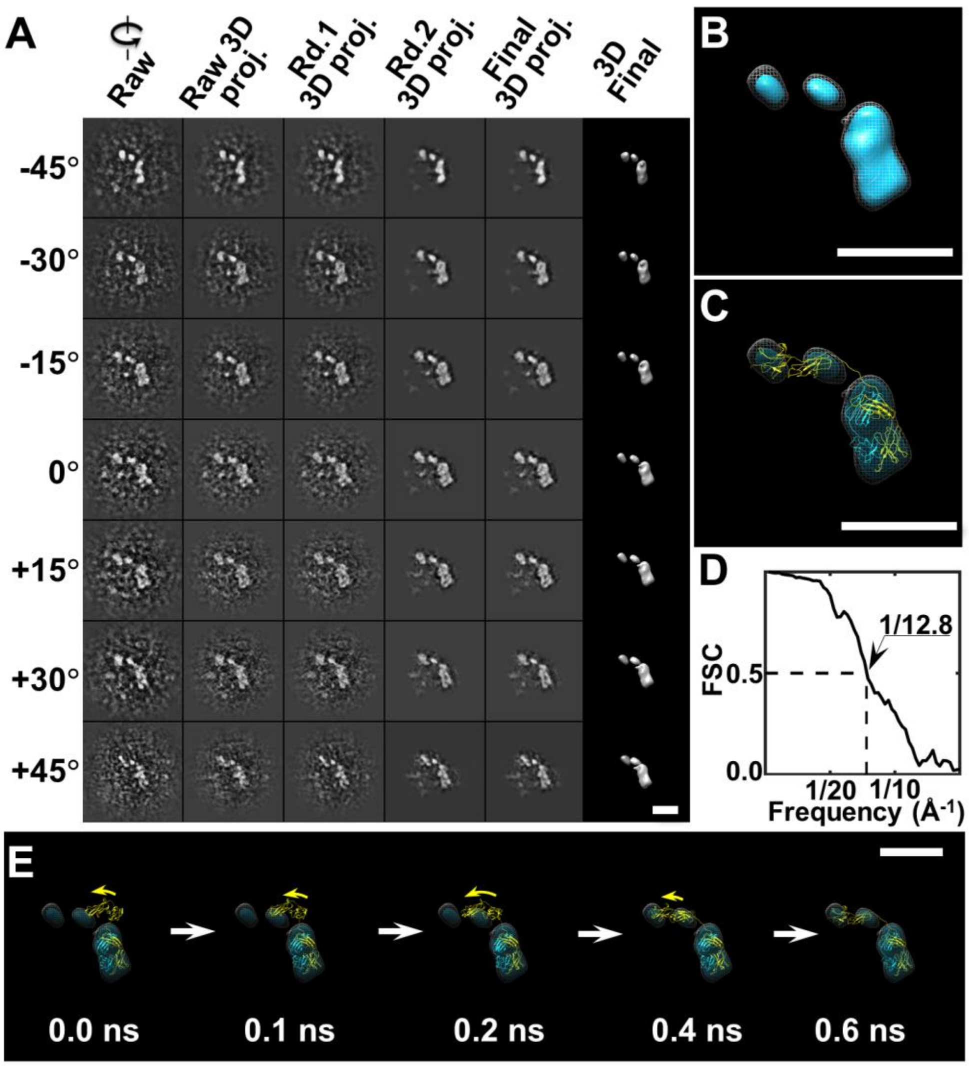

Supplementary Fig. 36 | 3D IPET reconstruction of the first i-shaped half-IgG by IPET. (A) Seven representative tilt images of an individual i-shaped particle are displayed in the first column from the left. Using IPET, the tilt images (after CTF correction) were gradually aligned to a common center for 3D reconstruction via iterative refinement. Projections of raw, intermediate and final 3D reconstructions at the corresponding tilt angles are displayed in the next five columns according to their corresponding tilt angles. **(B)** The final 3D density map. **(C)** The density map was flexibly docked with IgG crystal structure by using TMD simulation. **(D)** FSC analyses (from two density maps reconstructed from odd and even numbers of tilt images) showed that the resolution of the final 3D density map was ~12.8 Å. **(E)** Five snapshots illustrated the conformational changes of IgG model during TMD simulation. Scale bars=10 nm.

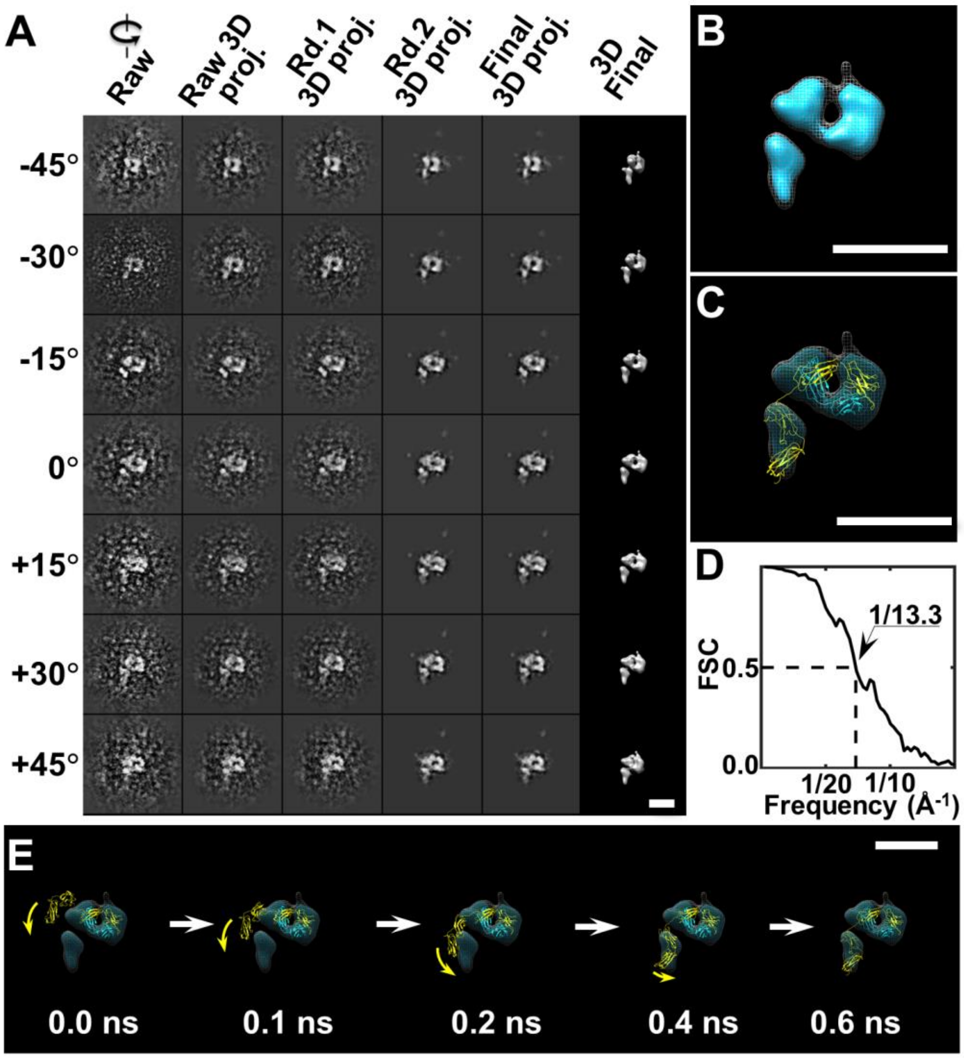

Supplementary Fig. 37 | 3D IPET reconstruction of the second i-shaped half-IgG by IPET. (A) Seven representative tilt images of an individual i-shaped particle are displayed in the first column from the left. Using IPET, the tilt images (after CTF correction) were gradually aligned to a common center for 3D reconstruction via iterative refinement. Projections of raw, intermediate and final 3D reconstructions at the corresponding tilt angles are displayed in the next five columns according to their corresponding tilt angles. **(B)** The final 3D density map. **(C)** The density map was flexibly docked with IgG crystal structure by using TMD simulation. **(D)** FSC analyses (from two density maps reconstructed from odd and even numbers of tilt images) showed that the resolution of the final 3D density map was ~13.3 Å. **(E)** Five snapshots illustrated the conformational changes of IgG model during TMD simulation. Scale bars=10 nm.

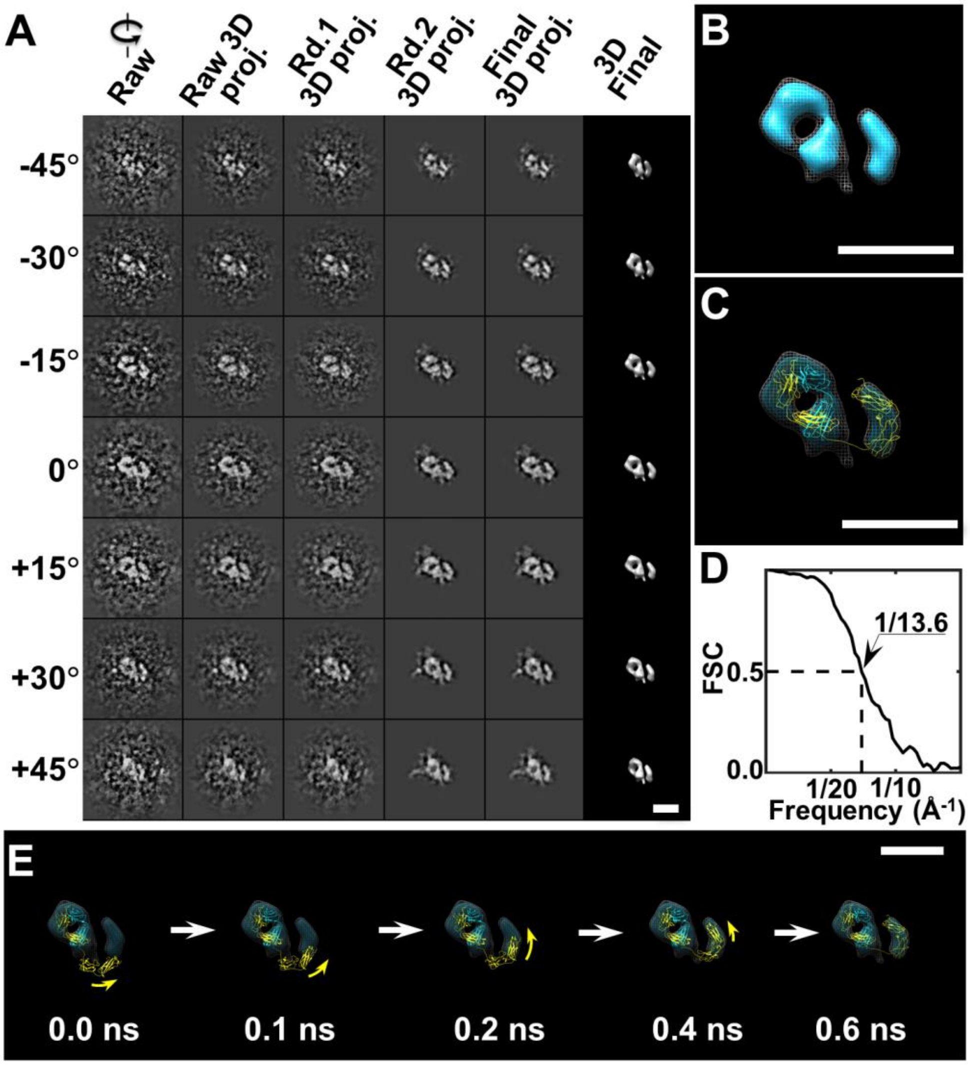

Supplementary Fig. 38 | 3D IPET reconstruction of the third i-shaped half-IgG by IPET. (A) Seven representative tilt images of an individual i-shaped particle are displayed in the first column from the left. Using IPET, the tilt images (after CTF correction) were gradually aligned to a common center for 3D reconstruction via iterative refinement. Projections of raw, intermediate and final 3D reconstructions at the corresponding tilt angles are displayed in the next five columns according to their corresponding tilt angles. **(B)** The final 3D density map. **(C)** The density map was flexibly docked with IgG crystal structure by using TMD simulation. **(D)** FSC analyses (from two density maps reconstructed from odd and even numbers of tilt images) showed that the resolution of the final 3D density map was ~13.6 Å. **(E)** Five snapshots illustrated the conformational changes of IgG model during TMD simulation. Scale bars=10 nm.

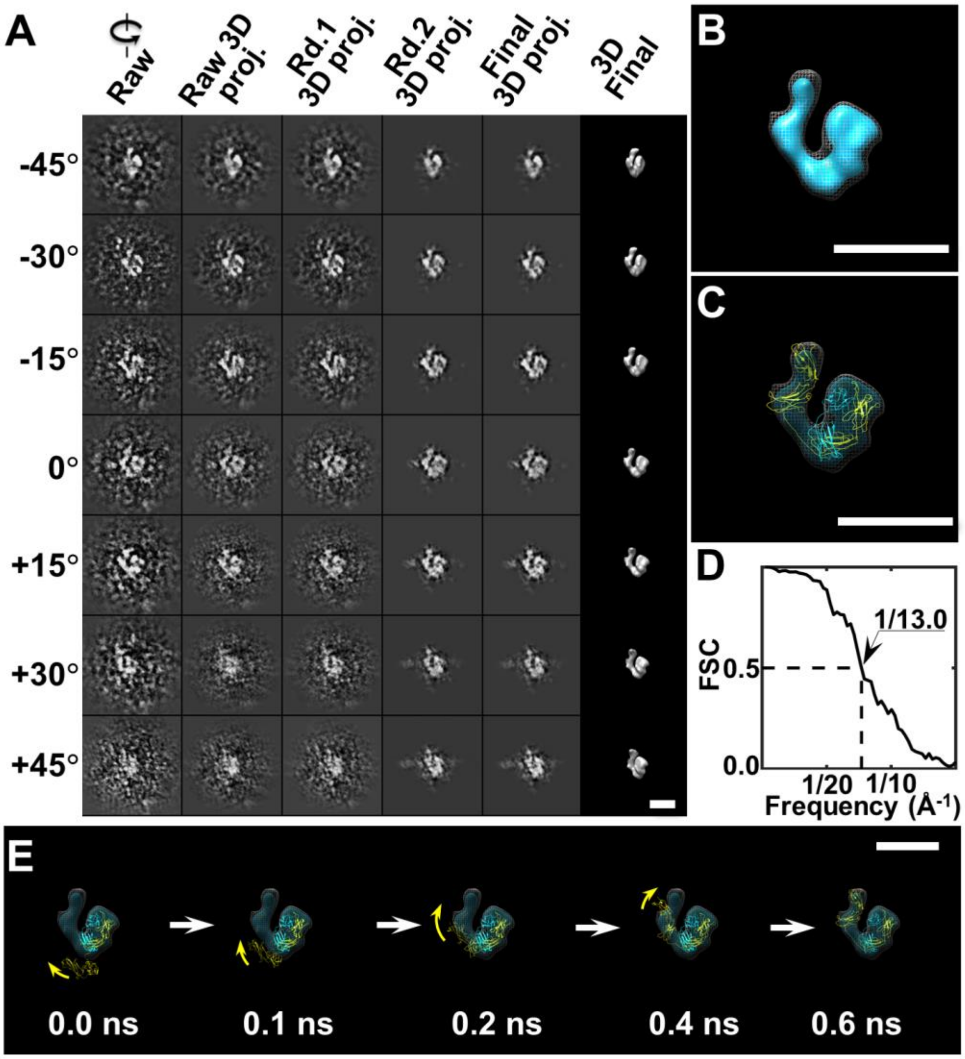

Supplementary Fig. 39 | 3D IPET reconstruction of the forth i-shaped half-IgG by IPET. (A) Seven representative tilt images of an individual i-shaped particle are displayed in the first column from the left. Using IPET, the tilt images (after CTF correction) were gradually aligned to a common center for 3D reconstruction via iterative refinement. Projections of raw, intermediate and final 3D reconstructions at the corresponding tilt angles are displayed in the next five columns according to their corresponding tilt angles. **(B)** The final 3D density map. **(C)** The density map was flexibly docked with IgG crystal structure by using TMD simulation. **(D)** FSC analyses (from two density maps reconstructed from odd and even numbers of tilt images) showed that the resolution of the final 3D density map was ~13.0 Å. **(E)** Five snapshots illustrated the conformational changes of IgG model during TMD simulation. Scale bars=10 nm.

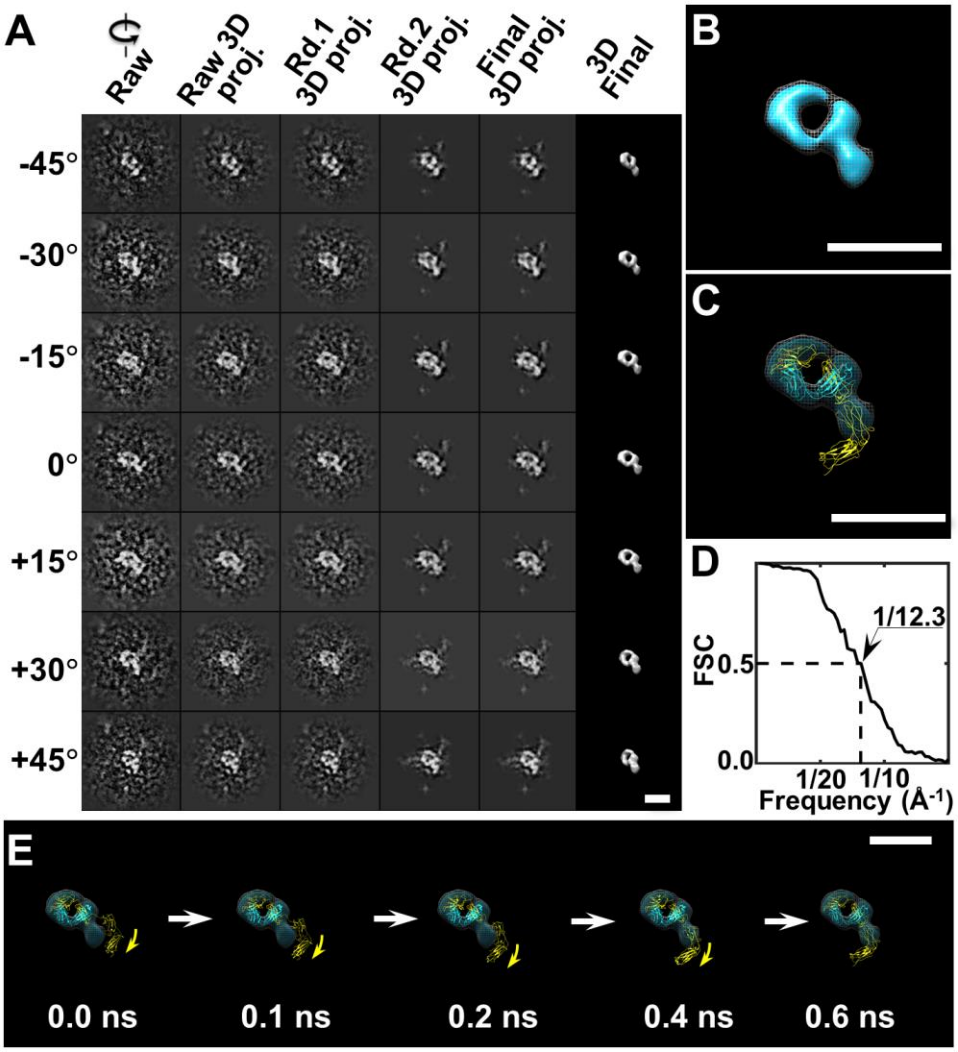

Supplementary Fig. 40 | 3D IPET reconstruction of the fifth i-shaped half-IgG by IPET. (A) Seven representative tilt images of an individual i-shaped particle are displayed in the first column from the left. Using IPET, the tilt images (after CTF correction) were gradually aligned to a common center for 3D reconstruction via iterative refinement. Projections of raw, intermediate and final 3D reconstructions at the corresponding tilt angles are displayed in the next five columns according to their corresponding tilt angles. **(B)** The final 3D density map. **(C)** The density map was flexibly docked with IgG crystal structure by using TMD simulation. **(D)** FSC analyses (from two density maps reconstructed from odd and even numbers of tilt images) showed that the resolution of the final 3D density map was ~12.3 Å. **(E)** Five snapshots illustrated the conformational changes of IgG model during TMD simulation. Scale bars=10 nm.

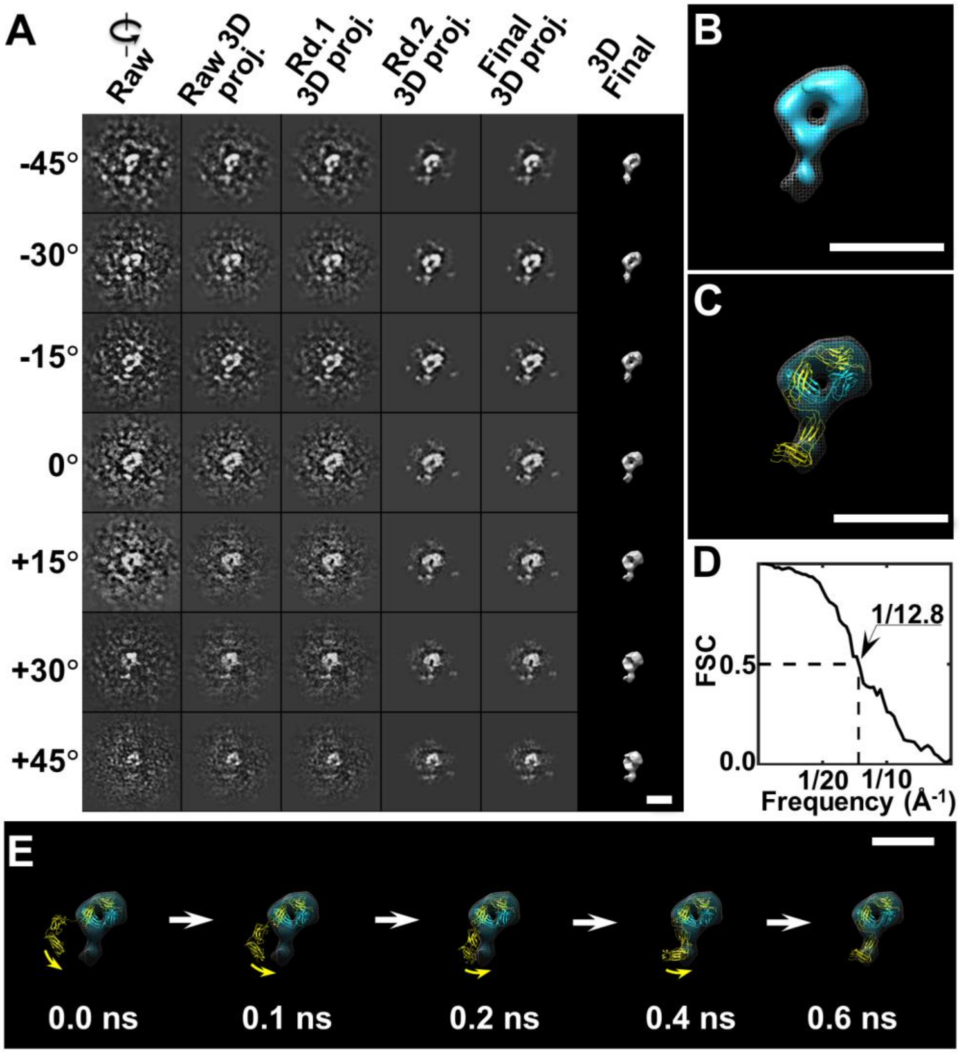

Supplementary Fig. 41 | 3D IPET reconstruction of the sixth i-shaped half-IgG by IPET. (A) Seven representative tilt images of an individual i-shaped particle are displayed in the first column from the left. Using IPET, the tilt images (after CTF correction) were gradually aligned to a common center for 3D reconstruction via iterative refinement. Projections of raw, intermediate and final 3D reconstructions at the corresponding tilt angles are displayed in the next five columns according to their corresponding tilt angles. **(B)** The final 3D density map. **(C)** The density map was flexibly docked with IgG crystal structure by using TMD simulation. **(D)** FSC analyses (from two density maps reconstructed from odd and even numbers of tilt images) showed that the resolution of the final 3D density map was ~12.8 Å. **(E)** Five snapshots illustrated the conformational changes of IgG model during TMD simulation. Scale bars=10 nm.

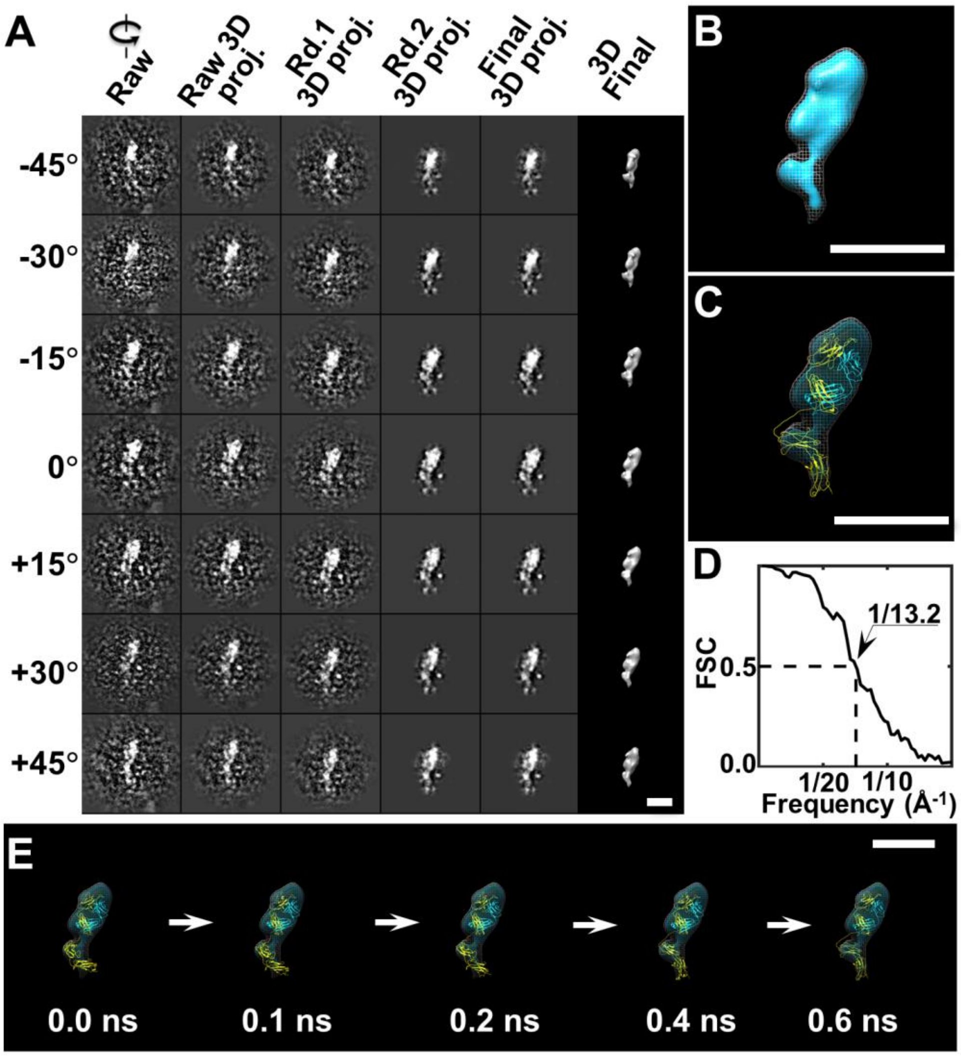

Supplementary Fig. 42 | 3D IPET reconstruction of the seventh i-shaped half-IgG by IPET. (A) Seven representative tilt images of an individual i-shaped particle are displayed in the first column from the left. Using IPET, the tilt images (after CTF correction) were gradually aligned to a common center for 3D reconstruction via iterative refinement. Projections of raw, intermediate and final 3D reconstructions at the corresponding tilt angles are displayed in the next five columns according to their corresponding tilt angles. **(B)** The final 3D density map. **(C)** The density map was flexibly docked with IgG crystal structure by using TMD simulation. **(D)** FSC analyses (from two density maps reconstructed from odd and even numbers of tilt images) showed that the resolution of the final 3D density map was ~13.2 Å. **(E)** Five snapshots illustrated the conformational changes of IgG model during TMD simulation. Scale bars=10 nm.

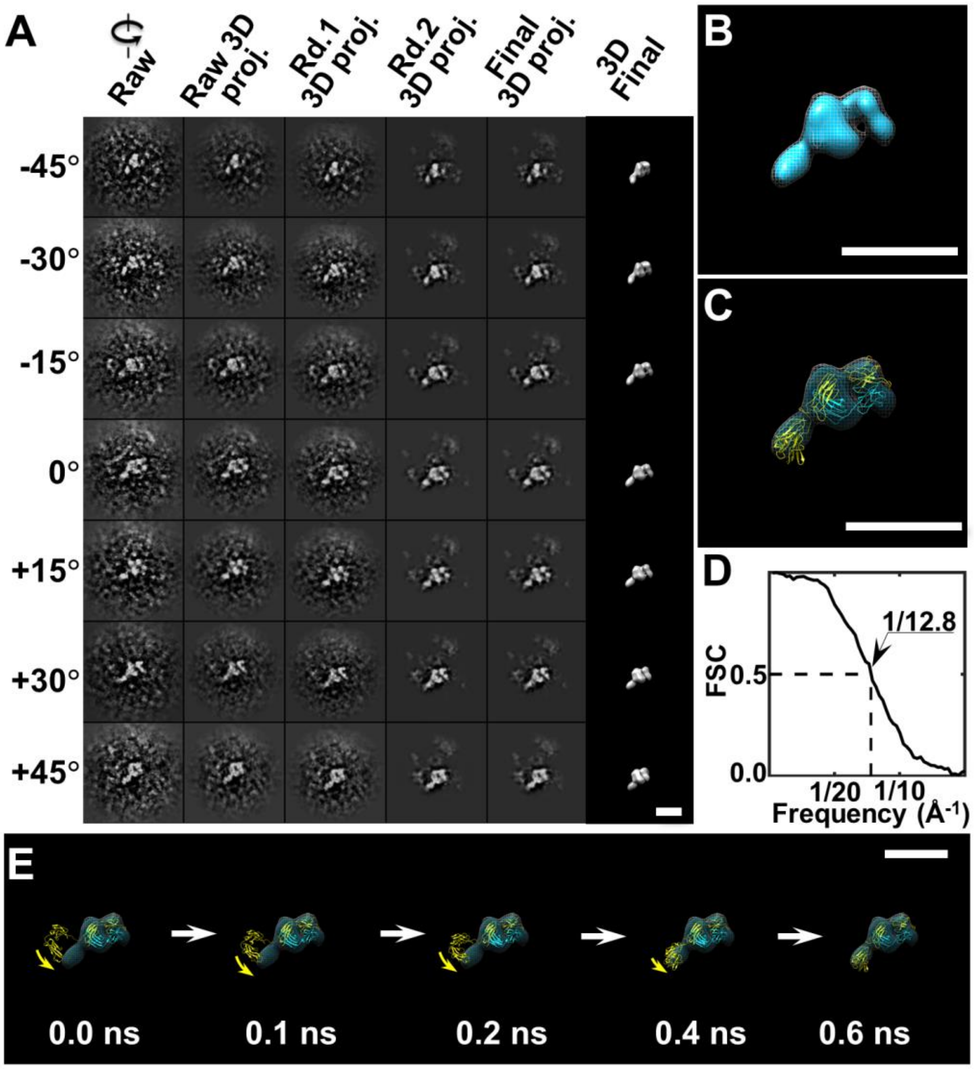

Supplementary Fig. 43 | 3D IPET reconstruction of the eighth i-shaped half-IgG by IPET. (A) Seven representative tilt images of an individual i-shaped particle are displayed in the first column from the left. Using IPET, the tilt images (after CTF correction) were gradually aligned to a common center for 3D reconstruction via iterative refinement. Projections of raw, intermediate and final 3D reconstructions at the corresponding tilt angles are displayed in the next five columns according to their corresponding tilt angles. **(B)** The final 3D density map. **(C)** The density map was flexibly docked with IgG crystal structure by using TMD simulation. **(D)** FSC analyses (from two density maps reconstructed from odd and even numbers of tilt images) showed that the resolution of the final 3D density map was ~12.8 Å. **(E)** Five snapshots illustrated the conformational changes of IgG model during TMD simulation. Scale bars=10 nm.

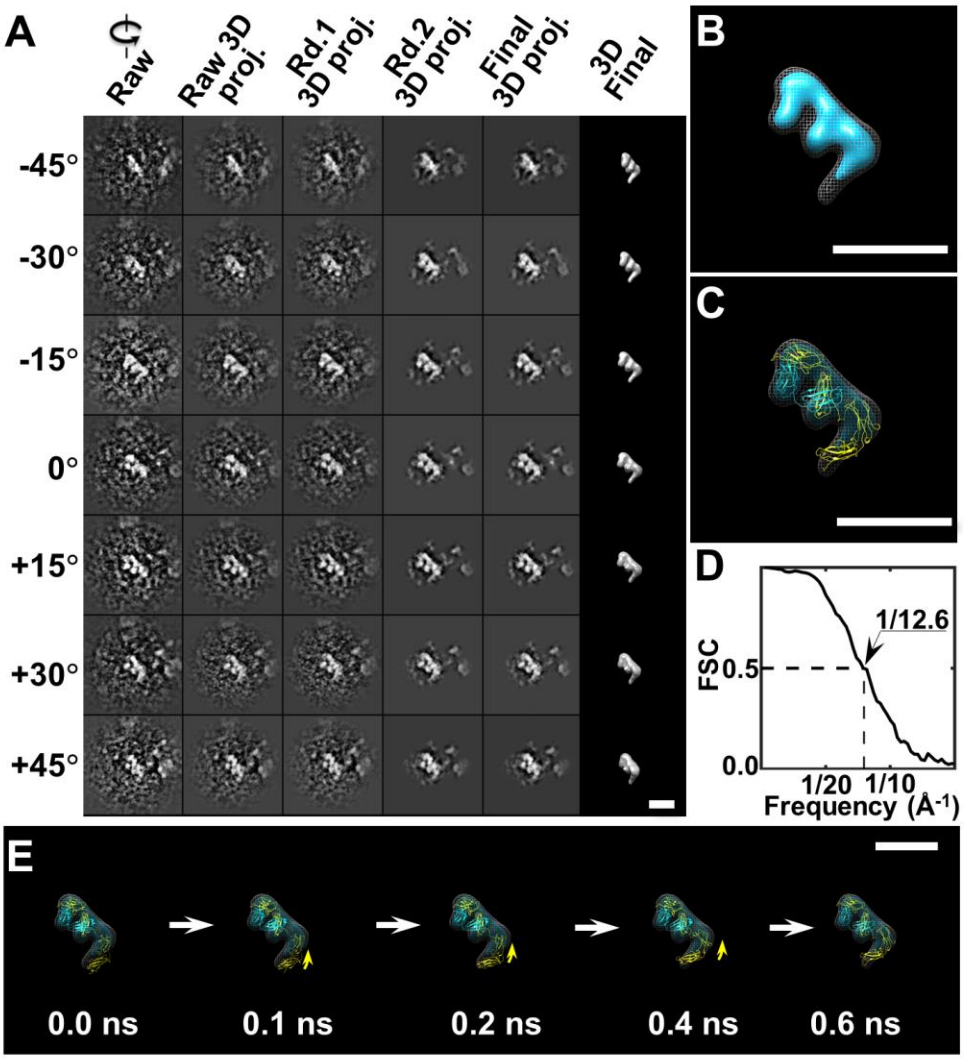

Supplementary Fig. 44 | 3D IPET reconstruction of the ninth i-shaped half-IgG by IPET. (A) Seven representative tilt images of an individual i-shaped particle are displayed in the first column from the left. Using IPET, the tilt images (after CTF correction) were gradually aligned to a common center for 3D reconstruction via iterative refinement. Projections of raw, intermediate and final 3D reconstructions at the corresponding tilt angles are displayed in the next five columns according to their corresponding tilt angles. **(B)** The final 3D density map. **(C)** The density map was flexibly docked with IgG crystal structure by using TMD simulation. **(D)** FSC analyses (from two density maps reconstructed from odd and even numbers of tilt images) showed that the resolution of the final 3D density map was ~12.6 Å. **(E)** Five snapshots illustrated the conformational changes of IgG model during TMD simulation. Scale bars=10 nm.

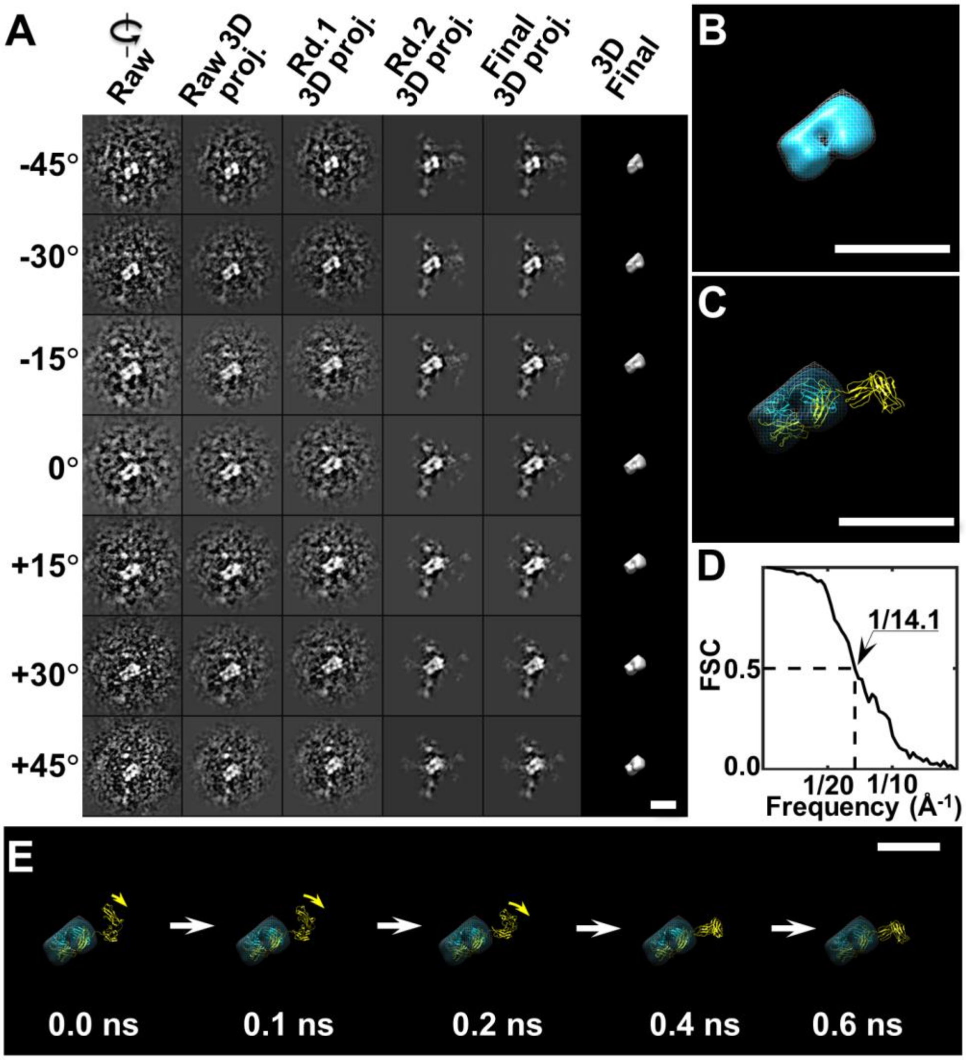

Supplementary Fig. 45 | 3D IPET reconstruction of the tenth i-shaped half-IgG by IPET. (A) Seven representative tilt images of an individual i-shaped particle are displayed in the first column from the left. Using IPET, the tilt images (after CTF correction) were gradually aligned to a common center for 3D reconstruction via iterative refinement. Projections of raw, intermediate and final 3D reconstructions at the corresponding tilt angles are displayed in the next five columns according to their corresponding tilt angles. **(B)** The final 3D density map. **(C)** The density map was flexibly docked with IgG crystal structure by using TMD simulation. **(D)** FSC analyses (from two density maps reconstructed from odd and even numbers of tilt images) showed that the resolution of the final 3D density map was ~14.1 Å. **(E)** Five snapshots illustrated the conformational changes of IgG model during TMD simulation. Scale bars=10 nm.

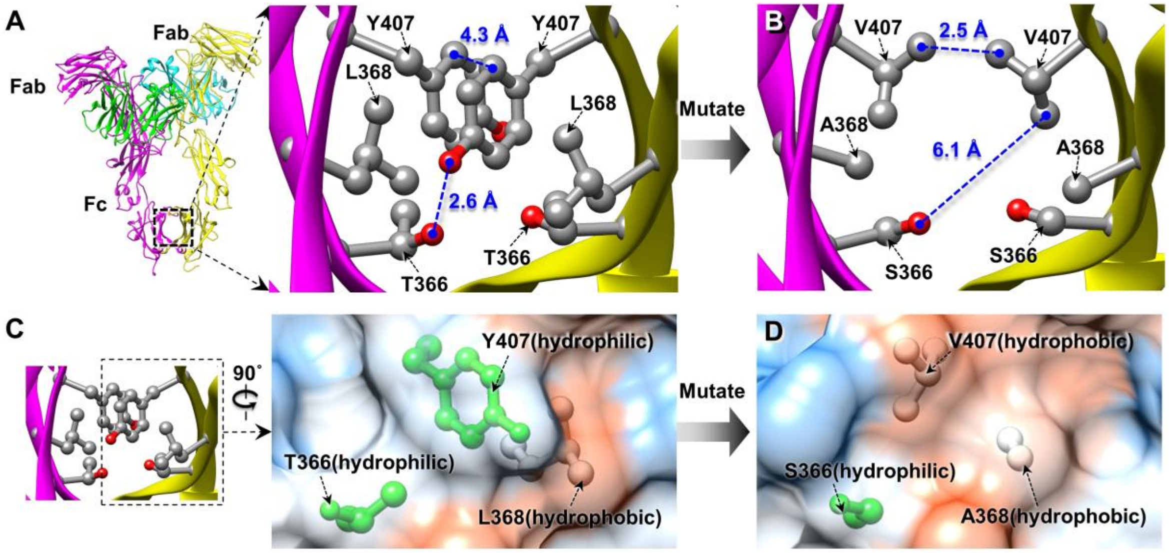

Supplementary Fig. 46 | The structural basis of CH_3-CH_3 interactions. (A) Overall view of the crystal structure of a native IgG (left, PDB entry 1HZH) and zoom-in view of the interface between two CH_3 domains (right). Residues T366, L368 and Y407, which are involved in hole mutations, are shown as ball-and-stick models and colored by element using Chimera. The protein is in ribbon representation, with heavy chains in yellow and magenta and light chains in cyan and green. **(B)** Zoom-in view of the interface between two CH_3 domains of a homology model of IgG homodimer (constructed based on crystal structures of normal IgG, and F_c fragments with hole mutations (PDB entry 1HZH and 4NQT); see Methods.) **(C)** Interface between two CH_3 domains of normal IgG (left) and zoom-in view of the protein surface near residues T366, L368 and Y407 (right). In the zoom-in view, residues T366, L368 and Y407 are shown as ball-and-stick models with hydrophobic residues in white and hydrophilic residues in green. Protein is shown as a transparent surface with most hydrophobic areas in orange and most hydrophilic areas in blue. **(D)** Hydrophobicity of the protein surface near residues S366, A368 and V407 with hole mutations.

Supplementary Video 1 | IPET 3D reconstruction and modeling of an individual X-shaped particle.
